# Supplementary material for: Ancient Plasmodium genomes shed light on the history of human malaria
Source: Nature. 2024 Jun 12;631(8019):125–33. doi: 10.1038/s41586-024-07546-2 (PMC11222158; doi:10.1038/s41586-024-07546-2)
Supplement: Supplementary file 1 — This file contains Supplementary Methods 1–12 and Supplementary Notes 1–13. [file 41586_2024_7546_MOESM1_ESM.pdf]

nature portfolio

https://doi.org/10.1038/s41586-024-07546-2

Supplementary information

Ancient *Plasmodium* genomes shed light on the history of human malaria

In the format provided by the authors and unedited

Supplementary Information

Ancient *Plasmodium* genomes shed light on the history of human malaria

AUTHOR LIST

Megan Michel, Eirini Skourtanioti, Federica Pierini, Evelyn K. Guevara, Angela Mötsch, Arthur Kocher, Rodrigo Barquera, Raffaela A. Bianco, Selina Carlhoff, Lorenza Coppola Bove, Suzanne Freilich, Karen Giffin, Taylor Hermes, Alina Hiß, Florian Knolle, Elizabeth A. Nelson, Gunnar U. Neumann, Luka Papac, Sandra Penske, Adam B. Rohrlach, Nada Salem, Lena Semerau, Vanessa Villalba-Mouco, Isabelle Abadie, Mark Aldenderfer, Jessica F. Beckett, Matthew Brown, Franco G. R. Campus, Tsang Chenghwa, María Cruz Berrocal, Ladislav Damašek, Kellie Sara Duffett Carlson, Raphaël Durand, Michal Ernée, Cristinel Fântăneanu, Hannah Frenzel, Gabriel García Atiénzar, Sonia Guillén, Ellen Hsieh, Maciej Karwowski, David Kelvin, Nikki Kelvin, Alexander Khokhlov, Rebecca L. Kinaston, Arkadii Korolev, Kim-Louise Krettek, Mario Küßner, Luca Lai, Cory Look, Kerttu Majander, Kirsten Mandl, Vittorio Mazzarello, Michael McCormick, Patxuka de Miguel Ibáñez, Reg Murphy, Rita E. Németh, Kerkko Nordqvist, Friederike Novotny, Martin Obenaus, Lauro Olmo-Enciso, Päivi Onkamo, Jörg Orschiedt, Valerii Patrushev, Sanni Peltola, Alejandro Romero, Salvatore Rubino, Antti Sajantila, Domingo C. Salazar García, Elena Serrano, Shapulat Shaydullaev, Emanuela Sias, Mario Šlaus, Ladislav Stančo, Treena Swanston, Maria Teschler-Nicola, Frederique Valentin, Katrien Van de Vijver, Tamara L. Varney, Alfonso Vigil-Escalera Guirado, Christopher K. Waters, Estella Weiss-Krejci, Eduard Winter, Thiseas C. Lamnidis, Kay Prüfer, Kathrin Nägele, Maria Spyrou, Stephan Schiffels, Philipp W. Stockhammer, Wolfgang Haak, Cosimo Posth, Christina Warinner, Kirsten I. Bos, Alexander Herbig, Johannes Krause

TABLE OF CONTENTS

SUPPLEMENTARY METHODS **4**
Supplementary Methods 1: Custom Screening Database Design 4
Supplementary Methods 2: Hybridization Capture Evaluation 5
Supplementary Methods 3: Laboratory Processing for Human Genomic Analysis 5
Supplementary Methods 4: Mitochondrial Capture Quality Control 6
Supplementary Methods 5: Nuclear Capture Quality Control 7
Supplementary Methods 6: Modern *Plasmodium* Mitochondrial Datasets 9
Supplementary Methods 7: Modern *Plasmodium* Nuclear Datasets 9
Supplementary Methods 8: Outgroup Selection 11
Supplementary Methods 9: Downsampling Simulations 12
Supplementary Methods 10: Human Population Genetic Analysis 12
Supplementary Methods 11: Malaria Preservation in Skeletal Material 16
Supplementary Methods 12: Bayesian Phylogenetic Analysis and Molecular Dating 16
SUPPLEMENTARY TEXT **18**
Supplementary Note 1. Archaeological Background 18
1. Archaeological Site and Sample Information for *P. falciparum* Endemic Sites 19
1.1.1 Bourges, Monin 19
1.1.2 Castelsardo 20
1.1.3 Chokhopani 21
1.1.4 Corona Moltana/Zarau 22
1.1.5 Galleon Beach 22
1.1.6 Göttlesbrunn 23
1.1.7 Heping Dao B 23
1.1.8 Lippstadt 24
1.1.9 Paris Réaumur-Sébastopol 25
1.1.10 Torčec, Crkvišće 25
2. Archaeological Site and Sample Information for *P. vivax* Endemic Sites 26
1.2.1 Cueva de las Lechuzas 26
1.2.2 Gâmbaş – Site 3 26
1.2.3 Gars/Thunau 27
1.2.4 Gózquez 30
1.2.5 Gundorovka 32
1.2.6 Laguna de los Cóndores 32
1.2.7 Leubingen 33
1.2.8 Lungi Tepa 34
1.2.9 Mikulovice 34

1.2.10 S’Orcu ‘e Tueri (Perdasdefogu) 35
1.2.11 Tarquinia 35
1.2.12 Thebes 36
1.2.13 Voivodeni 37
1.2.14 Volosovo 38
3. Archaeological Site and Sample Information for *P. malariae* Endemic Sites 38
1.3.1 Sosnovaya griva 3 38
4. Archaeological Site and Sample Information for Endemic Sites with Multiple Plasmodium Species 39
1.4.1 Mechelen, St. Rombout’s cemetery 39
Supplementary Note 2. Mitochondrial Capture Analysis 41
Supplementary Note 3: Nuclear Capture Analysis 43
Supplementary Note 4: Nuclear Downsampling Simulations 45
Supplementary Note 5: *P. vivax* Population Genetic Analysis 45
Supplementary Note 6: *P. vivax* Population Structure Within Latin America 47
Supplementary Note 7: *P. falciparum* Population Genetic Analysis 48
Supplementary Note 8: Human Population Genomics of LDC020 51
Supplementary Note 9: High-Altitude Malaria- Epidemiological and Climatological Considerations 51
Supplementary Note 10: Human Population Genomic Analysis of Individuals from Mechelen, Belgium 52
Supplementary Note 11: Factors Impacting *Plasmodium* spp. aDNA Recovery in High-Altitude Contexts 55
Supplementary Note 12: Malaria Preservation in Skeletal Material 56
Supplementary Note 13: Bayesian Molecular Dating Using BEAST 57
SUPPLEMENTARY TEXT REFERENCES 59

SUPPLEMENTARY METHODS

Supplementary Methods 1: Custom Screening Database Design

To maximize the sensitivity and specificity of our metagenomic analysis while reducing computational time, we designed a custom screening database including both *Plasmodium* species of interest and taxa with significant sequence similarity that could generate false positive identifications in metagenomic analyses, hereafter referred to as false positive taxa. False positive taxa were identified using a two-step approach, in which we (1.) simulated ancient *Plasmodium* reads from modern references and (2.) identified taxa with significant sequence similarity in a custom database containing all sequences present in the NCBI nucleotide database as of Oct. 26, 2017, hereafter referred to as the MALT full-nt database.

Ancient *Plasmodium* reads were simulated using the following complete and chromosome level assemblies, which were downloaded from the NCBI GenBank database on January 27, 2021: *Plasmodium falciparum*- GCA_000002765.3, GCA_900632045.1[91,92,102]; *Plasmodium vivax*- GCA_900093555.2[93]; *Plasmodium ovale*- GCA_900090025.2, GCA_900090035.2[103]; and *Plasmodium malariae*- GCA_900090045.1[103]. For each assembly, we retained scaffolds corresponding to the 14 nuclear chromosomes, as well as the mitochondrion and apicoplast genomes, where available. Next, a custom script was used to simulate reads of 50 bp in length with 1 bp tiling in FASTQ format. Duplicate simulated reads were removed using SeqKit (v. 2.4.0) (rmdup -s)[104]. Simulated reads were mapped to the human genome HG19 using the nf-core/eager (v. 2.3.1) implementation of BWA aln (v. 0.7.17) with mapping parameters -n 0.01 and -l 16; Samtools (v. 1.9) was used to filter alignments with quality threshold 0[64,66,83]. Unmapped reads were saved in FASTQ format using the nf-core/eager flag --bam_umapped_type fastq. Finally, simulated reads were further filtered to remove low-complexity sequences using the PRINSEQ-lite (v. 0.20.4) implementation of the dust algorithm with a complexity threshold of 7 (-lc_threshold) and quality scaling disabled (-qual_noscale)[67]. To identify false positive taxa, MALT (v. 0.4.0) was used to query simulated *Plasmodium* reads against the MALT full-nt database[68]. Semi-global alignment was performed using BlastN mode (-at SemiGlobal, -m BlastN), and binning was performed with the following parameters: minimum support value for the LCA algorithm of 1 (-sup 1), maximum alignments per query of 100 (-mq 100), top percent value for the LCA algorithm of 1 (-top 1), and minimum percent identity used by the LCA algorithm of 90.0 (-mpi 90.0). Replicate query cache was enabled using the flag --replicateQueryCache. Next, we filtered the tab-delimited alignments using a custom python script to identify false positive taxa for inclusion in the *Plasmodium* screening database (db_false_pos_taxa_id.py; https://github.com/meganemichel/plasmodium_project_scripts). Briefly, out of 414,785,536 alignments of our simulated reads to taxa in the MALT full-nt database, we retained 317,837,623 alignments (76.6%) with over 90% sequence identity. Taxa within the *Plasmodium* genus were excluded, leaving a total of 1,203,276 alignments. In many cases, a single simulated read aligned to more than one reference from the same species; to eliminate this redundancy, we retained only the highest-scoring alignment for each read-taxon combination. Employing these filtering criteria, we generated a final set of false positive taxa including 44,173 references from 15,053 unique taxa (**Supplementary Table 9)**. Finally, we combined these false positive taxa with a small number of high-quality reference sequences from our *Plasmodium* species of interest (**Supplementary Table 10**). The custom *Plasmodium* database was constructed using

MALT-build (v. 0.4.0) with sequence type (-s) set to DNA[68]. We used the following accession-to-taxonomy file compiled in Jan. 2021 and available for download on the MEGAN6 download page: megan-nucl-Jan2021.db.zip.

Supplementary Methods 2: Hybridization Capture Evaluation

To identify potential off-target matches, we screened our bait sets against the full-nt database described above (**Supplementary Methods 1)** using MALT (v. 0.5.2 for mitochondrial probes, v. 0.4.0 for nuclear probes). The following parameters were used for read alignment and taxonomic binning: BlastN mode, semiglobal alignment, minimum support value for the LCA algorithm of 1 (-sup 1), maximum alignments per query of 100 (-mq 100), top percent value for the LCA algorithm of 1 (-top 1), and minimum percent identity used by the LCA algorithm (-mpi) set to 90[68].Taxonomic binning resulted in fewer than 0.001% and 0.22% of the mitochondrial and nuclear probes assigned outside of the *Plasmodium* genus, suggesting that capture of off-target sequences should be minimal with these reagents. To assess breadth of coverage on the target genomes, mitochondrial baits were mapped to the following references: *P. falciparum* (LR605957.1), *P. vivax* (LT635627.1), *P. malariae* (LT594637.1), and *P. ovale* (LT594520.1); nuclear probes were mapped to the autosomes of *P. falciparum* (GCA_000002765.3) and *P. vivax* (GCA_900093555.1) (**Extended Data Figure 7**)[91–93]. For each probe set, mapping was performed using BWA aln (v. 0.7.17) with strict parameters (-n 0.1, -l 32), and alignments were filtered using Samtools (v. 1.12) with a quality threshold (-q) of 37[64,66]. Depth of coverage and nucleotide composition were assessed using BEDtools (v. 2.25.0) with 10 bp and 5000 bp windows for the mitochondrial and nuclear bait sets, respectively[90]. Finally, following deduplication using Picard MarkDuplicates (http://broadinstitute.github.io/picard), we assessed the breadth of nuclear probe coverage using Qualimap (v. 2.2.2)[105].

Supplementary Methods 3: Laboratory Processing for Human Genomic Analysis

Forty ancient individuals from the site of St. Rombout's cemetery in Mechelen, Belgium and one ancient individual from Laguna de los Cóndores in Peru were selected for human population genetic analysis (**Supplementary Table 7**). All sampling, extraction, and library preparation steps were performed in a dedicated clean room facility at the Max Planck Institute of Geoanthropology (formerly MPI for the Science of Human History) in Jena, Germany. For each individual, a tooth was sectioned at the enamel-dentin junction, and between 10.5 and 65.5 mg of powder was obtained from the dental pulp chamber as previously described (protocol: https://doi.org/10.17504/protocols.io.bqebmtan). For 12 samples, DNA was extracted following a silica column-based protocol (https://doi.org/10.17504/protocols.io.baksicwe)[74], while the remaining extracts were produced using a modified version of a previously-published bead-based method[75]. Briefly, lysates were prepared by adding 1 ml of extraction buffer (0.45 M EDTA, pH 8.0, 0.25 mg/ml proteinase K, 0.05% Tween-20) to the sample material in 2.0-ml Eppendorf Lo-Bind tubes and rotating the tubes at 37°C for approximately 16 hours[74,75]. DNA was purified from 125 µl lysate using an automated liquid handling system (Bravo NGS Workstation B, Agilent Technologies) as described elsewhere[75]. For LDC020, a double-stranded UDG-half treated library was produced as previously described

(http://doi.org/10.17504/protocols.io.bmh6k39e)[76]. For St. Rombout's Cathedral, 11 double-stranded non-UDG treated libraries were generated (protocol: http://doi.org/10.17504/protocols.io.bakricv6)[77], while the remaining 29 libraries were produced following a modified version of a previously-published single-stranded non-UDG protocol (see **Methods: Laboratory Processing**)[78,79]. Indexed libraries were amplified to plateau by running 35 PCR cycles, followed by an additional reamplification in which each library was diluted 1:2 with water and used as a DNA matrix for PCR with 15 cycles. All libraries were captured using a bait set targeting approximately 1.2 million human SNP positions as previously described[72,81,82]. Samples from St. Rombout's were sequenced on a HiSeq4000 with 75 bp single-end sequencing chemistry (1 x 76 + 8 + 8 cycles); LDC020 was sequenced on a HiSeq4000 with both 75 bp paired-end sequencing chemistry (2 x 76 + 8 + 8 cycles) and 50 bp paired-end sequencing chemistry (2 x 51 + 8 + 8 cycles).

Supplementary Methods 4: Mitochondrial Capture Quality Control

To investigate the species composition of *Plasmodium* MT capture datasets, preprocessed reads were competitively mapped to a concatenated reference containing the *P. falciparum* (LR605957.1), *P. vivax* (LT635627.1), and *P. malariae* (LT594637.1) mitochondrial genomes. Libraries prepared using non-UDG protocols (both single-stranded and double-stranded) were aligned using BWA aln (v. 0.7.17) with loose mapping parameters (-n 0.01, -l 16)[64]. For single-stranded UDG-half treated libraries, double-stranded UDG-half treated libraries, and sequences from the non-UDG treated Ebro Delta dataset, reads were clipped using fastp (v. 0.20.1) prior to mapping[85]. We removed 2 bp from both the 5' and 3' ends, after which reads were mapped using BWA aln (v. 0.7.17) with strict parameters (-n 0.1, -l 32)[64]. For all libraries, we filtered alignments using Samtools (v. 1.12) with a quality threshold (-q) of 37, after which duplicates were removed using Picard MarkDuplicates with default parameters (http://broadinstitute.github.io/picard/)[66].

Visual inspection of aligned datasets revealed several short genomic regions that appear particularly prone to mismapping:

**Species**	**Reference**	**Region Start**	**Region End**
P. falciparum	LR605957.1	5710	5752
P. vivax	LT635627.1	1000	1060
P. malariae	LT594637.1	5240	5240

Prior to deduplication, these regions show stacking of reads, resulting in significantly higher coverage than surrounding segments. Furthermore, across sequencing datasets, these regions exhibit a high number of mismatches and/or heterozygous positions, suggesting possible mismapping from environmental contaminants. Consistent with this hypothesis, for both *P. falciparum* and *P. vivax*, these regions encode portions of rRNAs, which may be conserved across a wide range of potentially contaminating taxa. Therefore, we have excluded these regions from all subsequent analyses.

Following deduplication, bams were processed to extract reads competitively aligned to each species. For each reference, we computed the number of aligned reads mapping outside of the problematic regions identified above using BEDtools (v. 2.25.0) intersect with the flag -v; problematic regions were supplied as a BED file (-b), and the resulting intersected alignments were output in bam format (-wa) (**Supplementary Table 14**)[90]. Finally, for each of the competitive alignments, we repeated the Samtools filtering step with a mapping quality filter (-q) of 0, extracted reads mapping to each reference species, and counted the number of alignments outside of problematic regions. We used these values to compute a mapping specificity metric for each species as follows, where m is the specificity score (**Supplementary Table 14**):

$$m = \frac{alignment\ \#, mapq0}{alignment\ \#, mapq37 + 1}$$

Finally, to evaluate characteristic patterns of aDNA damage, we re-mapped preprocessed reads from UDG-half treated libraries and the Ebro1944 datasets without trimming bases from the 5’ and 3’ ends; all other parameters were identical to those described above for non-UDG treated libraries (loose mapping parameters: -n 0.01 -l 16). We combined these bams with data from non-UDG treated libraries and extracted alignments falling outside of problematic regions, as described above. For reads mapping to each species, ancient DNA damage patterns were evaluated using DamageProfiler (v 1.1, -l 100, -yaxis_dp_max 0.30) (**Supplementary Table 14**)[106]. Finally, ancient *Plasmodium* damage rates were compared to damage patterns on reads mapping to the human genome. For all ancient libraries, unclipped, adapter-trimmed reads were mapped to the human reference genome hs37d5 using BWA aln (v. 0.7.17) with the following parameters: -n 0.01, -l 16500, -k 2, and -o 2[64]. Alignments were filtered using Samtools (v. 1.12) with a quality threshold (-q) of 25, duplicates were removed using Picard MarkDuplicates (http://broadinstitute.github.io/picard/) with default parameters, and damage rates were assessed with DamageProfiler (v. 0.4.9) (**Supplementary Table 14**)[66,106].

Supplementary Methods 5: Nuclear Capture Quality Control

As for the mitochondrial captures, nuclear capture datasets were competitively mapped to a concatenated reference containing the human genome hs37d5 as well as the 14 nuclear chromosome scaffolds of both *P. falciparum* (3D7, GCA_000002765.3) and *P. vivax* (PvP01, GCA_900093555.1)[91–93]. Single- and double-stranded non-UDG treated libraries were mapped using BWA aln (v. 0.7.17) with loose parameters; both single- and double-stranded UDG-half treated libraries and the Ebro Delta datasets were trimmed (2 bp from the 5’ and 3’ ends) and mapped with strict parameters, as described for the mitochondrial capture experiments[64]. Samtools (v. 1.12) was used for alignment filtering with a quality threshold of 37, and deduplication was performed using Picard MarkDuplicates with default parameters (http://broadinstitute.github.io/picard/)[66]. Finally, we evaluated coverage of the target positions using the nf-core/eager implementation of BEDtools (v. 2.30.0), supplying the list of target SNP positions for each species in BED format[83,90].

Several metrics were used to evaluate the success of the nuclear capture experiments. First, we counted alignments competitively mapped to the chromosomal scaffolds of each species (**Supplementary Table 15**). Next, for each species, we used BEDtools intersect (v.

2.25.0) to extract and count alignments overlapping target SNP positions (**Supplementary Table 15**)[90]. The percentage of on-target alignments was computed by dividing the number of reads overlapping target SNPs by the total number of alignments mapping to each species. We next assessed the output of BEDtools coverage to determine the number of target SNP positions per sample covered by at least one read; we also computed the percentage of covered target SNPs belonging to each species (**Supplementary Table 15**)[90]. Finally, we repeated the Samtools filtering step with a mapping quality filter (-q) of 0. Counts of alignments competitively mapped to each reference species were used to compute a mapping specificity metric as described for the mitochondrial analysis:

$$m = \frac{alignment\ \#, mapq0}{alignment\ \#, mapq37 + 1}$$

Next, in order to evaluate aDNA damage patterns in our ancient datasets, we re-mapped preprocessed, unclipped reads from UDG-half treated libraries and the Ebro1944 dataset using loose mapping parameters, as described for the mitochondrial analysis. We combined these data with non-UDG treated libraries already processed with loose mapping parameters and extracted alignments overlapping target SNP positions. Ancient DNA damage patterns were evaluated using DamageProfiler (v. 1.1, -l 100, -yaxis_dp_max 0.30) in reads mapping to each species (**Supplementary Table 15**)[106]. Finally, for each *Plasmodium* nuclear capture dataset, we extracted off-target reads competitively mapped to the 23 human autosomal scaffolds, the X/Y chromosomes, and the mitochondria. We used DamageProfiler to compute C to T substitution rates on reads mapping to the human genome as described above, and compared these values to ancient *Plasmodium* damage rates obtained from the same libraries (**Supplementary Table 15**)[106].

Finally, we employed a MALT-filtering step to further verify that the reads mapped to each species derive from endogenous ancient *Plasmodium* infections rather than spurious alignments from environmental contaminants. For each nuclear capture experiment, we reprocessed the raw sequencing data using nf-core/eager v. 2.4.5; adapter trimming, read collapsing, and lane merging were performed as described above, except that we omitted the fastp damage-clipping step[83]. Reads were then mapped to the human reference genome hs37d5 using BWA aln (v. 0.7.17) with the following parameters: -n 0.01 -l 1024 -k 2 -o 2[64]. Alignments were filtered with Samtools (v. 1.12) using a mapping quality threshold (-q) of 0[66]. MALT (v. 0.4.1) was used to query unmapped reads against a custom database containing all sequences present in the NCBI nucleotide database as of Oct. 26, 2017[68]. MALT performed semi-global alignment of unmapped reads using BlastN mode (-at SemiGlobal, -m BlastN); binning parameters followed those described above (**Custom Screening Database Design)**, except that the minimum percent identity used by the LCA algorithm was set to 85.0 (-mpi 85.0). rma6 files were visualized manually in MEGAN, and we extracted the names of summarized reads from both the Laverania and *P. vivax* nodes[69]. Using seqtk subset (v. 1.2-r94), we extracted the original reads from the raw FASTQ files and repeated the nuclear capture preprocessing and competitive mapping steps on these subsampled datasets (https://github.com/lh3/seqtk). Counts of reads competitively mapped to each species and target SNPs covered in the MALT-filtered datasets were generated for comparison with the unfiltered datasets (**Supplementary Table 15**).

Supplementary Methods 6: Modern *Plasmodium* Mitochondrial Datasets

Comparative modern *P. falciparum*, *P. vivax*, and *P. malariae* mitochondrial datasets were identified, downloaded, genotyped, and merged with ancient data using a custom snakemake script (plasmodium_mito.smk, https://github.com/meganemichel/plasmodium_project_scripts/). First, we used the python Bio.Entrez package to query the NCBI Nucleotide database for records matching a user-supplied search term[107]. For *P. falciparum* and *P. vivax,* our search terms were as follows:

<u>*Plasmodium falciparum*</u>

```
"Plasmodium falciparum"[Organism] AND ("mitochondrial"[All Fields] OR
"mitochondrion"[All Fields] OR "mitochondrial"[All Fields]) AND "complete"[TI]
AND ("5000"[SLEN] : "999999"[SLEN])'
```

<u>*Plasmodium vivax*</u>

```
"Plasmodium vivax"[Organism] AND ("mitochondrial"[All Fields] OR
"mitochondrion"[All Fields] OR "mitochondrial"[All Fields]) AND "complete"[TI]
AND ("5000"[SLEN] : "999999"[SLEN])'
```

Next, we downloaded relevant sequences (**Supplementary Table 13**) in multifasta and genbank format, from which relevant information was extracted to generate a tsv metadata file. Strains derived from nonhuman primates were excluded from downstream analysis. We then split the multifasta by mitochondrial sequence and used a custom script to simulate 50 bp reads with 1 bp tiling for each genome. Finally, we used nf-core/eager to map, genotype, and analyze modern *Plasmodium* mitochondrial genomes alongside ancient genotyped datasets (**Methods: *Plasmodium* Mitochondrial Genome Analysis**).

Supplementary Methods 7: Modern *Plasmodium* Nuclear Datasets

Comparative modern *P. falciparum* data were obtained from the Pf6 data release of the MalariaGEN *P. falciparum* Community Project (https://www.malariagen.net/resource/26), which includes nuclear genotypes and metadata associated with 7,113 *P. falciparum* isolates from 29 countries[38]. We used a custom python script to filter the Zarr format SNP data and convert them to EIGENSTRAT format for downstream analysis (ZarrToEigenstrat.py, https://github.com/meganemichel/plasmodium_project_scripts/). Briefly, we first filtered genotyped variants to include only biallelic SNP positions passing MalariaGEN quality control metrics and with a VQSLOD score greater than 0. Next, we excluded *Plasmodium* clones that were flagged in the published dataset due to duplicate sampling, low coverage, potential mislabeling and mixed-species infections, as well as sampled individuals who acquired their infection while traveling, which increases uncertainty surrounding the geographic origin of sampled strains. The preceding quality control steps resulted in the retention of 5,965 *P. falciparum* strains from 29 countries genotyped at 1,624,267 biallelic SNP positions. We retained the majority of population assignments from the *P. falciparum* Community Project for downstream analysis; however, as we observed evidence for phylogeographic substructure in strains from Oceania (OCE), we split this group into two subpopulations: Indonesia (Indonesia) and Papua New Guinea (PNG)[38]. Finally, despite constituting an important center of *P.*

falciparum endemicity, the MalariaGEN Pf6 release lacks genotype data from India. To partially compensate for this sampling bias, we downloaded publicly available shotgun data from 5 *P. falciparum* strains sampled from hospitalized patients in Goa, India (SRR3575059, SRR3575060, SRR3575061, SRR3575062, SRR3575063)[45]. Read preprocessing, mapping, and genotyping of these samples followed the methods outlined for the Ebro Delta datasets (**Methods**), except that we omitted the damage trimming step.

While all of the human stages of the *Plasmodium* life cycle are haploid, the MalariaGEN *P. falciparum* Community Project publishes diploid genotype calls[38]. Diploid genotypes can be used to compute within-host diversity; in natural infections, *Plasmodium* spp. are often present as part of strain mixtures, and estimates of within-host diversity provide a useful metric for exploring transmission dynamics[38]. However, calling diploid genotypes requires high coverage data, which are a rarity in studies of ancient DNA. Instead, analyses of ancient human SNP data typically employ pseudohaploid genotyping, in which one allele at each locus is sampled by randomly selecting an alignment overlapping the position of interest. In order to maintain consistency between genotyping of modern and ancient *Plasmodium* strains, we converted the diploid genotypes produced by the MalariaGEN project into haploid data by selecting one of the two possible alleles with a probability equal to the recorded depth of reads supporting the reference and alternate allele, respectively. Genotypes were written in EIGENSTRAT format for downstream analysis.

Similarly, we accessed comparative modern *P. vivax* genome-wide data from the Pv4 data release of the *P. vivax* Genome Variation Project (https://www.malariagen.net/resource/30), which includes genotypes from 1,895 *P. vivax* samples isolated from 27 countries[37]. Data were filtered using a custom python script as described above for *P. falciparum*; strains failing MalariaGEN QC metrics and derived from travelers were excluded, while loci were filtered to include only biallelic SNPs not flagged as low quality in the original data release and having a VQSLOD score greater than 0. Haploid genotype calls were generated for a final set of 911,901 variant positions genotyped in 1,055 *P. vivax* strains from 27 countries, and the results were written in EIGENSTRAT format. For the majority of samples, we retained population annotations published by the *P. vivax* Genome Variation Project[37]. However, as samples from sparsely sampled regions were given the population label 'unassigned' in the original data release, we combine genetic and geographical data to reclassify samples from the following populations: Africa (AF), Ethiopia (ETH), East Asia/Northern Southeast Asia (EA_NSEA), and Bhutan (Bhutan).

Next, we noted that some modern *P. vivax* and *P. falciparum* populations harbor clusters of highly related strains, which exhibit extremely low pairwise mismatch rates. In particular, *P. vivax* populations from Maritime Southeast Asia and South America contain a high proportion of near-clonal individuals, as noted previously[37]. Due to the specificities of the *Plasmodium* life cycle, the frequency of outcrossing is heavily dependent on epidemiological factors such as transmission intensity[108]. In order to control for varying levels of relatedness in our modern comparative datasets, we computed pairwise mismatch rates between all pairs of individuals within each population using the tool pMMRCalculator (Thiseas Christos Lamnidis, https://github.com/TCLamnidis/pMMRCalculator). We defined 0.02 and 0.075 as the minimum pairwise mismatch rates for *P. falciparum* and *P. vivax* populations, respectively. After iteratively removing one related individual until no remaining pairs fell below the defined cutoffs, we retained 4,558 and 906 strains for *P. falciparum* and *P. vivax*, respectively. Finally, we observed a large skew in the number of individuals sampled per *P. falciparum* population. In order to reduce

computational times and eliminate analytical biases that might result from oversampling strains from particular geographical regions, we retained at most 200 randomly selected strains for each *P. falciparum* population, resulting in a final dataset of 1,227 strains (**Extended Data Figure 2**).

Next, we evaluated the overlap between our nuclear capture probes and the high-quality SNPs genotyped in these modern comparative datasets. Using nf-core/eager (v. 2.4.5), we mapped the probe sequences to the 14 nuclear chromosome scaffolds of the *P. falciparum* and *P. vivax* references used in the MalariaGEN analysis pipelines (*P. falciparum* 3D7, GCA_000002765.3; *P. vivax* PvP01, GCA_900093555.1)[83,91–93]. Mapping was performed with BWA aln (v. 0.7.17) with strict mapping parameters (-n 0.1, -l 32), alignments were filtered using Samtools (v. 1.12) with a mapping quality threshold of 0, and duplicates were removed using Picard MarkDuplicates (v. 2.26.0, http://broadinstitute.github.io/picard/)[64,66]. We supplied nf-core/eager with BED files containing the high quality SNP positions genotyped in the modern comparative *P. falciparum* and *P. vivax* datasets, and coverage was assessed using the nf-core/eager implementation of BEDtools (v. 2.30.0) with default parameters[90]. For each species, all positions with non-zero coverage were combined to create target SNP sets of 873,060 and 872,564 positions for *P. falciparum* and *P. vivax,* respectively. Thus, our probes cover 53.8% and 95.7% of high-quality biallelic SNP variants genotyped in the MalariaGEN *P. falciparum* and *P. vivax* datasets, suggesting that coverage is sufficient to utilize these comparative modern data in analysis of our ancient strains.

Finally, we generated site frequency spectra for the *P. falciparum* and *P. vivax* SNP sets to assess the number of variants segregating in our downsampled modern populations. We observe that the proportion of singletons and low frequency variants is higher in *P. falciparum* relative to *P. vivax*. As such variants are not ancestry-informative, we used PLINK (v1.90b6.21, http://pngu.mgh.harvard.edu/purcell/plink/) to remove singletons and nonsegregating sites from our pseudohaploid data (--mac 4), retaining 106,179 (12.2 %) and 419,387 (48.1 %) sites for *P. falciparum* and *P. vivax*, respectively[98]. In all subsequent analyses, we refer to these filtered variant sets as the segregating target sites.

Supplementary Methods 8: Outgroup Selection

We selected *P. praefalciparum* as an appropriate outgroup for *P. falciparum*, while for *P. vivax* we utilized *P. vivax*-like. Genotypes overlapping the target SNP sets were generated for each species as follows. Briefly, we downloaded *P. vivax*-like (GCA_003402215.1) and *P. praefalciparum* (GCA_900095595.1) assemblies from NCBI and extracted scaffolds corresponding to the 14 nuclear chromosomes[14,109]. As described above (**Custom *Plasmodium* Screening Database**), we used a custom script to simulate 100 bp reads with 1 bp tiling in FASTQ format; using BWA aln (v. 0.7.17), we mapped these reads to the corresponding reference genome (*P. falciparum* 3D7, GCA_000002765.3; *P. vivax* PvP01, GCA_900093555.1) with strict mapping parameters (-n 0.1, -l 32)[64]. Alignment filtering and duplicate removal were performed as described above. Finally, target SNP positions for *P. falciparum* and *P. vivax* were supplied in BED and EIGENSTRAT format, and haploid genotypes were called using the nf-core/eager implementation of pileupcaller (v. 1.5.2, https://github.com/stschiff/sequenceTools) with default parameters (--run_genotyping, –genotyping_tool pileupcaller).

Supplementary Methods 9: Downsampling Simulations

To identify appropriate SNP coverage cutoffs for our ancient datasets, we downsampled modern strains to simulate low-coverage ancient samples and evaluated their performance in population genetic analysis. For *P. vivax*, we randomly selected one strain from the following modern populations in the comparative dataset described above: Africa: QM0003-C, Ethiopia: QS0191-C, Latin America: VVX01806, Western Asia: VVX12022, Bhutan: QR0002-CW, East Asia/Northern Southeast Asia: Myanmar_NB-15, Western Southeast Asia: PD0609-C, Eastern Southeast Asia: VVX01673, Maritime Southeast Asia: VVX01987, and Oceania: PJ0030-C. Similarly, we selected one *P. falciparum* strain from each population published in the MalariaGEN *P. falciparum* Community Project: Central Africa: QG0234-C, Eastern Africa: PE0415-C, Western Africa: PA0699-CW, South America: PP0002-C, South Asia: PR0150-C, Eastern Southeast Asia: PH0403-C, Western Southeast Asia: PD0834-C, and Papua New Guinea: PN0135-C. For each strain, we randomly downsampled genotypes from the segregating target SNP set to simulate three coverage levels: 500 SNPs, 1500 SNPs, and 5000 SNPs. We performed 50 replicates per strain/coverage combination.

Using smartPCA (v. 16000), we evaluated the impact of downsampling on population assignment[95,96]. For each coverage level, modern strains were used to compute the principal components, and simulated data were projected onto these axes of variation using the following parameters: lsqproject: YES, outliermode: 2, usenorm: NO. For each modern downsampled strain, we computed a convex hull encompassing the spatial distribution of the 50 replicates using SciPy (v. 1.9)[110].

Supplementary Methods 10: Human Population Genetic Analysis

For a subset of ancient individuals from malaria-positive sites, we explored patterns of genetic ancestry to identify potential migrants and/or admixed individuals. Selected indexed double-stranded (nonUDG or UDGhalf) and single-stranded (nonUDG) libraries were enriched with hybridization capture for the '1240K' SNP panel[72,81,82], after which they were sequenced on a HiSeq4000 Illumina platform with 75 bp single-end sequencing chemistry (**Supplementary Table 7**). Demultiplexed reads were trimmed for residual adapter sequences using LeeHom (v. 1.1.5-ba378b6 and v1.1.5-eb382b3) with the option --ancientdna[65]. Reads were mapped to the human reference genome hs37d5 using BWA aln (v. 0.7.12; -n 0.01 -o 2 -l 16500) and sorted and indexed with Samtools (v. 1.3)[64,66]. The remaining data processing steps were performed using nf-core/eager (v. 2.4.5)[83]. Alignments exceeding 30 bp in length were retained following deduplication using Picard MarkDuplicates (http://broadinstitute.github.io/picard). To reduce genotyping biases caused by ancient DNA damage in double-stranded non-UDG treated libraries, 7 bp were trimmed from the 5' and 3' ends of alignments with trimBam from bamUtil. For double-stranded UDG-half treated libraries, we trimmed 2 bp from the 5' and 3' read ends. Trimmed and untrimmed bams were genotyped *in silico* at the 1240k SNP positions using the nf-core/eager implementation of pileupcaller (https://github.com/stschiff/sequenceTools) with default parameters (pileupcaller_min_map_quality 25, pileupcaller_min_base_quality 30). For single-stranded non-UDG treated libraries, pileupcaller was executed using --singleStrandMode to account for strand-specific damage profiles characteristic of single-stranded libraries. Finally, the ratio of reads mapped to the X and Y chromosomes was computed using Sex.DetERRmine

(v. 1.1.2), and results were compiled using MultiQC (v. 1.3)[111,112]. Nuclear contamination on males was estimated using ANGSD (v0.935)[113]. Mitochondrial contamination was quantified using contaMix (v. 1.0-10) which was run with nf-core/eager as previously described[114], while applying a quality filter of q30 to mtDNA sequences and the option --contammix_trim_bases 10. To test for evidence of European ancestry in LDC020, we first used the Poseidon framework (v. 2.7.1, http://www.poseidon-adna.org) to merge the EIGENSTRAT file generated with pileupcaller with the poseidon package for the Simons Genome Diversity Project (SGDP)[104]. We performed principal component analysis using smartPCA (v. 16000, EIGENSOFT v7.2.1) with the options lsqproject:YES, and numoutlieriter:0[95,96]. Principal components were computed using a set of 118 modern populations (see below), with the ancient individual LDC020 projected onto these axes of variation. Next, we performed a supervised admixture analysis to further test for possible European ancestry in LDC020. After converting our EIGENSTRAT data to PED format, we used PLINK (v1.9, 9 Jan 2018) to filter SNPs with a minor allele frequency below 0.01 (--maf) as well as SNPs in linkage disequilibrium (--indep-pairwise 200 25 0.4)[98]. Using ADMIXTURE (v. 1.3.0), we performed a supervised analysis with 100 bootstrap replicates (-B 100) to model the ancestry of LDC020 using the following six populations: Atayal, French, Kalash, Karitiana, Mbuti, and Papuan[97]. Finally, as a formal test for European admixture we used *qpDstat* to perform F_4-statistics of the form f_4(LDC020, Test; Spanish.DG, Mbuti.DG), where the test set constitutes a selection of contemporary indigenous South American populations.

We also expanded on other reference datasets from the Americas[115–126] to investigate whether the ancestry of this individual originated in other regions such as the Andes or the Amazon. We used this dataset to compute a regional PCA (**Extended Data Figure 9**). To further disentangle the ancestry in LDC020, we performed ancestry modeling with *qpWave* (v. 5.1)[99] with two datasets: Dataset 1, which maximizes the number of SNPs (> 500K SNPs) and overall amount of aDNA data, and Dataset 2, which maximizes the number of sampled individuals and populations from present-day South America cited above (<500 SNPs) (**Supplementary Table 16**), including the Allen Ancient DNA Resource (v54.1)[127,128].

Similarly, to explore the ancestry of ancient individuals from St. Rombout's cemetery in Mechelen (STR), we performed a principal component analysis using smartpca (v. 16000, EIGENSOFT v7.2.1) after merging STR data with modern and ancient populations from the Allen Ancient DNA Resource (AADR, v54.1)[127,128]. Principal components were computed from 1,254 individuals genotyped at the Human Origins SNPs (~0.5M positions), assigned to a set of modern populations listed below, with the options autoshrink:Yes, and numoutlieriter:0[95,96]. Ancient individuals from St. Rombout's cemetery were projected onto these axes of variation (option lsqproject:Yes'), along with other relevant ancient published datasets dating to the same period [59,129–135]. We filtered the eigenvector output file from smartpca (*.evec*) for individuals dating from the last 4,000 years and excluding relatives, duplicates, low-quality, or failed samples using the annotation file of AADR v54.1 and used it a an input for *mobest* software[136], which creates an interpolated ancestry field through space and time and extracts the spatial similarity probability for an ancient individual according to its genetic profile. First, we applied ESPG:4326 latitude-longitude (or 'projected WSS84') to extract a polygon of surface corresponding to Western Eurasia and North Africa. Then, we projected our spatial data by transforming the land outline in the research area from EPSG:4326 to EPSG:3035. In the kernel parameters for the interpolation, we set both *dsx* and *dsy* at 800km and *dt* at 800 years. This value for *dt* is applied by the authors of the method for Europe. We consider it a good option for our dataset as well, given that available ancient data spanning ±500 years around the average

date of the St. Rombout's cemetery individuals (STR) are still scarce. Therefore, we note that it is mainly the genetic data from present-day and medieval Western Eurasia that will inform the ancestry field for the temporal transects of the STR samples. In all cases we defined *search_time* to be in a range of 200 years (i.e., ±100 years) from the mean C14 date of each STR individual.

Finally, we used *qpWave* from Admixtools (version v5.1)[99] to detect the minimum number of gene flows that explains two target populations ('left pops') from a set of reference populations ('right pops'). In this setting the first target was an STR malaria-positive individual (STR016, STR025, STR045, STR067, STR091, STR105, STR129, STR140, STR185, STR248). At the position of the second target we iterated published groups from the last thousand years which -according to genetic similarity from *smartpca* (PC1-2 coordinates) and similarity probability from *mobest*- represent plausible ascending (or descending if present-day) populations for the Mechelen individuals. As right pops we set: Mbuti.DG, South_Africa_2000BP.SG, Morocco_Iberomaurusian, Iran_GanjDareh_N,Israel_C, Russia_Samara_EBA_Yamnaya, Germany_BellBeaker, Spain_Almoloya_Argar, Greece_BA_Mycenaean. Low p values (usual cutoff: <0.05) indicate that more than one stream of ancestry are necessary to explain the two targets from our set of right pops. In these cases, we used the *qpAdm* module of Admixtools to model each STR individual as a linear combination of two sources from the pool of second targets in *qpWave*, and accepted models with p value ≥ 0.05 and positive coefficients. We kept the same setting of 'right pops' for both modules and used the parameter 'allsnps':yes.

Modern Human Populations used in Global PCA Construction (for LDC020):
Adygei.DG, Albanian.DG, Aleut.DG, Altaian.DG, Ami.DG, Armenian.DG, Atayal.DG, Australian.DG, Balochi.DG, BantuHerero.DG, BantuKenya.DG, BantuTswana.DG, Basque.DG, Bengali.DG, Bergamo.DG, Biaka.DG, Bougainville.DG, Brahmin.DG, Brahui.DG, Bulgarian.DG, Burmese.DG, Burusho.DG, Cambodian.DG, Chechen.DG, Chukchi.DG, Czech.DG, Dai.DG, Druze.DG, Dusun.DG, English.DG, Esan.DG, Eskimo_Chaplin.DG, Eskimo_Naukan.DG, Eskimo_Sireniki.DG, Estonian.DG, Even.DG, Finnish.DG, French.DG, Gambian.DG, Georgian.DG, Greek.DG, Han.DG, Hawaiian.DG, Hazara.DG, Hezhen.DG, Icelandic.DG, Igorot.DG, Iranian.DG, Irula.DG, Itelmen.DG, Japanese.DG, Jew_Iraqi.DG, Jew_Yemenite.DG, Jordanian.DG, Ju_hoan_North.DG, Kalash.DG, Kapu.DG, Karitiana.DG, Khomani_San.DG, Khonda_Dora.DG, Kinh.DG, Korean.DG, Kusunda.DG, Lezgin.DG, Luhya.DG, Luo.DG, Madiga.DG, Makrani.DG, Mala.DG, Mandenka.DG, Mansi.DG, Maori.DG, Masai.DG, Mayan.DG, Mbuti.DG, Mende.DG, Mexico_Zapotec.DG, Miao.DG, Mixe.DG, Mixtec.DG, Mongola.DG, Mozabite.DG, Naxi.DG, Orcadian.DG, Oroqen.DG, Palestinian.DG, Papuan.DG, Pathan.DG, Piapoco.DG, Pima.DG, Polish.DG, Punjabi.DG, Quechua.DG, Relli.DG, Russian.DG, Saami.DG, Saharawi.DG, Samaritan.DG, Sardinian.DG, She.DG, Sindhi.DG, Somali.DG, Spanish.DG, Surui.DG, Tajik.DG, Thai.DG, Tlingit.DG, Tu.DG, Tubalar.DG, Tujia.DG, Turkish.DG, Ulchi.DG, Uyghur.DG, Xibo.DG, Yadava.DG, Yakut.DG, Yi.DG, Yoruba.DG[59,137–140].

Modern Human Populations used in Regional PCA Construction (for LDC020):
Bolivian, Cao, Chotuna, Chulucanas, Cocama, Cofan, Cusco, Cusco2, Eten, Huancas, Inga, Kamentsa, Karitiana.DG, KichwaOrellana, LaJalca, LoretoMix, Luya, Narihuala, Olmos, Paran,

Piapoco.DG, Puno, Quechua.DG, Sechura, Surui.DG, Tallan, Tumbes, UtcubambaSouth, Wayku [116,139].

Modern Human Populations used in West Eurasian PCA Construction (for STR):
Abkhasian.HO, Adygei.HO, Albanian.HO, Armenian.HO, Assyrian.HO, Balkar.HO, Basque.HO, BedouinA.HO, BedouinB.HO, Belarusian.HO, Bulgarian.HO, Chechen.HO, Chuvash.HO, Croatian.HO, Cypriot.HO, Czech.HO, Druze.HO, English.HO, Estonian.HO, Finnish.HO, French.HO, Georgian.HO, Greek.HO, Hungarian.HO, Icelandic.HO, Iranian.HO, Italian_North.HO, Italian_South.HO, Jew_Ashkenazi.HO, Jew_Georgian.HO, Jew_Iranian.HO, Jew_Iraqi.HO, Jew_Libyan.HO, Jew_Moroccan.HO, Jew_Tunisian.HO, Jew_Turkish.HO, Jew_Yemenite.HO, Jordanian.HO, Kumyk.HO, Lebanese_Christian.HO, Lebanese.HO, Lebanese_Muslim.HO, Lezgin.HO, Lithuanian.HO, Maltese.HO, Mordovian.HO, Norwegian.HO, Orcadian.HO, Palestinian.HO, Romanian.HO, Russian.HO, Sardinian.HO, Saudi.HO, Scottish.HO, Sicilian.HO, Spanish_North.HO, Spanish.HO, Syrian.HO, Turkish.HO, Ukrainian.HO, Abazin.HO, Armenian_Hemsheni.HO, Avar.HO, Azeri.HO, Circassian.HO, Darginian.HO, Ezid.HO, Ingushian.HO, Kabardinian.HO, Kaitag.HO, Karachai.HO, Kubachinian.HO, Kurd.HO, Lak.HO, Ossetian.HO, Tabasaran.HO, Turkish_Balikesir.HO[99,130,141–143].

Assignment of uniparental haplogroups.

To assign the Y haplogroups to the malaria-positive males, we followed a previously-described semi-automated approach[144], with the addition of a parameter for the single-stranded libraries which depletes deaminated reads due to ancient DNA damage in the same way as the parameter *--singleStrandMod*e in pileupCaller (ignores forward-aligned reads in CT positions and reverse in GA). To analyze mitochondrial DNA data, reads mapping to mitochondrial genomes were extracted from the 1240k dataset using samtools while applying a quality filter of more or equal to q30. Mitochondrial consensus sequences for each sample were called using ivar (v. 1.3)[145] and then analyzed with Haplogrep3 (v3.2.1)[146] to assign the corresponding mtDNA haplogroups.

Biological relatedness

To determine the degree of genetic relatedness between ancient individuals, we applied four different approaches: PMR (originally reported in Kennett *et al.* 2017)[147], READ[148], BREAD[149], and LcMLkin[150] (**Supplementary Table 17**). First, the rate of mismatches among 1240k SNP positions in individual pairs (PMR: pairwise mismatch rate) was calculated, and the coefficient of relatedness estimated as described below. Pairs with less than 2000 overlapping SNP positions were excluded, while the median PMR value (pU = 0.26181) was established as the baseline for unrelated pairs. Pairs with PMR values below the median represent individuals who are likely more closely related. For those pairs, the coefficient of relatedness was quantified using the equation outlined below:

$$cij=(pU-pij)/pT$$

where pij is the pairwise-mismatch rate for the individuals i and j in the pair; pU is the expected PMR for two unrelated individuals (quantified as the median of all of the pij); pT is the expected PMR for identical individuals (found as pU/2). A coefficient of relatedness (cij) of: approximately 1 suggests identical twins or the same individual, around 0.5 suggests first-degree relatedness, and about 0.25 denotes second-degree relatedness among individuals. READ and BREAD are two methods for estimating relatedness that conceptually rely on the same statistic (PMR). Essentially, READ calculates PMR (P_0) on non-overlapping windows on the genome and uncertainty is estimated with a block-jackknife approach producing Z-scores for whether a pair of individuals is related. BREADR instead uses a binomial distribution for PMR values, therefore pairs are returned with a posterior probability distribution for different degrees of relatedness. Both tools were applied using default parameter settings. The fourth method we adopted was LcMLkin. This approach uses genotype likelihoods to quantify the probability that two individuals have zero, one or two alleles identical-by-descent at a random site in the genome, which are reported as the three k-coefficients (k0, k1 or k2). LcMLkin was here applied using the parameter --thin 50000. Although this method alone cannot accurately define beyond third degree on ancient data, it is powerful to detect pairs as more distantly related.

Supplementary Methods 11: Malaria Preservation in Skeletal Material

We assessed differences in *Plasmodium* DNA yield depending on the skeletal element analyzed. We chose to assess preservation of the mitochondrial rather than the nuclear genome given the very low mean nuclear genomic coverage of all *Plasmodium* strains analyzed here. Mean mitochondrial coverage was used as a response variable following log transformation. The *Plasmodium* species and library strandedness were used together with the skeletal element as predictors of the model due to the potential impact of these variables. The two libraries prepared from calcified tissues were excluded. Samples showing indications of mixed infections or *P. malariae* infections were also excluded. Because in some cases several libraries were sequenced from the same individual, we used a mixed effect model as implemented in the R package lme4 with the individual as a random effect[151].

Supplementary Methods 12: Bayesian Phylogenetic Analysis and Molecular Dating

In order to estimate the age of the Latin American *P. vivax* clade, we performed a Bayesian phylogenetic analysis following a previously outlined approach with minor modifications[30]. In addition to LDC020 and Ebro1944, we obtained modern, publicly available whole-genome sequencing data from 15 samples utilized in a previous dating analysis: SAMN02677154, SAMN02677164, SAMN02677167, SAMN03274512, SAMN02677169,

SAMN02677170, SAMN02677171, SAMN02677180, SAMN02677183, SAMN02677184, SAMN02677185, SAMN02677186, SAMN02677187, SAMN02677195, SAMN00710542[30]. Both modern samples and adapter-clipped, damage-trimmed reads from Ebro1944 and LDC020 were mapped to the PvP01 reference genome using the nf-core/eager (v. 2.4.5) implementation of BWA aln (v. 0.7.17) with strict mapping parameters (-n 0.1, -l 32)[64,83,93]. Alignment filtering was performed using Samtools (v. 1.12) with a mapping quality threshold (-q) of 37[66]. Following deduplication with Picard MarkDuplicates (v. 2.26.0; http://broadinstitute.github.io/picard), genotyping was performed using the GATK UnifiedGenotyper (v. 3.5) with default parameters and the output mode 'EMIT_ALL_SITES'[87]. We generated a SNP alignment using a modified version of MultiVCFAnalyzer (v. 0.87.1)[88]; for each sample, we filtered positions with 1.) a GATK genotyping quality below 30, and/or 2.) less than 90% of reads supporting the base call. The minimum coverage required for a base call was set to 1. Finally, we generated a complete deletion alignment using the tool SNP-sites (v. 2.5.1) with the flag -c[152]. The resulting alignment consisted of 3,993 nucleotide positions genotyped in 15 modern and 2 ancient *P. vivax* strains.

Following the approach used in a previous study, we used the tool homoplasyFinder to identify and remove recombinant positions in the resulting SNP alignment (https://github.com/JosephCrispell/homoplasyFinder)[30,153]. As homoplasyFinder requires both a multifasta file and associated phylogeny, we generated a maximum likelihood tree using the software RAxML-NG (v. 1.1)[154]. We performed an all-in-one analysis (--all) with 1000 bootstrap replicates (--bs-trees 1000) using the general nucleotide substitution model with four gamma rate categories and the Stamatakis ascertainment bias correction for invariant sites (GTR+G4+ASC_STAM). Using the best-scoring maximum likelihood tree as input for homoplasyFinder, we generated a homoplasy-stripped SNP alignment in fasta format (--createFasta). Finally, to visualize the impact of homoplasy removal on tree topology, we generated a new maximum likelihood phylogeny with RAxML-NG using the parameters described above[154]. Both phylogenies were visualized using FigTree (v. 1.4.4; https://github.com/rambaut/figtree/releases/tag/v1.4.4).

Due to uncertainty regarding the impact of homoplasy removal on inferred clock rate, we selected a second sample set for molecular dating analysis (**Supplementary Note 13**). Under the rationale that geographically isolated populations should not have a recent history of recombination, we chose one random strain per modern group included in our *P. vivax* population genetic analysis, except for Bhutan- Africa: malagasy19, Ethiopia: QS0125-C, Latin America: PW0033-C, Western Asia: VVX11827, East Asia/Northern Southeast Asia: QN0005-C, Western Southeast Asia: PD0706-C, Eastern Southeast Asia: VVX01747, Maritime Southeast Asia: PY0001-C, and Oceania: PNG58[37]. After obtaining raw sequencing data from the Sequence Read Archive (SRA), we used the nf-core/eager (v 2.4.5) implementation of BWA aln (v.0.7.17) to map both the modern strains and adapter- and damage-trimmed reads from Ebro1944 and LDC020 to the PvP01 reference genome with strict parameters, as described above[64,83,93]. Deduplication, genotyping, and filtering with MultiVCFAnalyzer followed the procedures outlined for the dataset on which we tested homoplasy removal[87,88]. We used the tool SNP-sites (v. 2.5.1) to generate a complete deletion SNP alignment (-c), which consisted of 8,104 sites genotyped across 9 modern and 2 ancient *P. vivax* strains[152].

Prior to pursuing molecular dating with BEAST, we performed root-to-tip regression and date randomization tests to check for the presence of a temporal signal in our dataset. Briefly, modern sample dates were extracted from metadata published by the *P. vivax* genome variation project[37]; tip dates for ancient samples were inferred either from radiocarbon analyses (LDC020)

or from published materials (Ebro1944)[30]. TempEst (v. 1.5.3) was used to infer the best-fitting root of the maximum-likelihood tree described above and to compute a root-to-tip regression[155]. To further validate the suitability of our sample set for molecular dating, we used BEAST (Bayesian Evolutionary Analysis Sampling Trees, v. 2.7.6) to perform a date randomization test with 15 iterations[156]. Using our complete deletion alignment with accurate sampling dates, we estimated the uncorrelated lognormal relaxed clock rate using the GTR substitution model with estimated frequencies and four gamma rate categories, the optimized relaxed clock model as implemented in the ORC BEAST2 package, and the Coalescent Bayesian Skyline model[157]. We set a diffuse uniform prior for the clock rate (0.011- 1.1×10^{-12}, starting value 1×10^{-9}); for all other priors we used the default values employed in BEAUti (v. 2.7.6), and we manually modified the BEAST2 xml file to correct for invariant sites. Finally, we used a custom script (randomise_dates_beast2.py; https://github.com/sebastianduchene/phylo_xml_tools) to generate 15 modified xml files with randomly shuffled sample dates. For all 16 experiments, the MCMC chains were run for 500,000,000 steps, after which we checked that the ESS values of all parameters exceeded 200. Chains that had not reached convergence were extended, after which we checked the ESS values and confirmed convergence via manual inspection of the BEAST trace file in Tracer (v. 1.7.2). We used the program TreeAnnotator (v. 2.7.6) provided with the BEAST2 package to generate a maximum clade credibility tree for the BEAST run with correct tip dates, specifying a burn-in of 10%, a posterior probability limit of 0, and tabulating median node heights. The maximum clade credibility was visualized using FigTree (v. 1.4.4; https://github.com/rambaut/figtree/releases/tag/v1.4.4).

SUPPLEMENTARY TEXT

Supplementary Note 1. Archaeological Background

The following paragraph describes the archaeological context for *Plasmodium*-positive samples reported in this study. Samples were obtained from 26 sites in 16 countries spanning from South America to the eastern Caribbean, northern Africa, Europe, and central and eastern Asia, covering a period between the 4th millennium BCE and the 18th century CE. The site descriptions are in the first instance organized by *Plasmodium* species identified, followed by the particular site names in alphabetical order. Each site description provides basic information about country, region, and geographical position, as well as dating and sample provenance. Radiocarbon dates include the sample ID, sample type, radiocarbon laboratory code, ^{14}C age before present (BP), and calibrated calendar age (2-sigma, i.e., 95% probability). Radiocarbon dates were calibrated using OxCal (v. 4.4) and the IntCal20 calibration curve, unless otherwise noted[158,159]. Radiocarbon dates not accompanied by a citation were generated as part of the present study. A short paragraph describes the archaeological site and the circumstances under which the sample was recovered, followed by a description of the particular *Plasmodium*-positive sample. Sample IDs used in this study are printed in bold, with associated archaeological IDs used in publications and site reports given in parentheses. Altitudes are given in meters above sea level (masl).

Archaeological Background, Table of Contents:

1. Archaeological Site and Sample Information for *P. falciparum* Endemic Sites
    1.1 Bourges, Monin
    1.2 Castelsardo
    1.3 Chokhopani
    1.4 Corona Moltana/Zarau
    1.5 Galleon Beach
    1.6 Göttlesbrunn
    1.7 Heping Dao B
    1.8 Lippstadt
    1.9 Paris Réaumur-Sébastopol
    1.10 Torčec, Crkvišće
2. Archaeological Site and Sample Information for *P. vivax* Endemic Sites
    2.1 Cueva de las Lechuzas
    2.2 Gâmbaş – Site 3
    2.3 Gars/Thunau
    2.4 Gózquez
    2.5 Gundorovka
    2.6 Laguna de los Cóndores
    2.7 Leubingen
    2.8 Lungi Tepa
    2.9 Mikulovice
    2.10 S'Orcu 'e Tueri (Perdasdefogu)
    2.11 Tarquinia
    2.12 Thebes
    2.13 Voivodeni
    2.14 Volosovo
3. Archaeological Site and Sample Information for *P. malariae* Endemic Sites
    3.1 Sosnovaya griva 3
4. Archaeological Site and Sample Information for Endemic Sites with Multiple *Plasmodium* Species
    4.1 Mechelen, St. Rombout's cemetery

1. Archaeological Site and Sample Information for *P. falciparum* Endemic Sites

1.1.1 Bourges, Monin

Country: France
Region: Centre-Val de Loire
Coordinates: 47.075912°, 2.402421°
Sample Date (MOB025): 1100-1300 CE (according to radiocarbon analysis of several burials). No direct radiocarbon dates have been produced for the sample MOB025 itself.
Radiocarbon Date: -
Excavation/Sample Provenance: Excavated between April 2018 and October 2019 under the auspices of Archaeological Survey Service of Bourges Plus.

Site Description:
Situated in downtown Bourges, the site served as a burial ground for the medieval city's inhabitants from the 10th century through the end of the 16th century CE. From the 12th century it is known as the Grand Cimetière, a space reserved for the burial of the poorest populations or those wishing to be buried among the most humble. Excavations in 2018 and 2019 uncovered 333 medieval burials, including 92 multiple burials; the unexpectedly large number of multiple burials is consistent with several elevated mortality events, if the burials are simultaneous primary deposits[160]. Containing between two and ten individuals for a total of 233 deceased, these multiple burials derive from at least three separate phases of the cemetery's long use period. It was hypothesized that the graves might contain victims of the multiple historically-attested outbreaks of plague that struck Bourges following the onset of the Black Death from 1347 CE; however, radiocarbon dating places the burials in the 12th-13th century CE, suggesting a different causative agent(s) for the elevated mortality observed at the site of Bourges. The marshy environment surrounding Bourges presents a suitable environment for the proliferation of *Anopheles* spp., consistent with our finding of malaria at the site. The final operation report for the excavation is being drafted and will be available during the first quarter of 2024.

- **MOB025 (SP7-392 US 7937)-** We recovered *P. falciparum* mitochondrial and nuclear DNA from the lower right 2nd molar of an adult female individual who probably died between the ages of 18 and 25 considering her bone maturation. In addition to non-lethal dental lesions, this individual presents signs of metabolic stress with traces of *cribra orbitalia*, especially pronounced on the left orbit. A fetus of 4 to 5 months of age (mean fetal age: 18 to 19 weeks amenorrhea) was recovered from the pelvic cavity of individual MOB025. The arrangement of the bones of this fetus showed no evidence of a perimortem delivery.

1.1.2 Castelsardo
Country: Italy
Region: Sassari, Sardinia
Coordinates: 40.9130204°, 8.6971407°
Sample Date (CSR001): c. 1600 CE (archaeological context, stratigraphic observations)
Radiocarbon Date: -
Excavation/Sample Provenance: Excavations in 2011 associated with cathedral restoration.

Site Description:
The Cathedral of Sant'Antonio Abate is in the small town of Castelsardo, situated on the northern coast of Sardinia in the province of Sassari, Italy. In 2011, excavations associated with cathedral restoration unearthed a crypt containing 18 mummified or partially-mummified individuals, including both children and adults[161]. Votive medals and pottery fragments found in the different ground levels of the crypt were used to determine the age of each level, and it appeared that the bodies were interred between 1600 and 1830 CE. Individual CSR001 was interred in a layer associated with the earlier use phase. The unsystematic layering of large numbers of individuals in the same burial suggests that the grave may be associated with a mass mortality event, such as the historically-attested local plague outbreak of 1653. The detection of

P. falciparum at Castelsardo is compatible with the endemic presence of the etiological agent of malaria in Sardinia till its elimination c. 1950.

- **CSR001 (37)**- We generated a *P. falciparum* mitochondrial genome from a premolar from individual CSR001.

1.1.3 Chokhopani

Country: Nepal
Region: Annapurna Conservation Area, Upper Mustang
Coordinates: 28.7833333°, 83.7166667°
Sample Date (CHO001): 804-765 BCE, according to radiocarbon dating of a birch bark vessel from one of the neighboring burials (2575 ± 19 BP, 804-765 calBCE, 2-sigma, Hd 15597)[39]. No direct radiocarbon dates have been produced for the sample CHO001 itself.
Radiocarbon Date: -
Excavation/Sample Provenance: The first rescue and recovery excavations were conducted in 1984 by the Department of Archaeology, Government of Nepal. Subsequent rescue excavations were conducted between March and May 1992 by teams from the Institute of Prehistory at the University of Cologne and the HMG Department of Archaeology of Nepal.
Human Genetic Analysis: Jeong *et al.* 2016; Liu *et al.* 2022

Site Description:
Situated in one of the high transverse valleys of the Himalayas at 2800 masl, Chokhopani lies near the modern village of Marpha on the east side of the Kali Gandaki river in lower Mustang, Gandaki Province, Nepal[50]. Chokhopani consists of a series of shaft tombs overlooking the river valley, which have been collapsed over time by geological processes that have altered the cliff's face[48]. A shaft cut during the construction of a micro-hydro power line destroyed the remaining cave system, and subsequent rescue excavations recovered the remains of at least 21 individuals from three burial chambers. Radiocarbon analysis of an accompanying birch bark vessel dated the burials to the early first millennium BCE (2575 ± 19 BP, 804-765 calBCE, 2-sigma, Hd 15597), while associated copper artifacts support interactions with lowland South Asian populations[39,49].

- **CHO001 (CNE1)**- In this study, we recover genome-wide *P. falciparum* mitochondrial and nuclear data from a tooth of a genetically male adult individual, CHO001 (CNE1). Previous studies document a close genetic relationship between early inhabitants of the Mustang region, including CHO001, and modern high altitude East Asian populations, such as Tibetans and Sherpa[50,162]. Consistent with this ancestry, CHO001 possesses a derived allele of the *EGLN1* gene associated with adaptation to high-altitude environments, while the well-known high altitude-adaptive *EPAS1* haplotype is absent in individuals from Chokhopani and contemporaneous sites[50,162]. Interestingly, in contrast to individuals from other high-altitude prehistoric sites in Mustang, only individuals from Chokhopani show signatures of admixture with mid- to low-altitude South Asian populations[50].

1.1.4 Corona Moltana/Zarau
Country: Italy
Region: Sassari, Sardinia
Coordinates: 40.5192°, 8.789147°
Sample Date (COR001): 885-992 CE (radiocarbon dating)
Radiocarbon Date (COR001, temporal/petrous bone, MAMS-38278): 1124 ± 23 BP, 885-992 calCE (AMS, 2-sigma)[131].
Excavation/Sample Provenance: Excavations by G. Meloni in 2000.
Human Genetic Analysis: Marcus *et al.* 2020

Site Description:
Located near the northwestern Sardinian town of Bonnanaro, the site of Corona Moltana/Zarau consists of a large series of rock-cut tombs, caves (*anfratti*), and rock shelters[131]. While an 1889 excavation of one tomb at the site recovered material dating to the Early Bronze Age, local oral histories suggested that the site's occupation continued into recent times; the place was thought to have served as a residence and final resting place for a local family suffering from an unidentified illness. In 2000, several tombs were excavated by G. Meloni, including our sample COR001; while the skeletal remains lacked accompanying artifacts, radiocarbon analysis placed the burials in the Early Medieval Period[131].

- **COR001 (ind. 1)**- We recovered *P. falciparum* mitochondrial data from a genetically female individual buried in Anfratto 1 at Corona Moltana/Zarau.

1.1.5 Galleon Beach
Country: Antigua and Barbuda
Region: Island of Antigua
Coordinates: 17.0239000°, -61.7745000°
Sample Date (GBA004): 18th century CE (archaeological context)[163]
Radiocarbon Date: -
Excavation/Sample Provenance: Excavations by A. R. Murphy, M. Brown, T. Varney, C. Look, 2012-2016.

Site Description:
Galleon Beach is located on the southern shore of the island of Antigua, Antigua and Barbuda. Situated at the mouth of English Harbour, Galleon Beach lies in close proximity to the former British Royal Navy Dockyard, which served as a primary site for British Naval activities in the Lesser Antilles between 1725 and 1815, and as a secondary repair and coaling station until 1890[163]. During this period, English Harbour gained notoriety for an extraordinary prevalence of infectious diseases, earning the moniker "The Grave of the Englishman". In 2007, the previously unmarked burial site was discovered after a tropical cyclone blew out part of the beach. Between 2012 and 2016 the site was subject to controlled archaeological recovery. Context suggests burial during the naval occupation of English Harbour; however, no diagnostic materials or archival information to date has been able to narrow down the temporal span. The current working assumption is that these were opportunistic burials for sailors. Previous excavations on a nearby

hill revealed portions of a naval hospital and associated cemetery, which was used extensively from 1793-1822 CE[163]. Research is ongoing[164].

- **GBA004 (ANT-HIS-19)-** Here we report the recovery of a mitochondrial *P. falciparum* genome from a tooth of an adult individual excavated in 2015 (unit 3, burial 2). Anthropological assessment suggests that the individual is male.

1.1.6 Göttlesbrunn

Country: Austria
Region: Lower Austria, Bruck an der Leitha
Coordinates: 48.0518075°, 16.7307301°
Sample Date (GOE016): Early La Tène period according to overall archaeological context of the cemetery (La Tène B1 to the turn of La Tène B2/C, 350-250 BCE); precise archaeological dating of this particular individual is hampered by the lack of chronologically sensitive artifacts in the grave. Early to Middle La Tène period (La Tène B to La Tène C) according to radiocarbon dating.
Radiocarbon Date (GOE016, femur fragment, VERA-7372): 2205 ± 29 BP, 371-176 calBCE (AMS, 2-sigma)
Excavation/Sample Provenance*:* Excavations by J. M. Czubak in Aug.-Oct. 2016, May-July 2018, and Jan.-Mar. 2020.

Site Description:
Situated on the fringes of the temperate Pannonian Basin near the town of Göttlesbrunn-Arbesthal in Lower Austria, the eponymous site consists of a partially excavated cemetery dating to the Late Iron Age La Tène culture[165]. Excavations led by J. M. Czubak in 2016, 2018, and 2020 uncovered 24 graves (2 double, 1 triple, 1 empty) with the remains of 27 individuals (26 inhumations, 1 cremation), which were distributed in two clusters within a 200 m by 30 m strip bordering today's A4 motorway. Despite evidence of extensive looting, likely also dating to the La Tène period, the burials contained diverse grave goods including pottery, gravestones/stelae, jewelry, and fragments of sword scabbards and weapons[165]. Consistent with the site's location near the crossroads of two major Late Iron Age trade routes, grave goods recovered from nearby La Tène cemeteries support the area's importance as a center of both regional and long-distance connectivity[40].

- **GOE016 (Grave 13, feature 091, section/VF 08, year 2018)-** We recovered *P. falciparum* genome-wide nuclear and mitochondrial data from the molar of a 25-45-year-old male buried with sword scabbard and lance tip fragments.

1.1.7 Heping Dao B

Country: Taiwan (R.O.C.)
Region: Keelung, Zhongzheng District
Coordinates: 25.155812°, 121.764807°

Sample Date (HPD007): 1626-1668 CE (historical sources, archaeological context and radiocarbon dating). No direct radiocarbon dates have been produced for the sample HPD007 itself.
Radiocarbon Date: -
Excavation/Sample Provenance: Excavated 2014 Further excavation campaigns between 2019 and 2023 increased the number of burials revealed to a total of 23.

Site Description:
The island of Heping Dao lies just off the northern coast of Taiwan opposite Keelung. Between 2011-2016, a joint Spanish-Taiwanese archaeological team excavated a total area of approximately 238 m^2 in a parking lot area referred to as Heping Dao B[166]. Excavation revealed a complex archaeological record at Heping Dao B, with evidence of continuous occupation dating from the Neolithic to the present day[166]. Historical documents link Heping Dao with the Spanish colony of San Salvador de Kelang or Quelang, which lasted from 1626 CE until its decline in 1642 CE. Then the Dutch East India Company (*Vereenigde Oostindische Compagnie*) took over the colony, which endured until 1668 CE, when Europeans were expelled from Taiwan by Ming loyalists. The remains analyzed here were recovered from a cemetery associated with the European colonial Convent of All Saints and its church, first Dominican, under Spanish rule, and later Protestant, under Dutch rule. The burials have been dated through their very clear archaeological context. They belong to both the Spanish and Dutch periods[166,167].

- **HPD007 (HPD - B 2014 T3P2 UE26 L I2)-** We recovered *P. falciparum* mitochondrial and nuclear genome-wide data from a permanent incisor from an adult male individual.

1.1.8 Lippstadt
Country: Germany
Region: North Rhine-Westphalia
Coordinates: 51.67131°, 8.676931°
Sample Date (LIP011): 1757-1758 CE (historical sources, archaeological context)
Radiocarbon Date: -
Excavation/Sample Provenance: Excavated in June 2015 by Archaeonet GBR, Bonn under supervision of the responsible monument authority LWL-Archäologie für Westfalen.

Site Description:
During construction works at Lippstadt in North Rhine-Westphalia 60 km east of Dortmund, 49 graves arranged in five rows were discovered. The graves contained a minimum number of 68 individuals. Overall the graves consisted of 39 single burials, seven double burials, and two triple burials all laid out in wooden coffins. Especially in the double and triple burials different stages of anatomical dislocation were present. According to finds of pottery and historical records the burials are probably connected with the military conflicts around the town between the French and Prussian armies in 1757/1758 during the Seven Years War.

- **LIP011 (Bef. 18)-** We recovered a *P. falciparum* mitochondrial genome from a tooth from an adult individual discovered in *Befund* 18, probably a French or Prussian soldier

fighting the Seven Years' War. Anthropological assessment indicates that the individual is likely male.

1.1.9 Paris Réaumur-Sébastopol

Country: France
Region: Île-de-France
Coordinates: 48.866226°, 2.3525439°
Sample Date (PRS010): c. 1280-1395 CE (radiocarbon dating, stratigraphy). No direct radiocarbon dates have been produced for the sample PRS010 itself.
Radiocarbon Date: -
Excavation/Sample Provenance: Excavated in 2015 under the auspices of the Institut National de Recherches Archéologiques Préventives (Inrap).

Site Description:
Excavations in the basement of the 1910 Félix Potin building on Paris's Boulevard de Sébastopol uncovered the remains of 314 individuals from eight multiple burials[168]. The excavated individuals belong to a larger cemetery associated with the medieval/early modern hospital of La Trinité. Founded in 1202 CE as the Hôpital de la Croix de la Reine and located adjacent to the city gates, La Trinité originally served as a welcoming point for pilgrims and travelers entering the city. Over time, the institution's primary function became caring for the sick, although it also housed orphans, disabled veterans, and other disadvantaged populations[168].

The hospital's cemetery served the population of Paris from 1353 CE until its closure at the end of the 17th century CE. The eight multiple burials excavated thus far vary in size and belong to three chronological groups, spanning the late 13th through the mid 17th century CE. The oldest and largest of the multiple burials, burial 5 contains the remains of at least 218 simultaneously deposited individuals. It exhibits characteristics associated with a mass mortality event, and is temporally associated with the outbreak of the Black Death[168]. Smaller, multiple burials from later phases exhibit less evidence of organization and careful deposition, suggesting that the interred individuals may have occupied a lower social class.

- **PRS010 (Ind. 144)-** We recovered genome-wide nuclear and mitochondrial *P. falciparum* DNA from a molar from this individual interred in burial 5.

1.1.10 Torčec, Crkvišće

Country: Croatia
Region: Koprivnica-Križevci, Drnje
Coordinates: 46.22391°, 16.877164°
Sample Date (TOR008): 1250-1750 CE (archaeological context)
Radiocarbon Date: -
Excavation/Sample Provenance: Excavations from 2002 to 2015 under the auspices of the Archaeology Institute in Zagreb.

Site Description:
The archaeological site Torčec, Crkvišće is a multi-layered historical cemetery located on a slight terrace adjacent to an old meander of the Drava river in northern Croatia, near the modern town of Koprivnica. Inhumations in the cemetery date from the mid-13th to the mid-18th century CE[169]. A total of 355 individuals have been recovered from the cemetery, and osteological analyses performed at the Croatian Academy of Sciences and Arts document a high incidence of subadult mortality, particularly in children under five years of age[170], as well as the presence of leprosy, tuberculosis, and perimortem injuries.

- **TOR008 (250)-** We recovered *P. falciparum* nuclear and mitochondrial data from a female individual aged 25-30 years at the time of death. Ancient *Plasmodium* DNA was extracted from a calcified hydatid cyst, which was originally thought to derive from infection by a dog tapeworm (*Echinococcus granulosus*). This individual also exhibits bilateral, moderate, active periostitis on the tibiae and linear enamel hypoplastic defects on the maxillary central incisors and canines.

2. Archaeological Site and Sample Information for *P. vivax* Endemic Sites

1.2.1 Cueva de las Lechuzas
Country: Spain
Region: País Valencià, Alicante
Coordinates: 38.6303°, -0.863°
Sample Date (CLL005): c. 3300-2300 BCE (archaeological context)[42]
Radiocarbon Date: -
Excavation/Sample Provenance: Excavations by J. M. Soler.
Human Genetic Analysis: Villalba-Mouco *et al.* 2021

Site Description:
Located in the Villena basin in Southeastern Iberia, the cave site of Cueva de las Lechuzas is situated on the eastern slope of the Cabezo de las Cuevas[42]. Originally accessed by a 2 m wide opening, portions of the single main chamber were destroyed during quarrying activity, leading to the discovery of human remains. Subsequent excavations of the cave by J. M. Soler recovered skeletal material from at least 18 individuals, as well as a rich assemblage of associated grave goods. Material finds and the collective burial context date the assemblage to the Southeastern Iberia Late Neolithic/Chalcolithic, c. 3300-2300 BCE[42].

- **CLL005 (Lech 7)**- We recovered *P. vivax* genome-wide nuclear DNA from an adult genetically male individual. CLL005 was also infected with Hepatitis B Virus (HBV), as previously published[171].

1.2.2 Gâmbaş – Site 3
Country: Romania
Region: Alba County, Transylvania
Coordinates: 46.345816°, 23.722172°

Sample Date (GAS004): Middle Bronze Age (archaeological context), 1870-1630 BCE (radiocarbon date)
Radiocarbon Date (GAS004, temporal/petrous bone, MAMS-51998): 3428 ± 21 BP, 1872-1631 calBCE (AMS, 2-sigma)
Excavation/Sample Provenance: Excavated under the auspices of the National Museum of Unification in Alba Iulia between November 2014 and April 2015 throughout preventive archaeological investigations conducted on the route of a future motorway.

Site Description:
The archaeological site at Gâmbaş was discovered and delimited by archaeologists from the National Museum of Unification in Alba Iulia through an intrusive diagnosis conducted on the route of a future motorway. Subsequent preventive archaeological investigations started in November 2014 and continued, for the second part of the site, at the end of January 2015. By the end of the excavation campaign in April 2015 the site was fully investigated.
The site is situated on the left bank of the Mureş river, more precisely on the portion of the first river terrace which is at the border of the Gâmbaş locality. The area had been settled across several prehistoric and historical ages: the Middle Bronze Age (Wietenberg Culture), Early Iron Age (G.va Culture), Late Iron Age, represented by Dacian features (of the 1st century BCE - 1st century CE), Post-Roman Period (4th-6th century CE), and Migration Period (7th-8th century CE). 217 features were identified in the excavated area at Gâmbaş, dated to the Bronze Age and assigned to the Wietenberg Culture, consisting of storage pits turned into refuse pits, various pits with special characteristics, dwellings, and inhumation burials. Four Bronze Age burials were observed in different areas throughout the settlement[172].

- **GAS004 (Ft 347)**- We recovered genome-wide *P. vivax* nuclear DNA from an adult genetically male individual from Gâmbaş. The archaeological association with the Middle Bronze Age Wietenberg culture was confirmed by direct radiocarbon dating of the skeleton. GAS004 was recovered from the east-central part of the settlement. The deceased lay crouched, with the head towards SE. Two ceramic fragments were found in the grave. It was cut by another, from a more recent period. From the cranium, only four permanent teeth remain, and three teeth were lost before death (the 2nd and 1st left mandibular molars and the 1st right mandibular molar). Additionally, three teeth indicated signs of decay (the 3rd mandibular molar, the maxillary canine and the 2nd maxillary molar, all from the right side), and two abscesses appear at the following molars: 1st left maxillary, 2nd right mandibular. From the post-cranium, of the 13 joints that are present, six are affected by weak osteoarthritis: the right acromioclavicular joint, the right distal humerus, the right proximal radius, the right distal ulna, the thoracic segment of the spine, and the left glenoid cavity. Healed porotic hyperostosis is present on the cranial bone, but without being associated with cribra orbitalia. On both humeri, the insertion of the pectoralis major is well accentuated, with several bony exostoses[172].

1.2.3 Gars/Thunau
Country: Austria
Region: Lower Austria
Coordinates: 48.6009°, 15.6623°

Sample Date (GAT004): 800-1000 CE (archaeological context)
Radiocarbon Date: -
Excavation/Sample Provenance: Throughout archeological excavations between 1965 and 2021, the skeletal remains of a total of 456 individuals were discovered, 301 at the hilltop settlement, and 155 at the riverine site of Gars/Thunau. They were handed over to M. Teschler-Nicola (Department of Anthropology, Natural History Museum, Vienna), inventoried under her leadership, and analyzed within the framework of two research projects funded by the Austrian Fonds zur Förderung der wissenschaftlichen Forschung (FWF) with different focal points: Teschler-Nicola, M., "Allochthonie und Autochthonie in der Kamptalregion" (FWF P09491-HIS) und Prohaska, T. (FWF-Start-Project P267N195). Furthermore, several academic theses have been completed under M.T.-N.'s supervision and/or in collaboration with colleagues from the University of Agricultural Sciences (BOKU) and the Vienna Environmental Research Accelerator-Laboratory (VERA-LAB) focussing on palaeo-epidemiological aspects, malnutrition, mobility, and migration[173–185].

Site Description:
First investigations in Thunau am Kamp, Lower Austria, a village in the border area between Wald- and Weinviertel, approx. 25 km north of the Danube, Katastralgemeinde of the market town of Gars am Kamp (Politischer Bezirk Horn, Lower Austria) date back to the beginning of the 19th century. From the 1870s and particularly during railroad construction in the 1880s the site was increasingly the focus of archaeological research. Systematic and regular excavations at the so-called "*Schanzburg*", a hilltop settlement, were initiated by Herwig Friesinger only in 1965 and continued by his collaborator Anton Kern and (since 1993) by Erik Szameit until 2003[186–188]. From 1975 excavations and rescue-excavations (1975, 1983, 1985-1987) under the leadership of Herwig Friesinger, Erik Szameit, Falko Daim and Martin Obenaus also took place in the valley area of Gars/Thunau. Here, from 2004-2023, systematic investigations were intensified by Erik Szameit and Martin Obenaus, who uncovered many previously unknown structural details, such as the extent and function of this locality and interpreted it as a *suburbium*[189–191]. Both areas of this central site date to the late phase of the Early Middle Ages (9th to 10th centuries CE)[192]. The fortified hilltop settlement also included a fortified manor house, a church, and a cemetery of mostly densely and regularly arranged inhumation burials in supine position[193–197]. The valley settlement, on the other hand, was unfortified; the cemetery lies north of the settlement center and probably contained almost five times as many burials as the hilltop settlement's cemetery. Only a part of the cemetery in the valley could be archaeologically investigated so far, but further burials in the settlement and irregular deposits of human bodies and/or body parts either in houses or settlement pits have been discovered. Grave goods, other cultural objects, and features at the riverine area indicate large-scale craft production (among other things, for example, more than 500 loom weights came to light) and processing of agricultural goods (storage pits, rotary quern stones, and baking ovens). Subsequently, the archaeologists interpreted this area as a production-based *suburbium* with affiliated "industrial craft zone" closer to the river, obviously less densely populated than the hilltop locality and most likely inhabited by craftsmen, farmers, and their families[198]. Under these circumstances, it seems plausible that the people of the valley settlement were responsible for supplying the manor house or its users as well as the whole fortified hilltop settlement. Although both facilities were used at about the same time - late phase of the Early Middle Ages (9th-10th centuries CE) - their purposes diverged, and there is bioanthropological evidence that these circumstances also influenced

lifestyles, such as nutrition and workload, to varying degrees. In comparison to the hilltop settlement, which ended around the middle of the 10th century, the riverine settlement seemed to be still in use up to the transition from 10th to 11th centuries.

As mentioned above, the site Gars/Thunau includes both a fortified hilltop settlement and necropolis and an associated lowland riverine population center. Both portions of the site are contemporaneous and date to the early medieval period (9th -10th century CE). The site's central character in the 9th and 10th centuries CE is due to the topographical situation (N-S axis) through the Kamptal valley and the location in a geopolitically significant border region between political powers: in a contact zone about halfway between the realm of the Eastern Franks (i.e. Fränkisch-Bayrisches Ostland) along and south of the Danube and the Moravian principality with its centers in today's southern Moravia[199].

Weapon finds and pieces of riding equipment, but also the conspicuous frequency of pathomorphological alterations and traumatic changes observed in the human skeletal remains as well as the results of isotope studies seem to reflect the site's hypothesized military function and also its function in supra-regional traffic and exchange networks. It is assumed that the hilltop site served as a residence for the social and military elite, whereas the riverine section, the so-called *suburbium* or its affiliated industrial zone closer to the river, was inhabited by a less prosperous population. Studies included – among others – strontium isotope analyses which emerged as crucial due to the high number of non-local individuals (88% at the hilltop, 90 % at the riverine settlement, respectively), supporting the hypothesis of a role in a military and supra-regional exchange network context[178,183,185]. We have some arguments based on the archaeological record that this location may have acted as a trading center as well. The findings and the bioanthropological data, in particular the high mortality rate of immatures in both areas (up to 60%), the incidence of tuberculosis and other communicable diseases, known as most likely associated with crowded urban environments, seem to corroborate the locations' function(s). Stress episodes indicated by high frequencies of malnutrition symptoms and unfavorable living conditions could have affected the demographic, functional, and social conditions at this site: among the many pathological alterations which we cannot exhaustively list here, there are several cases of (active and remodeled) Pott's disease and joint tuberculosis linked with *Mycobacterium tuberculosis* infection; a high portion of possible (unspecific) tuberculosis symptoms, e.g., new bone formations at the inner surface of the ribs and at the cranial base; symptoms of bacterial infections most likely caused by *Brucella* and *Actinomyces*[200]; lethal and healed injuries, including several cases of decapitation and other mutilations/cutting of body parts induced probably by corporal punishment (*Leibesstrafen*); a high level of workload (biomechanical stress) implied by bone formation at entheseal sites; sex-specific degenerative joint and vertebral lesions, e.g., spondylolysis only in females pointing to their heavy workload[182]; a high number of malnutrition symptoms and a possible case of intoxication (probably by consumption of Ergot alkaloids)[201]. A few selected immatures exhibiting endocranial lesions and one adult characterized by a progressed joint tuberculosis were successfully investigated for the molecular genetic evidence of the causative organism by the Natural History Museum's aDNA experts under the leadership of Elisabeth Haring[185].

The early medieval population of Gars/Thunau located in the frontier area between the realm of the Eastern Franks, the Moravian Principality and Bohemia must not be considered as socially homogeneous, since in the early Middle Ages there was not yet a clear separation between the militarily active and the civilian population. This intermediary situation between different spheres of political power associated with the specific function of the central site is

consistent with a period of intensified contacts with other cultures in a wider geographical area of differing characters.

The individual under discussion in the present study, GAT004/ FN 134 was recovered as a so-called deviant or anomalous burial from a storage pit at the riverine area and was identified as a c. 50-70-year-old female. During the bioanthropological analysis an object, identified by macroscopic inspection as calcified tissue, was taken to gain information about its anatomical location and pathophysiological causes. Moreover, it was aimed to test the potential of aDNA analysis in such a complex matter. Such a formation is often associated with lifestyle, and in many cases nutrition and fluid intake play a decisive role. Some body stones also develop as a product of inborn errors of metabolism or are related to chronic diseases or infectious diseases[202].

- **GAT004 (134/GT 2020)-** We recovered *P. vivax* genome-wide nuclear data from a portion of calcified tissue from an adult female individual with the find no. (FN) 134 (SE 333, *Schnitt* 19/burial 1). She was recovered from a settlement pit at the riverine site during the excavation campaign in 2020. Based on the finding depth it was denoted as "burial 1"; below, a second individual was uncovered. The cranium of the female ("1") is nearly complete, the post-cranial portions are in part preserved, and the cortical surface of all bony elements show only mild erosive alterations.

  All morphological sex specific features are characteristically female. Age-at-death estimation was based on the complete ossification of the cranial sutures, the premortem loss of 8 teeth, and the fact that all bones are extremely osteoporotic (cortical thinning; loosened cancellous bone structure, particularly concerning the sternum), thus estimated to be c. 50-70 years.

  Pathomorphological alterations and other changes include, e.g., slightly developed *hyperostosis frontalis interna*; porotic palate (*stomatitis*), porotic and reduced alveolar rim of the mandibular and maxillary tooth compartments; signs of inflammation at the right and left sinus maxillaris (*sinusitis*); caries lesions at 41, 45, 46, granuloma at 14 and dental calculus appositions are on all remaining teeth visible; degenerative changes were found at the caput mandibulae and the right first metacarpal (*osteochondritis dissecans*); slight spondylosis and spondylarthrosis and a compression fracture in form of a deep cover plate impression at the second lumbar vertebra is observable; striae (features of remodeled periostitis) are visible at all long bones (caused by vitamin deficiency in early childhood); of particular interest are two small (2 cm x 1 cm; 1 cm x 0,5 cm) whitish to gray calcified objects mentioned above, with an irregularly shaped surface representing crystalline deposits of minerals and salts. Their features suggest a genesis in the renal pelvico-caliceal system (nephroliths, kidney, or urinary stones).

1.2.4 Gózquez

Country: Spain
Region: Madrid
Coordinates: 40.233°, -3.6°
Sample Date (GOZ006): 544-645 CE (radiocarbon dating). Two bone samples from one (unspecified) individual from the same burial were previously radiocarbon dated (CSIC-1560: 1503±37 BP; BETA-135022: 1390±60 BP). New radiocarbon analyses of two separate individuals interred with GOZ006 date the burial to 544-645 calCE (MAMS-48802: 1462±22

BP, 571-645 calCE, 2-sigma; MAMS-48803: 1500±23 BP, 544-636 calCE, 2-sigma). No direct radiocarbon dates have been produced for the sample GOZ006 itself.
Radiocarbon Date: -
Excavation/Sample Provenance: Rescue excavations under the direction of G. A. Vigil-Escalera were carried out between 1997 and 2000, prior to the construction of a leisure park at the site. The final report of the excavations by Vigil-Escalera remains unpublished (title: *Memoria de las excavaciones arqueológicas en el yacimiento de época visigoda de Gózquez de Arriba).* However, a comprehensive summary of the results appeared in 2013[203].

Site Description:
The site of Gózquez is located 20 km to the southeast of the city of Madrid and 56 km to the northeast of Toledo, former capital of the Visigothic kingdom, on the southern bank of a stream that flows into the Jarama river. It is one of the most extensively excavated rural settlements of this period in the Iberian Peninsula (about three hectares). The village is divided into two neighborhoods with the cemetery of the community placed between them. It contains ten to twelve households with a strict allocation of their respective plots, from the initial arrangement until its abandonment. A highly detailed sequence of occupation was achieved through pottery analysis and the radiocarbon dating of some key contexts. Apart from the around 400 individuals buried in the necropolis, another ten (at least) were buried without recognizable funerary treatment in various pits formerly used as silos in the house yards[204]. The study of faunal and botanical records from Gózquez has prompted a challenging picture of the productive strategies of early medieval peasant communities[205].

- **GOZ006 (silo 6640 Ind. 3)-** We recovered *P. vivax* genome-wide nuclear data from individual 3, an individual interred in a multiple burial inside an abandoned grain silo (silo 6640). An unpublished anthropological report by E. C. Sampedro entitled *Análisis de los restos antropológicos procedentes del yacimiento 050 de San Martín de la Vega (Madrid)* indicates that the human remains of silo 6644 belong to five individuals, all of them complete: (1) young adult, male, age 30-35, with approximate height between 1,60 and 1,65 m. Marked dental attrition and some bone degeneration, especially in intermediate cervical, lumbar, and lower thoracic vertebrae. Degenerative changes affecting the upper extremities, especially the right humerus and ulna, were also observed. Caries were identified in two teeth, and dental calculus in almost all the dentition. (2) Young female with an estimated age 15-17. (3) Child individual, 6 years ± 24 months. (4) Young female, 19-20 years, with some dental wear on the first molars, quite high for her estimated age. Caries in the intermediate zone between the first and second molars. Estimated height between 1,55-1,60 m. Pathology in the left tibia that does not seem to be a consequence of a fracture (x-ray would be necessary to determine the type of injury). (5) Child individual, estimated age 11 years ± 30 months, with severe cribra orbitalia in both eye sockets. The report states in page 5 that "*Desconocemos la causa de la muerte de estos individuos, ya que no se ha encontrado ninguna evidencia en los restos óseos, pero las características de los enterramientos sugieren alguna de las grandes epidemias de peste que nos consta que existieron en esos días*" ("We do not know the cause of death of these individuals, since no evidence has been found in the skeletal remains, but the characteristics of the burials suggest some of the great plague epidemics that we know existed in those days").

1.2.5 Gundorovka

Country: Russian Federation
Region: Samara Oblast, Krasnoyarskiy Rayon
Coordinates: 53.718713°, 50.677327°
Sample Date (GUD004): ca. 3000 BCE/turn of the 4th/3rd millennium BCE (archaeological context)
Radiocarbon Date: -
Excavation/Sample Provenance: Excavated between 1985 and 1989 by I. B. Vasilyev, A. E. Mamonov, and N. V. Ovchinnikova.

Site Description:
The settlement and burial site of Gundorovka is situated in the forest-steppe on the Sok River in the Krasnoyarsky District of Samara Oblast, Russia. A total area of 1256 m^2 was excavated between 1985 and 1989, exposing a stratigraphy dating from the Neolithic-Eneolithic period though the Middle-Late Bronze and Early Iron Ages. The majority of the settlement remains are connected with the Eneolithic[43]. Excavations unearthed a total of twelve burials, with numbers 3-11 contextually associated with the Neo-Eneolithic periods, and 1-2 with the Middle Bronze Age. Radiocarbon analysis of skeletal remains from four burials (4, 9-11) strengthens their connection with the Eneolithic period, but also indicates that they may be older than the individual (GUD004 - burial 5) analyzed for the present study.

- **GUD004 (burial 5)-** We report nuclear genome-wide *P. vivax* data from the Eneolithic adult male individual from Gundorovka.

1.2.6 Laguna de los Cóndores

Country: Peru
Region: San Martín
Coordinates: -6.85135°, -77.696297°
Sample Date (LDC020): 1436-1617 (radiocarbon dating); while one of the radiocarbon dates places this individual in the Late Intermediate Period, a second tooth from this individual produced a date spanning the Spanish contact period (cf. below).
Radiocarbon Date 1 (LDC020, tooth, MAMS-35124): 429 ± 16 BP, 1437-1473 calCE (2-sigma)
Radiocarbon Date 2 (LDC020, tooth, MAMS-58729): 405 ± 21 BP, 1441-1617 calCE (2-sigma)
Excavation/Sample Provenance: Rescue excavations in 1997 to prevent further looting.
Human Genetic Analysis: Analysis of LDC020 presented in this study.

Site Description:
Situated at an altitude of 2800 masl in the cloud forests blanketing the eastern slopes of the northern Peruvian Andes, Laguna de los Cóndores consists of a series of funerary structures, known as *chullpas*, built on a cliff ledge 325 feet (c. 100 m) above the lake[52,206]. Across the lake, the *chullpas* overlook the remains of the large village of Llaqtacocha, which contained approximately 200 dwellings; both the burial houses and the village are associated with the Chachapoya culture, a loose confederation of peoples that inhabited highland regions of the northeastern Peruvian Andes between c. 800 and 1470 CE, when they were conquered by the Inka[206,207]. Discovered by ranch hands in 1996, extensive looting impacted 90% of the site before

rescue excavations began the following year[206]. Nevertheless, several hundred mummy bundles, skeletonized remains, and countless grave goods recovered from the site display an exceptional degree of organic preservation. Assessment of material culture and radiocarbon dating of human remains and organic artifacts indicate that the *chullpas'* use period began prior to the Incan conquest (Late Intermediate Period, 1000-1475 CE), continuing through the Incan occupation (Late Horizon, 1475-1532 CE) and into the Spanish colonial era[206,208]. Indeed, recovery of colonial-era artifacts including a Christian crucifix supports the site's continued use post-contact. However, the short duration of the Late Horizon and unfavorable form of the calibration curve complicate attempts to establish chronology through radiocarbon dating[208]; similarly, establishment of a contextual archaeological chronology has been challenging due to the extensive looting that took place prior to the onset of archaeological excavations.

- **LDC020 (CHA99)-** We recovered genome-wide *P. vivax* mitochondrial and nuclear data from two teeth from one genetically male individual from Laguna de los Cóndores. The skeletonized remains of this individual were commingled with others as a result of looting.

1.2.7 Leubingen
Country: Germany
Region: Thuringia
Coordinates: 51.206201°, 11.183455°
Sample Date (LEU028): 3637-3528 BCE (radiocarbon dating)
Radiocarbon Date (LEU028, temporal/petrous bone, MAMS-43524): 4789 ± 22 BP, 3637-3528 calBCE (2-sigma)
Excavation/Sample Provenance: Excavations from 2009 to 2010 and 2016 to 2020 by the Thuringian State Office of Heritage Management and Archeology.

Site Description:
Leubingen is located in the Thuringian basin, a highly fertile region in the present-day German state of Thuringia. The earliest archaeological activity at Leubingen was the excavation of the well-known early Bronze Age "prince's" tumulus. Before the construction of the A 71 motorway and a service station, archaeological excavations were carried out on a contiguous area of 22.5 ha in 2009 and 2010. During the excavations, 6,141 archaeological features were documented[209]. Radiocarbon analyses and archeological chronotypology of the excavated findings date the site of Leubingen from c. 3650 calBCE to the Late Middle Ages and Early Modern Period. The oldest burials on the site belong to the Neolithic Baalberge culture and are characterized by trapezoid-shaped burial structures; two burials of this type have been found in Leubingen. Besides the trapezoids, simple individual earth burials could also be attributed to the Baalberge culture. Contemporaneous settlement remains are also known[210]. Further excavations in the vicinity of the tumulus with evidence of a third trapezoid-shaped ditch took place in 2016-2020. The assignment of a child burial described below to the Baalberge culture is a new result.

- **LEU028 (09/100-1782, feat. 2017)-** We recovered *P. vivax* nuclear genome-wide data from a genetically female child of approximately 2.5-3.5 years old. The individual was interred in a round-cornered rectangular grave (0.63 x 0.31 m) within a larger oval pit

(0.85 x 0.70 m). Oriented towards the west-northwest - east-southeast with the head in the west-northwest and facing north, LEU028 was buried in a left-sided crouched position consistent with the Baalberge culture. While pottery sherds accompanying the remains could not be associated with a particular culture, Baalberge-type ceramics were recovered from several nearby archaeological features.

1.2.8 Lungi Tepa

Country: Uzbekistan
Region: Surkhan Darya Province, Sherobod District
Coordinates: 37.897028°, 66.782056°
Sample Date (TGA007): 1500-1900 CE (archaeological context, radiocarbon dating of commingled remains)
Sample Date (TGA012): 1500-1900 CE (archaeological context, radiocarbon dating of commingled remains)
Radiocarbon Date: -
Excavation/Sample Provenance: Excavated in 2017 and 2019 by a joint Czech, Uzbek, and New Zealand team.

Site Description:
Situated near the village of Khojaunkan in the Kugitang foothills of southern Uzbekistan, the site of Lungi Tepa consists of a central oval tell (*tepa*) approximately 60 x 40 x 6.5 m as well as a surrounding settlement area and nearby burial ground[211]. At 1322 masl, the tell overlooks a mountain valley and sits close to a seasonal water course. The tell settlement site is well preserved and has been dated to the Pre-Mongol Medieval Period, whereas the graves on the *tepa* (area T1) have been AMS dated to the Late Medieval period (15th-17th century CE), and the graves adjacent to the *tepa* (area B1 also known as Tell Garden) have been AMS dated to the Late Medieval period and may extend into the post-Medieval period (18th-early 20th century CE)[211]. In 2017 and 2019, excavations by a joint Czech, Uzbek, and New Zealand team recovered human remains that likely represent intact Muslim burials on the *tepa* and commingled remains at the Tell Garden site.

- **TGA007 (Burial 3 2019)**- We recovered *P. vivax* nuclear genome-wide data from a tooth from a subadult, male individual from area B1 (Tell Garden) of Lungi Tepa.
- **TGA012 (Mandible A 2019)-** We recovered *P. vivax* nuclear genome-wide data from a tooth from an adult, female individual from area B1 (Tell Garden) of Lungi Tepa.

1.2.9 Mikulovice

Country: Czech Republic
Region: Eastern Bohemia, Pardubice District
Coordinates: 49.992196°, 15.776734°
Sample Date (MIB002): 2000-1700 BCE, Classical Únětice Culture (archaeological context); 1884-1749 BCE (direct radiocarbon dating); 2013-1770 BCE (contextual radiocarbon dating, from animal bone deposited in the grave of MIB002: CRL-19334, 3548 ± 31 BP, 2013-1770 calBCE, 2-sigma[212])

Radiocarbon Date (MIB002, rib, MAMS-30482): 3494 ± 19 BP, 1884-1749 calBCE (2-sigma)
Excavation/Sample Provenance: Excavated between 2006 and 2012 by J. Frolík (Institute of Archaeology, Academy of Sciences, Prague) and R. Sedláček (Museum Pardubice).
Human Genetic Analysis: Papac *et al.* 2021

Site Description:
The site of Mikulovice consists of a large settlement as well as roughly 100 graves from the Early Bronze Age period. The cemetery was completely analyzed and published in 2020[212,213].

- **MIB002 (Grave 55)-** We recovered *P. vivax* nuclear data from a petrous bone from a c. 20-25 year old female individual buried in grave 55 at Mikulovice. Buried in a right-sided crouched position facing south, the individual was accompanied by rich grave goods including a necklace made of more than 20 amber beads, which were likely imported from the Baltic region, bronze pin, bronze earrings etc.[212].

1.2.10 S'Orcu 'e Tueri (Perdasdefogu)
Country: Italy
Region: Sardinia, Perdasdefogu
Coordinates: 39.6672°, 9.4261°
Sample Date (ORC003): 1370-1130 BCE, Nuragic period (radiocarbon dating)
Radiocarbon Date (ORC003, temporal/petrous bone, MAMS-38282): 2996 ± 24 BP, 1374-1126 calBCE (2-sigma)[131]
Excavation/Sample Provenance: First recovery of human remains in 1963; looting and damage until 2005; salvage surface recovery in 2014 to prevent further looting.
Human Genetic Analysis: Marcus *et al.* 2020

Site Description:
Located in the mountains in the Eastern Sardinian territory of Perdasdefogu (Nuoro), the site of S'Orcu 'e Tueri consists of a natural cave in close proximity to a Nuragic tower and settlement. Since 1963, several interventions have recovered human remains from the site, which has also been subjected to extensive looting. Radiocarbon analysis of eight individuals confirms the cave's use as a burial place during the Nuragic period (c. 1386-991 calBCE), while one individual dates to the later Carthaginian era (391-208 calBCE)[131].

- **ORC003 (busta 12/9-1)-** We recovered *P. vivax* nuclear data from a petrous bone of a genetically male individual ORC003 (busta 12/9-1).

1.2.11 Tarquinia
Country: Italy
Region: Viterbo
Coordinates: 42.25°, 11.7678°
Sample Date (TAQ018): 346-51 BCE, based on relatedness to radiocarbon dated individuals from the same site[214]. No direct radiocarbon dates have been produced for the sample TAQ018 itself.

Radiocarbon Date: -
Excavation/Sample Provenance: Samples were obtained from the collection of Emil Schmidt housed at the Anatomic Institute, Leipzig University.
Human Genetic Analysis: Posth *et al.* 2021

Site Description:
The site of Tarquinia is located near the Tyrrhenian Sea in the contemporary province of Viterbo. A prosperous trade center as early as the 6th century BCE, by the 4th century BCE Tarquinia had developed into the most prominent city in Etruria. The metropolis experienced gradual decline following its conquest by Rome and was eventually abandoned in the 7th century CE[214]. A large, richly appointed necropolis located south of the city on Monterozzi hill was used as a burial place from the 8th century BCE through the Roman period. Excavations of the necropolis began as early as the Renaissance but were not regulated until the later half of the 19th century[214]. The skeletal remains analyzed here were among those purchased by Prof. Emil Schmidt from the curator of the Etruscan tombs in Tarquinia[214]. A catalog compiled by Schmidt in 1887 states that the remains derived from different excavation campaigns between 1877 and 1879, but more precise information regarding archaeological context has been lost[214].

- **TAQ018 (192)-** We recovered *P. vivax* nuclear DNA from the petrous bone of a genetically male individual from the Monterozzi necropolis[214].

1.2.12 Thebes
Country: Egypt
Region: Luxor, Upper Egypt
Coordinates: 25.731733°, 32.597044°
Sample Date (THE006): 800-573 BCE, Third Intermediate Period (radiocarbon dating)
Radiocarbon Date (THE006, temporal/petrous bone, MAMS-43513): 2555 ± 20 BP, 800-573 calBCE (2-sigma)
Excavation/Sample Provenance: Samples were obtained from the collection of Emil Schmidt housed at the Anatomic Institute, Leipzig University.

Site Description:
The city of Thebes, now known as as Luxor, was a crucial center in ancient Egyptian civilization. Its foundation dates back to the Old Kingdom era (c. 2686-2181 BCE), when it was a minor city that subsequently gained significance during the Middle Kingdom (c. 2055-1650 BCE)[215]. Throughout the New Kingdom (c. 1550-1069 BCE), it assumed a pivotal role as an administrative center and served as the capital during the 18th dynasty[216]. However, its influence declined from the Third Intermediate Period (c. 1069-664 BCE) through Roman times as the authority of the cult of Amun waned[216].

The material from Thebes was assembled between 1874 and 1879 by Friedrich Mook (1844-1880), an Egyptologist with doctorates in theology, philosophy, and medicine. Mook collaborated with Dr. Emil Ludwig Schmidt (7 April 1837 – 22 October 1906), a German medical doctor and anthropologist, to whom he sold his collection of skulls from Thebes[217]. Both Mook and Schmidt collected and purchased skulls in Egypt, with Schmidt documenting in his habilitation treatise (1885, 10 f.) that these skulls originated from graves and mummy chambers

in the ancient necropolis of Thebes[218]. However, the detailed context surrounding the individuals analyzed in this study has been lost over time. Between 1885 and 1900 Schmidt was first a private lecturer, then a full professor of anthropology and ethnography at the Philosophical Faculty of Leipzig University. He transferred his skull collection to the University of Leipzig (see https://research.uni-leipzig.de/catalogus-professorum-lipsiensium/leipzig/Schmidt_978/)[217].

- **THE006 (737)-** We recovered *P. vivax* mitochondrial and nuclear data from a petrous bone from an infant of unknown genetic sex.

1.2.13 Voivodeni
Country: Romania
Region: Transylvania, Mureș
Coordinates: 46.704165°, 24.626603°
Sample Date (VOI007): 1425-1465 CE (radiocarbon dating)
Radiocarbon Date (VOI007, MAMS-47793): 447 ± 20 BP, 1425-1465 calCE (2-sigma)
Excavation/Sample Provenance: Professor Z. Bartha discovered the site of Voivodeni in 1975, during fieldwork in a local schoolyard[219]. M. Petică led rescue excavations at the site in 1977 and 1978. The remains analyzed here were recovered during excavations led by N. Man in the summer of 2008, prior to the construction of a new nursery school[219].

Site Description:
Situated on the banks of Luț creek, Voivodeni is located on the eastern side of the Transylvanian Plain approximately 25 km from the city of Târgu Mureș[219]. Excavations by M. Petică in the 1970s unearthed artifacts from periods spanning the Eneolithic through the Middle Ages. The remains analyzed here were recovered in 2008 from a Bronze Age mass burial containing the remains of seven individuals[219]. Initially circular, the burial pit was later cut by several features so that it appeared as an approximately 135 x 120 cm round-cornered rectangle at the time of excavation. Six individuals were interred in fetal position at a depth of approximately 140 cm with a seventh individual at 160 cm. The recovered remains included one adult male of c. 30-35 years of age, two young adult females approximately 14-15 years old, and four children between the ages of six and 13[219]. The grave also included ceramic sherds characteristic of the 3rd phase of the Wietenberg ceramic style[219]. Radiocarbon dates produced from three individuals place the burial between c. 1700-1500 BCE; however, radiocarbon dating for VOI007 (skeleton 7) places this individual in the Late Middle Ages, complicating the burial's chronology.

- **VOI007 (Skeleton 7)-** We recovered *P. vivax* nuclear DNA from a tooth from a genetically female individual interred in a fetal position on her right side facing eastward. The skeleton was poorly preserved with a fragmented cranium.

1.2.14 Volosovo
Country: Russian Federation
Region: Leningrad Oblast, Volosovskiy Rayon
Coordinates: 59.447°, 29.485°
Sample Date (VOS002): 1100-1300 CE (archaeological context)

Radiocarbon Date: -
Excavation/Sample Provenance: The burial ground was excavated in 1876 and 1883 by L. K. Ivanovsky. The bones (skulls) are stored in the Peter the Great Museum of Anthropology and Ethnography (Kunstkamera), Saint Petersburg, as part of collection 5548. A total of 68 skulls from the burial ground are present in it.

Site Description:
In the 1870s and 1880s, over 5500 burial mounds (kurgans) were excavated (L. K. Ivanovsky) in the area of Izhora Plateau in Volosovsky District, Leningrad Oblast, Russia, a region dominated by agricultural lands and temperate deciduous forest. These included a group of 153 mounds in the vicinity of Volosovo village (then Petergofskiy Uyezd, today Leningrad Oblast, Russia). The mounds were up to 3.5 m high and 12 m in diameter and contained burials in a sitting position with funerary implements. Burials of this type are well known in the eastern territory of the Izhora Plateau in the 12th-14th centuries. Based on burial customs and artifacts, the graves from Volosovo were later associated with Votians, a Finnic group, with some possible Slavic component. The Volosovo materials were published by A.A. Spitsyn in 1896[220]. According to the periodization of Y.M. Lesman, the burial ground belongs to the period of the late 12th-early 14th century[221].

- **VOS002 (mound 20, MAE RAS 5548-151)-** We obtained nuclear and mitochondrial *P. vivax* data from an adult male individual from Volosovo[222].

3. Archaeological Site and Sample Information for *P. malariae* Endemic Sites

1.3.1 Sosnovaya griva 3
Country: Russian Federation
Region: Republic of Mari-El, Zvenigovskiy Rayon
Coordinates: 56.164993°, 47.739437°
Sample Date (SOS004): 17th-18th century CE (contextual radiocarbon dating)
Radiocarbon Date: -
Excavation/Sample Provenance: Excavated in 1986 and 1993 by V. S. Patrushev and M. Lavento.

Site Description:
The site of Sosnovaya griva (3) is located within the Bolshaya Kokshaga estuary in the Middle Volga Region (Republic of Mari-El, Russia). It lies in the south of the boreal zone, on a sandy, pine-covered ridge with more than 40 prehistoric settlements and find locations, mostly dating to the Bronze Age[223]. In addition to ten Bronze Age dwellings, excavations at Sosnovaya griva in 1986 and 1993 (V. S. Patrushev, M. Lavento) uncovered at least 13 graves broadly associated typologically with Medieval Finnic Mari people[223]. While precise contextual information for the individual analyzed here has been lost due to storage issues, it certainly belongs to an even later context (c. 17th-18th century CE) based on radiocarbon dates.

- **SOS004-** We recovered *P. malariae* mitochondrial data from the post-medieval individual from Sosnovaya Griva.

4. Archaeological Site and Sample Information for Endemic Sites with Multiple *Plasmodium* Species

1.4.1 Mechelen, St. Rombout's cemetery
Country: Belgium
Region: Antwerp Province, Flanders
Coordinates: 51.029°, 4.4793°
Sample Dates (STR016, STR025, STR045, STR067, STR091, STR105, STR129, STR140, STR185, STR248): c. 1100-1800 CE (archaeological context, stratigraphic observations, radiocarbon dating). Samples belong to three different layers/phases of the cemetery. For further details, including contextual radiocarbon dates, cf. sample description below.
Radiocarbon Date (STR016, tooth, MAMS-62383): 136±17 BP, 1677-1942 calCE (2-sigma)
Radiocarbon Date (STR025, tooth from individual interred in the same single-phase multiple burial, MAMS-34223): 396±20 BP, 1445-1619 calCE (2-sigma)
Radiocarbon Date (STR045, tooth, MAMS-34227): 371±21 BP, 1454-1630 calCE (2-sigma)
Radiocarbon Date (STR067, tooth, MAMS-34236): 258±21 BP, 1527-1797 calCE (2-sigma)
Radiocarbon Date (STR091, tooth from individual interred in the same single-phase multiple burial, MAMS-34239): 343±21 BP, 1476-1636 calCE (2-sigma)
Radiocarbon Date (STR105, tooth, MAMS-34241): post 1650
Radiocarbon Date (STR129, tooth from individual interred in the same single-phase multiple burial, MAMS-34251): 351±22 BP, 1465-1635 calCE (2-sigma)
Radiocarbon Date (STR140, tooth, MAMS-34260): 340±19 BP, 1479-1635 calCE (2-sigma)
Radiocarbon Date (STR185, tooth, MAMS-62382): 1006±17 BP, 991-1120 calCE (2-sigma)
Radiocarbon Date (STR248, tooth, MAMS-62384): 864±17 BP, 1163-1221 calCE (2-sigma)
Excavation/Sample Provenance: Excavated between 2009 and 2011 by the former Department of Archaeology of the city of Mechelen. The remains are currently curated by the Heritage Depot Rato of the city of Mechelen, Belgium.
Human Genetic Analysis: Analysis of 40 individuals presented in this study.

Site Description:
The cemetery surrounding St. Rombout's parish church served as a burial ground in the small city of Mechelen, Belgium, between the 10th and 18th centuries CE. Excavations at St. Rombout's cemetery between 2009 and 2011 unearthed the remains of 4,158 individuals in anatomical position from 3,617 graves, deriving from three layers roughly dated to the 12th-14th century CE, the 15th-16th century CE, and the 17th-18th century CE[57,58]. The oldest layer also contains a number of burials which have been dated to the 10th-11th centuries CE. Many individuals interred at the cemetery were likely inhabitants of St. Rombout's parish, one of the wealthiest in the prosperous city of Mechelen. However, poor and sick individuals in the care of the parish and/or charitable institutions may also have been buried there. Furthermore, St. Rombout's cemetery may have served as a burial place for patients at the adjacent Spanish military hospital, the first permanent military hospital in early modern Europe. First instituted in 1567 and mainly active between 1585 and 1715, the hospital may have treated as many as 2000 soldiers per year, but no associated hospital cemetery has yet been identified[57,58].

In ancient, medieval and early modern Europe, multiple burials, simultaneous interments of several individuals in a single grave, have often been associated with periods or events of unusual mortality[224]. The excavation of St. Rombout's cemetery revealed a total of 65 multiple

graves, which were spatially dispersed across the cemetery and appear more common in later time periods (15th-18th century CE)[57]. A detailed study of 351 individuals from the parish cemetery included 19 multiple burials containing a total of 103 individuals, with the number of individuals per grave ranging from two to 14[58]. Demographic composition, paleopathological changes, and burial characteristics suggest that the multiple graves may harbor individuals from particular subgroups of the population, distinctive with respect to contemporaneous single burials. In particular, the multiple burials harbor an overrepresentation of males and a larger proportion of adolescents and young adults than expected for attritional burials, and the frequency of markers of physical stress, growth disturbances, and disease differ between the multiple burials and contemporary single depositions[58]. Based on this evidence, the multiple graves may include soldiers treated at the adjacent military hospital or young laborers/apprentices of lower socio-economic background[58].

Here we report evidence for *Plasmodium* infection in teeth from ten individuals from three temporal phases of St. Rombout's parish cemetery. In addition to analyzing *Plasmodium* spp. DNA, we assessed the human genetic ancestry of a set of 40 individuals interred in the three burial phases at St. Rombout's (**Supplementary Methods 10**). This set of individuals included all ten of the *Plasmodium*-positive individuals described below. Metadata for all individuals included in the population genetic analysis can be found in **Supplementary Table 7**.

Early Phase (c. 12th-14th century CE)

- **STR185 (614-1):** We recovered *P. vivax* mitochondrial and nuclear data from a genetically female adult individual interred in a single burial contextually dated to the 12th-14th century CE.
- **STR248 (2814-1)-** Interred in a double burial with an adult, STR248 is a male child contextually dated to the 12th-14th century CE. We obtained *P. vivax* nuclear data from this individual.

Middle Phase (c. 15th-16th century CE)

- **STR025 (261-1)**: Analysis of STR025 yielded genomic mitochondrial and nuclear data from both *P. falciparum* and *P. vivax*, suggesting a multispecies malarial coinfection in this individual. STR025 is a genetically male individual aged 18-25 years buried in a single-phase multiple burial containing a total of 10 individuals, including three adolescents aged 12-17, five male adults aged 18-25, one 18-25-year-old of indeterminate sex, and one middle-aged adult male aged 26-50.
- **STR045 (287-5)-** STR045 is a male individual aged 26-50 years, interred in a single-phase multiple burial containing 12 individuals, including six adolescents (approximately 12-17 years old), four young adults (2 male, 1 female, and 1 of indeterminate sex, between 18-25 years old), and two middle aged adults between about 26-50 years old (one male, one female). We recovered *P. vivax* nuclear DNA from this individual.
- **STR091 (1922-3)-** Buried in a single-phase multiple burial which included four non-adults and three adults (based on the initial assessment), the genetically male, adult individual STR091 exhibited evidence of a *P. falciparum*/*P. malariae* coinfection.

- **STR129 (3069-5)-** We recovered *P. vivax* nuclear data from STR129. This adult male individual was interred in a single-phase multiple-burial, which included two non-adults and six adults (based on the initial assessment).
- **STR140 (3535-2)-** Interred in a single-phase multiple burial which included one non-adult and four adults (based on the initial assessment), this adult male individual exhibits evidence of a *P. malariae* infection.

Late Phase (c. 17th-18th century CE)

- **STR016 (203-7)-** Dated to the late 17th-18th century CE based on archaeological context, individual STR016 was a male between 18-25 years, who suffered from a *P. falciparum*/*P. malariae* coinfection. STR016 was interred in a multi-phase multiple burial containing four adolescents of c. 12-17 years old, five young adult males between c. 18-25 years old, and one adult of indeterminate age and sex.
- **STR067 (569-2)-** STR067 is a genetically male individual, aged 18-25 years, interred in a possible multi-phase multiple burial. We identified *P. vivax* nuclear DNA in STR067.
- **STR105 (1957-5)**: Analysis of a tooth from STR105 yielded *P. vivax* mitochondrial and nuclear DNA. STR105 is a genetically male individual recovered from a single-phase multiple burial which included five adults (based on the initial assessment).

Supplementary Note 2. Mitochondrial Capture Analysis

To evaluate the success of our mitochondrial capture and identify the species present, we performed a competitive mapping against a concatenated reference containing *P. falciparum*, *P. vivax*, and *P. malariae* reference genomes (**Supplementary Methods 4**). For each species, we counted the number of libraries with more than 100 unique reads assigned to the corresponding reference; using this threshold, we identify 15 libraries from 13 unique individuals as positive for *P. falciparum*, four libraries from four unique individuals with evidence of *P. malariae* preservation, and nine libraries from six individuals infected by *P. vivax*. The number of competitively assigned reads varied significantly across libraries, with a maximum of 6692, 5304, and 5452 reads assigned to *P. falciparum*, *P. vivax*, and *P. malariae*, respectively (**Supplementary Table 14**).

For 23 out of 26 putatively *Plasmodium*-positive libraries, more than 90% of competitively mapped reads were assigned to a single species, as expected for a monoinfection. The predominance of a single species for most libraries suggests that mapped reads derive from endogenous *Plasmodium* DNA rather than distantly-related environmental contaminants, which we would expect to be equally related to the three species included in our competitive mapping. Interestingly, we do identify three probable cases of coinfection by more than one *Plasmodium* spp. Individual STR016 had 490 and 1482 reads assigned to *P. falciparum* and *P. malariae* respectively, while STR091 exhibited 464 and 102 reads competitively mapped to these two species. On the other hand, two libraries prepared for individual STR025 showed different species predominating; library STR025.A0101 had 83.4% (n=283) of reads assigned to *P. vivax*, while STR025.A0102 had 96.1% (n=708) of reads assigned to *P. falciparum*. Similarly, we observe some stochasticity in *Plasmodium* mitochondrial DNA recovery across capture

experiments; one of two and one of three libraries prepared for the *P. falciparum*-positive individuals GBA004 and CHO001 failed to meet our threshold of 100 unique mapped reads for a putatively positive library. We suggest that this stochasticity in capture performance across libraries prepared from the same sample may result from the extremely short length and relatively low copy number of *Plasmodium* mitochondrial genomes, as random loss of fragments during purification steps may have a significant effect on the proportion of the genome that can be reconstructed.

Next, we applied several quality control metrics to verify that the recovered DNA derives from endogenous ancient *Plasmodium* spp. First, we computed a mapping specificity score (m, described above) to test whether environmental contamination may account for the observed alignments. A lower mapping quality threshold (e.g. -q 0) results in retention of reads that map with equal likelihood in multiple locations across the concatenated reference genome, while a strict mapping quality threshold (e.g. -q 37) selects for alignments specific to a particular genomic location. In a true *Plasmodium* infection, we expect a significant proportion of species-specific alignments, resulting in a low value of m, while environmental contaminants should exhibit a higher value. Indeed, for each single-species infection passing our inclusion thresholds, the species present consistently exhibits the lowest m value, ranging from 1.2 to 1.87 (**Supplementary Table 14**). Species not identified as part of a mono- or coinfection, on the other hand, exhibit m values ranging from 3.35 to 1329.

Finally, we extracted alignments from putatively-positive species falling outside of problematic regions described above and used DamageProfiler to evaluate these for the presence of characteristic ancient DNA damage patterns. We observe 5' C to T substitution values consistent with those expected for UDGhalf and non-UDG treated libraries (**Supplementary Table 14**). Notably, two of the UDG-half libraries with 5' deamination values below 5% come from archaeological contexts consistent with low damage rates. CSR001 dates to very recent times (16th-mid 19th century CE) and was recovered from a cathedral crypt. THE006 is a mummified individual dating to 800-573 calBCE in Egypt, and previous ancient DNA studies of Egyptian mummies have noted exceptionally low DNA damage rates possibly related to the mummification process[225]. Several additional low-coverage libraries displaying abnormal damage profiles have been flagged; as all three samples have additional, higher coverage libraries exhibiting damage patterns consistent with endogenous ancient DNA, we include these datasets in subsequent analyses. Finally, we compare 5' C-T substitution rates on reads mapping to *Plasmodium* mitochondria and the human genome. If the ancient *Plasmodium* reads derive from an active infection at the time of death, we hypothesize that we should observe similar deamination rates on reads derived from the human and pathogen genome. Indeed, we note a strong correlation for all three *Plasmodium* spp., with generally higher deamination rates on reads mapping to *Plasmodium* spp. compared to the human reference genome (**Extended Data Figure 4; Supplementary Table 14)**.

Next, we merged data from libraries passing quality control thresholds on a per-sample basis for genomic analysis (**Supplementary Table 2**). For all three species, we note a small number of substitutions in the ancient mitochondrial genomes compared to the reference, which is consistent with the short length of the *Plasmodium* mitochondrial genome (c. 6000 bp). As low sequence divergence obscures branching patterns in phylogenetic analysis, we visualized patterns of haplotype diversity using median joining networks as implemented in PopART (http://popart.otago.ac.nz). For *P. falciparum* (**Extended Data Figure 1**), six genomes attained sufficient coverage for inclusion in our network analysis (**Methods**): Ebro1944, CSR001,

GBA004, GOE016, HPD007, and LIP011. Interestingly, two of the European strains (Ebro1944 and GOE016) harbor the same mitochondrial haplotype, which still circulates in Asian *P. falciparum* populations today. Haplotypes from strains HPD007 and LIP011 are closely related to the Ebro1944/GOE016 haplotype, exhibiting one and two nucleotide differences, respectively. CSR001 and GBA004 show relatedness to large haplotype groups that are widely distributed across multiple continents. For *P. vivax* (**Extended Data Figure 1)**, four genomes attained sufficient coverage for network analysis: Ebro1944, LDC020, STR105, and THE006. The two European strains, STR105 and Ebro1944, harbor the same mitochondrial haplogroup. LDC020 exhibits a common haplogroup that is globally distributed across multiple continents. Overall, *Plasmodium* mitochondrial haplotypes exhibit a low phylogeographic signal, limiting the inferences that can be drawn regarding parasite transmission from mitochondrial data alone.

Supplementary Note 3: Nuclear Capture Analysis

As with the mitochondrial analysis, we assessed the competitive mapping of the ancient nuclear datasets to determine the success of our capture experiments and identify the *Plasmodium* spp. present. Employing a cutoff value of 1,500 target SNPs, we identify 12 libraries from eight unique individuals exhibiting evidence of *P. falciparum* DNA preservation, while 26 libraries from 22 ancient individuals attain the threshold for *P. vivax* positivity (**Supplementary Table 15)**. As in the mitochondrial capture experiments, we observe several libraries with putative *Plasmodium* spp. coinfections. For STR025.A0102, 9,497 (68.36%) and 4,396 (31.64%) covered target SNPs belong to *P. falciparum* and *P. vivax*, respectively. A second library from this individual, STR015.A0101, narrowly missed the threshold for *P. falciparum* positivity (1459 SNPs, 36.13%) but had 2,579 (63.87%) *P. vivax* target SNPs covered. Interestingly, these results parallel our findings for the mitochondrial capture experiments, where *P. vivax* reads predominated for STR025.A0101, while STR025.A0102 preserved more *P. falciparum* DNA. In addition to the *P. vivax/P. falciparum* coinfection, one library (STR091.A0103) exhibiting a *P. falciparum/P. malariae* coinfection in our mitochondrial capture analysis exceeded the *P. falciparum* positivity threshold following nuclear capture. Notably, this library also exhibited coverage of 1,249 *P. vivax* target nuclear SNPs but appeared *P. vivax*-negative following mitochondrial capture. We suggest that coverage of *P. vivax* nuclear SNPs in STR091.A0103 may be due to off-target capture and mapping of *P. malariae* DNA in our nuclear capture experiment.

We note that libraries attaining our positivity threshold exhibit a high proportion of on-target reads; for libraries with *P. falciparum* monoinfections, between 65.37 and 89.74% of reads competitively mapped to the 14 chromosomal scaffolds covered one or more target SNP; for *P. vivax*, the on-target read percentage ranged from 63.89 to 87.61% (**Supplementary Table 15**). In contrast, on-target rates tend to be lower for samples failing to attain positivity thresholds for a given species. The target SNP panels have been filtered to include only high-quality variants and exclude regions of the genome that may be particularly prone to mismapping, such as the centromeres, subtelomeric regions, and internal hypervariable regions. Thus, the high proportion of on-target reads supports the authenticity of our ancient *Plasmodium* spp. genotype data.

To test whether nuclear SNP calls could be the result of mismapping from environmental taxa, we computed a mapping specificity score (m) comparing the number of reads competitively

mapped to each species with loose (-q 0) and strict (-q 37) mapping quality thresholds. A low score indicates that a large proportion of reads map with highest quality in a single genomic location, whereas a high score indicates non-specific mapping, possibly from environmental contaminants. As with the mitochondrial capture results, we observe that samples meeting our positivity thresholds display lower m values, ranging from 1.2-4.5 (mean 2.3) and from 1.0-3.2 (mean 1.8) for putative *P. falciparum* and *P. vivax* monoinfections, respectively (**Supplementary Table 15**). In contrast, the average *P. vivax* specificity score for putative *P. falciparum* monoinfections was 8.5 (range 5.7- 14.1), while the mean *P. falciparum* specificity score in samples classified as *P. vivax* monoinfections was 16.6 (range 3.6 to 40.8).

Next, we re-analyzed the nuclear capture data using a MALT filtering step to extract reads taxonomically assigned at or below the Laverania and *P. vivax* nodes prior to competitive mapping. We rationalized that this pre-filtering step should reduce species misidentifications due to mismapping from closely-related taxa included in the MALT fullNT database but not in our competitive mapping reference. Encouragingly, we observe that pre-filtering with MALT does not greatly reduce the number of target SNPs covered for individuals classified as *P. falciparum* and *P. vivax* monoinfections, further supporting the endogenous nature of the *Plasmodium* spp. nuclear data recovered (**Supplementary Table 15**).

Finally, we analyzed reads overlapping *Plasmodium* target SNPs with MapDamage to check for patterns of nucleotide substitution and fragmentation characteristic of ancient DNA (**Supplementary Table 15**). For libraries classified as *P. vivax* and/or *P. falciparum*-positive, 5' C to T substitution rates were within the range of those expected for ancient data according to library preparation protocol. Next, we investigated whether human and *Plasmodium*-mapping reads from the same library exhibit similar damage and fragmentation patterns, which would further support their authenticity as endogenous ancient *Plasmodium* infections. For the majority of samples, we observe strong correlations between both substitution rates and average fragment lengths for reads mapped to *P. vivax* and/or *P. falciparum* and the human reference genome (**Extended Data Figure 4; Supplementary Table 15**).

Interestingly, we note that a subset of *P. vivax*-positive nuclear capture libraries exhibit unique damage properties compared to the remaining *P. vivax* and all *P. falciparum*-positive samples. These libraries also failed to meet our cutoff value for *P. vivax* mitochondrial positivity after capture and are hereafter referred to as the nuclear-only *P. vivax* dataset. For these libraries, damage rates on reads mapping to the *P. vivax* reference are significantly higher than expected based on human-mapping reads. Indeed, C to T substitution rates at the first base on the 5' end reach as high as 45-50%, suggesting that a majority of *P. vivax*-mapping reads in these individuals may in fact be single-stranded. These same libraries exhibit systematically shorter mean fragment lengths than off-target human alignments. Finally, in contrast to the remainder of our dataset, there appears to be no correlation in mean fragment length between *P. vivax* and human reads for the nuclear-only *P. vivax* libraries. Together, these observations suggest that *P. vivax* reads from nuclear-only *P. vivax* libraries are systematically more damaged than in the remainder of our dataset. Further investigation is needed to clarify the physiological processes that lead to our recovery of *Plasmodium* DNA from skeletal material and may account for the observed differences in damage/fragment lengths in *P. vivax* DNA (**Extended Data Figure 4**).

Supplementary Note 4: Nuclear Downsampling Simulations

To determine appropriate *Plasmodium* nuclear SNP coverage thresholds for inclusion in subsequent analyses, we evaluated the performance of downsampled modern datasets using smartPCA (**Supplementary Methods 9**). For both *P. falciparum* (**Extended Data Figure 5**) and *P. vivax* (**Extended Data Figure 5**), we observe that simulated genomes downsampled to 5,000 segregating SNPs project very close to the original, full-coverage modern strain. This result reflects a robust ability to distinguish population of origin even with a high amount of missing data, corresponding to 95.3% and 98.8% of segregating sites for *P. falciparum* and *P. vivax*, respectively.

At coverage levels of 1500 and 500 segregating SNPs, downsampled replicates project across much broader spatial distributions. Convex hulls encompassing replicates from related populations overlap in many cases, reflecting a limited ability to distinguish between closely-related populations at these extremely low coverage levels. However, we note that even with only 500 SNPs covered (corresponding to 0.5% and 0.1% of segregating sites for *P. falciparum* and *P. vivax,* respectively), replicates from regional population groupings tend to fall in distinct regions of the PCA, reflecting sufficient genetic signal to distinguish between African/South American/Western Asian populations, Southeast Asian populations, and Oceanian/Maritime Southeast Asian populations in many cases. Based on this analysis, we conclude that 500 segregating SNPs represents an appropriate threshold for inclusion in our *P. vivax* and *P. falciparum* nuclear population genetic analysis; however, we note that precise spatial positioning should be interpreted with significant caution at this level of coverage. Below 500 segregating SNPs, the spatial distributions of downsampled replicates overlap to such an extent that we consider these data uninformative for assessing population affinities.

Supplementary Note 5: *P. vivax* Population Genetic Analysis

As a quality control check of our analysis pipeline, we attempted to replicate patterns of modern *P. vivax* population structure observed in previous studies. First, we performed a principal component analysis using modern genotype data from the MalariaGEN *Plasmodium vivax* genome variation project. As previously observed, we find evidence of strong geographical structure in modern *P. vivax* populations, with strains from proximal regions forming distinct clusters in PCA space (**Figure 2; Extended Data Figure 3**)[37]. The two main axes of variation in our PCA define three genetic clusters, including clones from Africa, Western Asia, and Latin America, strains from East and Southeast Asia, and strains from Oceania. Maritime Southeast Asian strains, including clones sampled from Malaysia and the Philippines, form a cline between Southeast Asian and Oceanian strains (PC2). Similarly, two clones sampled from Bhutan fall halfway between the African/Western Asian/Latin American cluster and the East/Southeast Asian cluster, suggesting admixture between these two sources. The third principal component, which accounts for 3.23% of variation present in the data, separates Latin American populations from African, Ethiopian, and Western Asian strains (**Extended Data Figure 3**).

The results of an unsupervised ADMIXTURE analysis (**Figure 2**) support the modern population differentiation observed in PCA. Modern *P. vivax* strains are best modeled using K=6 ancestral sources, which are each maximized in one of the following populations: Latin America, Oceania, Eastern Southeast Asia, Western Southeast Asia, Western Asia, and Ethiopia.

Accordingly, and consistent with the putative admixture cline observed in PCA, Maritime Southeast Asian strains are modeled as mixtures of Oceanian, Western Southeast Asian, and Eastern Southeast Asian ancestry, while the two strains from Bhutan are modeled as mixtures of Western Asian and Western Southeast Asian ancestry. The African and East Asian/Northern Southeast Asian clusters include geographically diverse strains annotated with an "Unknown" population label in the MalariaGEN data release; while we group these strains based on geography to facilitate downstream analysis, we observe genetic heterogeneity within these clusters consistent with their diverse sample origins.

To assess the genetic affinities of our ancient samples, we project these strains onto the PCA computed using the modern *P. vivax* populations. Based on the results of our downsampling analysis, we consider 5,000 segregating SNPs an appropriate threshold for reliably differentiating strains from distinct populations. We include lower-coverage samples in the PCA as well (**Extended Data Figure 3**), but caution that we expect a significant degree of uncertainty in PCA position at coverage levels approaching 1,500 SNPs and lower. Interestingly, we find that the only South American strain in our ancient dataset, LDC020, overlaps the modern Latin American cluster in PCA space, while high-coverage European samples with more than 5,000 SNPs also fall close to the Latin American cluster along PCs 1 and 2. Lower quality European strains with coverage levels between ~700 and 3500 segregating SNPs fall in a diffuse group adjacent to the higher coverage European samples. In projections of the first and third principal components, we observe greater differentiation of the Latin American and pre-elimination European clusters. On the plot of PC1 and PC2, two strains from the High Medieval period in Uzbekistan (Lungi Tepa) fall adjacent to modern Western Asian strains, on what might be interpreted as the proximal end of the cline separating the African/Western Asian/Latin American and the East/Southeast Asian clusters. Finally, despite extremely low coverage (n=721 SNPs), one strain from Thebes from the Third Intermediate Period overlaps with the modern Western Asian cluster, which includes strains from Afghanistan, India, Iran, and Sri Lanka. (**Extended Data Figure 3**).

To explore these genetic patterns within a model-based framework, we performed a supervised ADMIXTURE analysis, modeling the ancient strains using the aforementioned six populations with distinct ancestral sources identified in the unsupervised analysis at K=6: Latin America, Oceania, Eastern Southeast Asia, Western Southeast Asia, Western Asia, and Ethiopia (**Figure 2; Extended Data Figure 3**). Consistent with the results from PCA, LDC020 (n = 208,344 SNPs) is modeled as deriving 100% of its ancestry from Latin America. The European strains also derive the majority of their ancestry from Latin America, while the samples with highest coverage, Ebro1944 (n = 261,814) and STR105 (n = 57,921), are modeled as deriving 13.58% (± 2.5%, 3 SE) and 14.15% (± 4.5%, 3 SE) of their ancestry from a Western Asian source, respectively. In contrast, GAT004 (n = 10,101) is modeled as deriving ~9% (± 6.2%, 3 SE) of its ancestry from Eastern Southeast Asia. Analysis of additional European samples is needed to determine whether this observation results from low coverage or reflects a real history of connectivity between East Asian and European *P. vivax* populations. Finally, consistent with its position in PCA space, the Medieval Uzbekistan strain TGA007 (n = 5,819) derives the majority of its ancestry from Western Asia, with a minor component most closely related to modern Latin America (22.95%, ± 16.8%, 3 SE).

To quantitatively assess the affinity of our ancient strains to modern *P. vivax* populations, we used outgroup F_3-statistics to measure the shared drift between ancient and modern populations relative to the outgroup *Plasmodium vivax*-like. We find that LDC020 shares

significantly more drift with Latin America compared to any other modern population (**Extended Data Figure 3**). After Latin America, LDC020 shares most drift with STR105 and Ebro1944, further supporting the close relationship between Latin American and ancient European strains. Similarly, Ebro1944 and STR105 share more drift with one another and with ancient and modern Latin American samples than other modern *P. vivax* populations (**Extended Data Figure 3**). These results are further supported by cladality tests of the form f_4(*P. vivax*-like, Ancient Test; LAM, Modern) (**Supplementary Table 4**). Inclusion of LDC020, Ebro1944, or STR105 breaks the cladality of LAM and any modern population tested (≤-3 s.e. or Z≤-3), indicating a genetic affinity of Latin American populations with the ancient Latin American/European strains that is not shared with any other modern group. In addition, f_4(*P. vivax*-like, Ebro1944; LDC020, LAM) is zero (1.06 s.e.), whereas f_4(*P. vivax*-like, LDC020; Ebro1944, LAM) is positive (i.e., 4.3 s.e. or Z=4.3), providing further support for LDC020 being a sister clade to LAM compared to other ancient strains.

Next, we used MEGA-CC to reconstruct a neighbor-joining phylogeny incorporating both modern and ancient *P. vivax* (**Figure 2**). The presence of meiotic recombination in the life cycle of *Plasmodium* spp. presents a significant complication for phylogenetic analysis, as recombination events cannot be easily modeled by a bifurcating tree. Consistent with this observation, few nodes receive high bootstrap support values, indicating uncertainty in the tree topology (**Figure 2**). Similarly, despite removing singletons from our SNP set, we observe long terminal branch lengths for modern strains; these long branch lengths indicate a significant number of homoplasious SNPs, which may result from processes such as admixture, incomplete lineage sorting, etc. Despite these challenges, we note that strains from the same geographic region fall together in our neighbor joining tree, suggesting that phylogenetic analysis still provides a useful approach for exploring patterns of population affinity in *Plasmodium* spp. We observe that LDC020 falls within a clade composed of modern Latin American strains, suggesting that this ancient sample represents a close relative of pathogens circulating in the Americas today. Moreover, we find that Ebro1944, STR105, STR185, and GAT004 all fall basal to the Latin American branch, reinforcing the suggested directionality of *P. vivax* transmission from Europe to the Americas during the colonial period. Consistent with other analyses, TGA007 falls with strains from modern Western Asia and Bhutan in the tree.

Supplementary Note 6: *P. vivax* Population Structure Within Latin America

In order to further explore Latin American *P. vivax* population structure, we performed a principal component analysis on 62,820 SNPs segregating in a subset of 96 modern clones. We observe phylogeographic structure in modern Latin American *P. vivax* strains, with clones from Brazil, Colombia, Peru, and Central America (Nicaragua, Panama, Mexico, and El Salvador) forming distinct clusters in PCA space (**Extended Data Figure 3b**). Based on this result, we consider these groups as distinct subpopulations in subsequent analyses. Next, we projected the ancient samples LDC020 and Ebro1944 onto these axes of variation. Both LDC020 and Ebro1944 fall close to the origin, which may be due to the higher proportion of missing data in our ancient datasets. However, we note that LDC020 falls near the cluster of modern Peruvian strains, possibly reflecting a genetic link between ancient and modern populations in this region.

As we observe genetic heterogeneity within Latin American *P. vivax* lineages, we next sought to determine whether these populations diverged from a single introduced lineage or

derive from the mixture of multiple genetically heterogeneous founding populations. In the former case, we expect pairs of Latin American subpopulations to be cladal relative to all other modern *P. vivax* groups. We tested this hypothesis using F_4-statistics of the form f_4(*P. vivax*-like, Test; LAM1, LAM2). Interestingly, we observe significantly higher allele sharing between *P. vivax* populations from Colombia, Peru, and Brazil and WAS, ETH, and AF compared to Central American populations (**Supplementary Table 5**). Brazilian strains also share excess affinity with ESEA, WSEA, and EA_NSEA compared to several other Latin American subpopulations (**Supplementary Table 5**). Based on these results, we infer that modern Latin American *P. vivax* experienced a complex population history including the introduction of multiple genetically distinct strains. Our results would be consistent with the introduction of a single European *P. vivax* population followed by subsequent admixture with strains related to WAS/ETH/AF and ESEA/WSEA/EA_NSEA. Alternatively, present-day Latin American *P. vivax* strains may have descended from multiple European sources with asymmetrical relatedness to Eurasian *P. vivax* strains. More thorough sampling of pericontact *P. vivax* clones from Europe may help to discriminate between these two scenarios.

Finally, to investigate the relationship between the ancient strains LDC020, Ebro1944, and STR105 and modern Latin American subpopulations, we computed F_4-statistics of the form f_4(*P. vivax*-like, Ancient; LAM1, LAM2). We observe that all pairs of modern Latin American subpopulations are cladal with respect to STR105 and Ebro1944. On the other hand, we note that LDC020 exhibits excess allele sharing with modern Peruvian *P. vivax* strains relative to any other Latin American subpopulation (Brazil, Colombia, Central America). This shared ancestry suggests the establishment of an endemic focus in Peru soon after the contact period, and some ancestry related to this early lineage appears to persist in *P. vivax* clones in the region today.

Supplementary Note 7: *P. falciparum* Population Genetic Analysis

As with *P. vivax*, we first verified that patterns of modern *P. falciparum* population structure mirror the results of previous studies. In general, we observe a lower amount of diversity in *P. falciparum* populations compared to *P. vivax*, with 106,179 segregating SNPs in a geographically diverse set of 1,227 *P. falciparum* strains compared to 419,387 SNPs in a set of 906 *P. vivax* clones. Consistent with this lower level of nucleotide diversity, geographically proximal populations show some overlap in PCA space. For *P. falciparum*, principal components 1 and 2 capture 9.95 and 1.85% of the variation present, respectively, and define three main clusters: 1.) Africa and South America, 2.) Southeast and South Asia, and 3.) Oceania (**Figure 3; Extended Data Figure 8**)[38]. Interestingly, we observed that strains annotated as Oceania (OCE) in the Pf6 data release appear to form two partially overlapping clusters in PCA space. Upon further investigation, we found that these two clusters were composed primarily of clones from Papua New Guinea (PNG) and Indonesia, respectively, indicating phylogeographic substructure within the OCE strains. Finally, as the Pf6 data release of the *P. falciparum* Community Project lacks data from India, we included five shotgun-sequenced *P. falciparum* datasets obtained from hospitalized patients in Goa alongside the MalariaGEN genotype data[45]. We find that these Indian clones form a distinct cluster in PCA space, falling closest to South Asian strains from Bangladesh and slightly shifted towards Oceania along PC2 (**Figure 3, Extended Data Figure 8**).

Using an unsupervised ADMIXTURE analysis, we replicate patterns of modern *P. falciparum* population structure observed in PCA (**Extended Data Figure 8**). Based both on analysis of CV errors and correspondence of K to geographical substructure, we selected a model in which *P. falciparum* clones constitute mixtures of K=8 ancestral source populations, forming the following clusters: Central/East Africa (CAF/EAF), West Africa (WAF), South America (SAM), Indonesia, Papua New Guinea (PNG), Eastern Southeast Asia (ESEA), Western Southeast Asia (WSEA), and South Asia/India (SAS/IND). As we observe separation between PNG and Indonesia in both PCA and ADMIXTURE, we include these clones as separate populations in subsequent analyses. All other population labels follow those published in the *P. falciparum* Community Project Pf6 data release.

To assess the genetic affinities of our ancient *P. falciparum* strains, we projected our datasets onto a PCA of modern clones computed as described above. Three ancient samples exceed our 5,000 SNP coverage threshold for reliably differentiating strains from distinct populations: Ebro1944, HPD007, and CHO001. Interestingly, all three high-coverage samples fall into a gap in PCA space, situated closest to the Indian shotgun datasets and with PC2 values intermediate between South/Southeast Asian and Oceanian populations (**Figure 3**). Such a positioning is perhaps surprising, considering the geographically and temporally disparate origins of the three ancient strains, which derive from archaeological sites in Spain, Taiwan, and Nepal and span almost three millennia of human history. However, such a result is consistent with previous studies of the Ebro1944 genome, which shows the highest similarity to modern South Asian clones[34]. Indeed, South Asia has been proposed as a source for *P. falciparum* in Europe, which may have been introduced as a result of cross-cultural connectivity in the first millennium BCE. Similarly, as HPD007 comes from colonial-era Taiwan, the *P. falciparum* strain recovered from that individual could conceivably derive from European, East Asian, or island Taiwanese parasite populations, none of which is well-represented in currently sampled modern datasets. An additional five European strains with coverage levels between 500 and 5,000 segregating SNPs form a diffuse cluster surrounding the high coverage ancient clones (**Extended Data Figure 8**).

To further explore these findings within a model-based framework, we performed supervised ADMIXTURE modeling our ancient strains as mixtures of K=8 ancestral sources, each maximized in one of the following modern populations: East Africa, West Africa, South America, Indonesia, Papua New Guinea, Eastern Southeast Asia, Western Southeast Asia, and South Asia. In contrast to the results for *P. vivax*, none of our three high coverage clones can be modeled as deriving a majority of its ancestry from a single modern source population (**Extended Data Figure 8**). In fact, both CHO001 and Ebro1944 show affinity to five modern source populations, including South America, Eastern Southeast Asia, Papua New Guinea, Indonesia, and South Asia, possibly indicating that they derive from a population not represented amongst currently sampled modern sources. To explore this hypothesis, we repeated our supervised ADMIXTURE analysis with K=9 populations, including the eight sources listed above and Ebro1944. For this analysis, we downsampled our modern dataset to include only one randomly selected clone per population and performed our inference using the intersection of pruned SNPs and those genotyped in Ebro1944. Interestingly, both high and low-coverage ancient clones are modeled as deriving a large proportion of their ancestry from Ebro1944. Importantly, this finding likely does not represent a batch effect, as libraries from Ebro1944 were generated in a different laboratory via shotgun sequencing rather than capture.

To further explore the origins of *P. falciparum* in the Americas, we used *F*-statistics to explore genetic affinities between modern South American, modern African, and ancient European clones. F_3-statistics of the form f_3(WAF; Ancient, Modern) demonstrate that Ebro1944, HPD007, and CHO001 share more drift with Asian populations compared to South American strains (**Extended Data Figure 8**). This finding remains true whether EAF or CAF are selected as the outgroup rather than WAF. On the contrary, we find evidence for cladality between modern African and South American *P. falciparum* populations. At three standard errors, values for f_4(*P. praefalciparum*, Test; AF, SAM) are not significantly different from zero for any ancient and/or modern population *Test* population; these findings suggest that South American *P. falciparum* populations descended from African lineages, possibly as a result of the trans-Atlantic slave trade. However, we note that tests of the form f_4(*P. praefalciparum*, Ebro1944; SAM, AF) approach significance when AF includes CAF (Z = -2.984), WAF (Z = -2.948), and EAF (Z = -2.891) (**Supplementary Table 6**). As such, we cannot exclude some contribution of European strains to the genetic diversity of modern South American populations.

Finally, we used MEGA-CC to construct a neighbor-joining phylogeny including both ancient and modern *P. falciparum* strains (**Extended Data Figure 8**). As with *P. vivax*, a majority of nodes in the resulting tree receive low bootstrap support, indicating uncertainties in tree topology. Nevertheless, we find that the ancient genomes Ebro1944, HPD007, and CHO001 fall with Asian rather than African *P. falciparum*, a result consistent with our principal component analysis. Indeed, our ancient genomes form a sister clade to genomes from modern India[45]. Further sampling will be needed to clarify these results, given the paucity of data from contemporary India and uncertainties in our tree topology.

Because of the lower amount of diversity in *P. falciparum,* we tested for possible discrepancies using a different clustering algorithm provided through ChromoPainter/fineSTRUCTURE (http://www.paintmychromosomes.com/)[226]. This software harnesses haplotype information to 'paint' every sample in the analysis with the haplotypes of the remaining samples in the dataset. In the absence of a recombination map or if the data are widely spaced enough to be considered unlinked, the copying model runs independently for every biallelic marker. Irrespectively of a linked or unlinked model, the input for this analysis cannot include missing data, therefore the analysis was run including only Ebro1944, the ancient *P. falciparum* strain with the highest coverage, and after a series of filters with plink2 were applied to eliminate missingness from the modern dataset (--bfile --geno 0.01, --bfile –mind 0.003)[98]. After these filters, 1067 modern individuals and 47453 segregating biallelic SNPs were retained. More specifically, the strains from India and most of the Indonesian ones were excluded from the analysis input. The entire pipeline from ChromoPainter to fineSTRUCTURE was run with one command (fs) with the options "-ploidy 1 -go". The output was analyzed in RStudio, using the R functions implemented by the developers and available at http://www.paintmychromosomes.com. FineSTRUCTURE estimated k=61 (**Extended Data Figure 8**); with the algorithm of additional hill-climbing moves embedded in the software, the populations were organized in a tree that broadly corresponds to the geographical substructure previously described, and Ebro1944 forms a separate branch within the clade of SAS strains. Similar to smartpca, the eigenanalysis of the normalized version of the coancestry matrix implemented in the R code does not place Ebro1944 within any of the modern clusters, however, it falls closer to strains from South Asia (SAS) and Southeast Asia (WSEA and ESEA) (**Extended Data Figure 8**). When GLOBETROTTER was performed on the *chunkcounts.out output from ChromoPainter/fineSTRUCTURE using all nine metapopulations as possible

sources (CAF, EAF, ESEA, Indonesia, PNG, SAM, SAS, WAF, WSEA), Asian strains (SAS, ESEA and PNG) accounted for less than 70% of the genetic makeup of Ebro1944, and the remaining was copied by the SAM strains[227].

Supplementary Note 8: Human Population Genomics of LDC020

As the radiocarbon dates obtained for the Laguna de los Cóndores individual LDC020 span the European contact period, we analyzed 1240k data to determine whether this individual exhibits any evidence of admixture with European populations. In a PCA constructed using a global set of populations from the SGDP, PC2 forms a cline separating Western Asian and European populations from East Asian, South American, and Oceanian populations. LDC020 clusters with modern populations from South America and is not shifted towards the European cluster, suggesting that this individual is of unadmixed Indigenous American ancestry (**Extended Data Figure 9**). A supervised ADMIXTURE analysis modeling the ancestry of LDC020 as a mixture of Atayal, French, Kalash, Karitiana, Mbuti, and Papuan further supports this result; LDC020 can be modeled as deriving 100% of its ancestry from Karitiana (**Extended Data Figure 9**). Finally, we tested these findings statistically using f_4-statistics of the form f_4(Mbuti.DG, Spanish.DG; Test, LDC020), where *Test* includes a set of South American populations from the Simons Genome Diversity Project. At three standard errors, none of the statistics differed significantly from 0; as such, LDC020 exhibits no evidence of European admixture (**Extended Data Figure 9**).

In a PCA only including relevant populations from South America, LDC020 overlaps with a modern Chachapoya individual while also falling close to modern Peruvians from the coast and Amazonians from Ecuador (**Extended Data Figure 9**). We explored further this clustering by using qpWave, which revealed that LDC020 is modeled as a sister clade to other Andean and Coastal populations (p value ≥ 0.05), and not to Amazonian groups (p value $\leq$ 0.005)(**Supplementary Table 16**).

Supplementary Note 9: High-Altitude Malaria- Epidemiological and Climatological Considerations

Modern climatological and environmental conditions strongly support the hypothesis that ancient *P. falciparum* from Chokhopani derived from an imported case rather than endemic high-altitude transmission. Situated in a high transverse valley at approximately 2800 masl, Chokhopani lies well above the altitudinal threshold for endemic transmission in the region[228], and entomological surveys in eastern Nepal have failed to document known malarial mosquito vector species above 1820 masl[229]. Furthermore, Chokhopani's mean annual temperature of c. 10°C lies well below the 16°C reported minimum required to sustain *P. falciparum* development, and low annual precipitation (~307 mm per year) makes the site unsuitable for vector survival and reproduction[19,50,229]. Today, *P. falciparum* accounts for a minority of Nepal's malaria cases, which are concentrated in the lowland Terai region bordering India[229]. Together, this data suggests that endemic malaria transmission at the site of Chokhopani would have been extremely unlikely; however, nearby lowland regions may have served as a source for imported infections. In fact, a significant proportion of Nepal's *P. falciparum* cases today appear to be imported from

India[51,229], and malarial cases have been observed at altitudes outside of endemic ranges; one illustrative case report documents an active *P. vivax* infection in a traveler in the Everest region at ~3900 masl[228].

Climatological conditions at Laguna de los Cóndores differ greatly from those of Chokhopani, making it impossible to completely exclude the possibility of high-altitude transmission of *P. vivax* at this location. The Chachapoyas territory, encompassing the eastern cordillera situated between the Marañón and Huallaga Rivers in the north-central Peruvian Andes, harbors a wide variety of ecotypes, including tropical zones which might have supported endemic malaria transmission. Average monthly temperatures at Laguna de los Cóndores, situated at ~2860 masl, range from 12-17°C[230], while the minimum temperature required for *P. vivax* reproduction inside the mosquito vector, T_{min}, is ~14.5°C[18]. Both this lower T_{min} and the ability to reactivate transmission from dormant liver forms known as hypnozoites enable *P. vivax* to persist in temperate climates unsuitable for *P. falciparum* transmission. Laguna de los Cóndores also experiences annual rainfall exceeding 3,200-4,000 mm[230]; extending another 700 meters above Laguna de los Cóndores, the cloud forest ecosystem provides a moist environment potentially conducive for reproduction of malaria vectors[231]. Indeed, entomological surveys in highland Ecuador in the 1940s documented the presence of the malaria vector *Anopheles pseudopunctipennis* at elevations as high as 2500-2700 masl, while recent reports suggest that this mosquito ranges up to 2800 and 3200 masl in Ecuador and Bolivia, respectively[232–235]. Isolated instances of high-altitude malaria epidemics in neighboring Andean countries further highlight the potential for endemic transmission at Laguna de los Cóndores; for instance, a 1998 epidemic outbreak of *Plasmodium vivax* struck several rural Bolivian villages situated at c. 2300 masl, with isolated cases documented as high as 3000 masl[236].

Past climatic oscillation and changes in land use complicate attempts to reconstruct historic disease ranges. Palynology, mineralogy, and diatom analysis of lake cores from Laguna de los Cóndores reveal a 2000-year history of climatic variability, with oscillation in precipitation levels leading to varied prevalence of maize cultivation around the site[230,231]. Even beyond these broad-scale climatic shifts, both interannual climate variability and shifts in microenvironments can have significant impacts on local malaria transmission cycles. Given these uncertainties, the likelihood of endemic *P. vivax* transmission at Laguna de los Cóndores in the pericontact period is difficult to ascertain.

Supplementary Note 10: Human Population Genomic Analysis of Individuals from Mechelen, Belgium

In this study, we present genome-wide data from 40 individuals from the St. Rombout's cemetery in the city of Mechelen (Belgium), spanning across three different phases from the 12th to the 18th c. CE. The genetic sex was determined to be male in 23 cases, with the highest proportion being in the middle and late phases (15th-18th c. AD; 13/15 males). Accordingly, all of the 8 individuals infected with malaria from these phases were determined to be males, and the only infected female dates to the first phase. Contamination estimated both from the X chromosome and the mitochondrial genome did not exceed 6%. The portion of SNPs from the 1240k panel covered after the enrichment protocol ('1240k capture') was more than 0.5M in 85% of the libraries. Thanks to this high overall coverage, a higher resolution in analyses like admixture modeling, genetic relatedness and assignment of uniparental haplogroups can be

expected. First, we did not detect any pairs of close relatives, and lcMLkin did not indicate that more distant relatives exist among our dataset either (**Supplementary Table 17**). When we project all the individuals from Mechelen on a W. Eurasian PCA along with other Medieval and Early Modern datasets from Europe, we find that those from the earliest phase of the cemetery -including the two malaria-infected individuals- cluster with published Late Medieval E. Germany and Netherlands ('Germany_Schleswig_Saxon_LMedieval', 'Netherlands_Groningen_Saxon_Medieval', respectively)[59], as well as present-day populations like 'French.HO', 'English.HO', and 'Scottish.HO'. In the absence of other contemporaneous data from Belgium, W. Germany, Netherlands, and France, we test these populations as a proxy for local Mechelen ancestry. We observe that some individuals from the middle and late phases are shifted away from the 'local' Mechelen-late Medieval-late Medieval E.Germany/Netherlands cluster, extending on a range of PC1-PC2 coordinates where Medieval western and southern Europe are positioned. Two individuals (STR199 and malaria-infected STR091) are found dispersed on a PC1-PC2 range where individuals from Medieval-Early modern C. Italy and Aegean Byzantine Anatolia are plotted. While individuals of 'non-local' ancestry are in both groups (infected; non-infected), we note that all four cases of co-infections from different malaria species had a non-local genetic profile. Deducing the most likely geographic origins of the infected individuals directly from the PCs has limitations, as the ancient genetic data -in their great majority- date to the early and late medieval periods when individual mobility was high. However, a recent study showed that the effective migration rate must have been lower, otherwise a collapse in the population structure would have been observed[135]. Considering that our context is associated to such a process i.e., individual mobilization for socio-political reasons, we applied *mobest*[136], a method that performs spatial interpolation of ancestry through time utilizing all ancient genetic data with spatiotemporal information. In this way, both migration and individual mobility traced through ancestry (i.e., 'genetic outlier') contributes to the ancestry field. We present the output of the software for each of the infected individuals in **Extended Data Figure 10** with their associated median date. The two infected individuals from the earliest phase (STR185 and STR248; c. 1100-1400 AD) display the highest similarity probabilities with N. Europe (present-day Germany, E. England and Scandinavia). As these are the oldest individuals in our dataset, the ancestry field was informed by Late Medieval data from Germany and Viking contexts. Accordingly, probabilities for STR129 and STR045 from the middle phase (*c.* 1400-1600 AD) are the highest for central/northern Europe, but also northern Balkans. From the same phase, STR025's and STR140's probabilities are shifted to France, but mostly to S. Spain and Sardinia. On the contrary, STR091 -one of the two cases co-infected with *P. malariae* and *P. falciparum*- exhibits highest similarity probabilities for central (present-day south Italy) and eastern Mediterranean (present-day Israel). We highlight that given the more recent date of these individuals, the lack of contemporaneous eastern Mediterranean data, and our kernel parameter *dt* set at *800y*, the extracted ancestry field is mainly informed by modern data. In the case of STR091, probability is higher for these two regions as southern Italians and Ashkenazi Jews (sampled in Israel) share very similar PC1 and PC2 coordinates with him. In the last phase (STR016, STR067, STR105; *c.* 1600-1800 AD), *mobest* supports equally well a local origin (present-day Belgium) as well as high similarity probabilities with S. Europe (southern Iberian peninsula, Sardinia and northern Balkans).

Our *qpWave* tests with Admixtools indicate that STR045, STR129 and STR248 are indistinguishable to the set of reference ('right') pops from Late Medieval Germany and Medieval Netherlands, as well as Vikings from Sweden (STR045 and STR129) (p value for one

stream of ancestry ≥ 0.05; **Supplementary Table 8**). In the same way, STR016, STR067 and STR140 are cladal to modern and Medieval Spain (‘Spanish.HO’ and/or ‘Spanish_North.HO’, ‘Spain_Medieval’ and/or ‘Spain_Carolingian’), and STR091 to Aegean Byzantine. For STR025, STR105 and STR185, we explored 2-way admixture combinations with *qpAdm*. Adequate models (i.e., p value ≥0.05) might represent unsampled genetic variation or recent admixture events. For example, STR105 can be modeled as a combination from Medieval Germany/Netherlands and Medieval Spain/Sardinia, however *qpWave* supports deriving 100% from modern Spanish_North.HO (*qpWave* p value=0.01). This could indicate the ancestry of STR105 is not well represented in the ancient DNA, but has survived into the modern population from northern Spain. Similarly, STR140 that can be modeled as deriving 100% from ‘Spain_Medieval’ or cladal to ‘Spanish.HO’ (mostly sampled from southern Spain), requires a second source from Guanches in Canary Islands when applying Medieval ‘Spain_Carolingian’ as first source. This can be explained by low amount of N. African-related ancestry in the group of ‘Spain_Medieval’ which could date back to the Late Antique period[134]. Therefore, our data do not support that STR140 carries additional N. African ancestry.

Individuals STR025 and STR185 are the only two that cannot be modeled as deriving 100% from any of the tested modern European populations. Using the Medieval groups, we could model female STR185 as 60% Germany and 40% Spain (‘Spain_Carolingian’,) and STR025 as 30% Germany and 70% Sardinia. In spite of the high genetic variation in autosomes, five of nine *Plasmodium*-infected males were assigned to the same derived haplogroup R1b1a1b1a1a2 (see table below), which is encountered in high frequency in western and north Europe since the Iron Age, and later among individuals from Viking contexts. Notably, individual STR016 belongs to the DF27 subclade of the R1b1a1b1a1a2 haplogroup, which is common among present-day Iberian populations and has been present in the region since the Bronze Age[237-239]. Overall, our results suggest a high correlation between PCA, *mobest* and *qpWave/qpAdm*. In most cases, *qpWave* narrows down the possible pools, and at the same time adequate models using ancient (medieval) or present-day sources pinpoint to the same geographic origin. Individuals STR025 and STR185 present exceptional cases, whereby we consider more complex models with admixture with potential sources from Spain and Sardinia, respectively.

Y-Haplogroups for malaria-positive individuals from Mechelen

Individual	Malaria Infection	Transect	Genetic Sex	Y-haplogroup
STR016	*falciparum/malariae*	17-18 (1600-1800)	M	R1b1a1b1a1a2a1b1a1a~
STR091	*falciparum/malariae*	15-16 (1400-1600)	M	R1b1a1b1a1a2
STR025	*falciparum/vivax*	15-16 (1400-1600)	M	H1a1a4b2a
STR140	*malariae*	15-16 (1400-1600)	M	J1a2a1a2d2b2b2c2a~
STR045	*vivax*	15-16 (1400-1600)	M	I1~
STR067	*vivax*	17-18 (1600-1800)	M	R1b1a1b1a1a2
STR105	*vivax*	17-18 (1600-1800)	M	R1b1a1b1a1a2c1a4b2
STR129	*vivax*	15-16 (1400-1600)	M	R1b1a1b1a1a2c1a1a1a1a1~
STR185	*vivax*	12-14 (1100-1400)	F	-
STR248	*vivax*	12-14 (1100-1400)	M	I2a1a1b1a1~

Supplementary Note 11: Factors Impacting *Plasmodium* spp. aDNA Recovery in High-Altitude Contexts

Whether endemic or imported, high-altitude *Plasmodium* spp. infections account for an extremely small proportion of malaria cases today[47]. Thus, the identification of aDNA from two different *Plasmodium* spp. in temporally and geographically divergent high-altitude settings warrants further consideration, and we offer several non-mutually exclusive hypotheses to explain this observation. First, previous work suggests that cold, dry environmental conditions characteristic of high-altitude sites may favor the recovery of ancient DNA. Previous studies support a thermal age model of cytosine deamination, with both sample date and ambient temperature influencing damage rates; ancient DNA fragmentation, on the other hand, appears to correlate with both precipitation and temperature fluctuation levels[240]. Consistent with this hypothesis, reports on both Chokhopani and Laguna de los Cóndores note exceptional preservation of organic materials[50,206], and remains from both sites show high rates of endogenous human DNA preservation[50,162].

In addition to favorable DNA preservation, the epidemiology of malarial infection itself may increase the likelihood of recovering ancient DNA from *Plasmodium* spp. at high altitude sites. As retrieval of pathogen DNA from the dental pulp chamber of ancient individuals is thought to reflect blood stream infection at the time of death[32], factors increasing the overall density of circulating pathogens may improve the likelihood of detection. In malaria cases, higher parasitemia is associated with severe disease and poor clinical outcomes, but geographical regions characterized by differing transmission dynamics display distinct patterns of age-specific disease burden[1,241]. Acquisition of clinical and antiparasitic immunity appears to rely on repeated, ongoing exposure to malaria parasites but may be quickly lost if the cycle of infection is interrupted. While adults in endemic regions with stable transmission experience nearly ubiquitous malarial infection, acquired immunity keeps circulating parasite densities low, and many experience asymptomatic infection[241]. Instead, immunologically naïve children under the age of 5 suffer the highest burden of malaria morbidity and mortality in holoendemic regions; however, poor preservation of fragile juvenile skeletal material and/or incomplete formation of the dentition may complicate retrieval of *Plasmodium* aDNA from young individuals[242]. Conversely, in regions prone to unstable transmission, all age groups are highly susceptible to infection by *Plasmodium* spp., with high parasitemia and mortality rates in adults during malaria epidemics. Thus, paradoxically, recovery of ancient *Plasmodium* spp. DNA from infected adults may be most likely in regions where malaria transmission is unstable, such as high-altitude sites on the fringes of endemic zones.

Finally, the physiology of human adaptation to high altitude hypoxia may impact malaria parasitemia and, consequently, the likelihood of detecting *Plasmodium* spp. infections in ancient individuals. At elevations exceeding 2500 masl, a reduction in the amount of inspired oxygen imposes a physiological stress, triggering several adaptive responses[243]. One such response includes an increase in erythropoiesis, the production of red blood cells[244]. In severe infections, *P. falciparum* may parasitize the majority of circulating RBCs; thus, it seems possible that an increase in total erythrocyte number could result in higher parasitemia, improving the likelihood of infection detection using aDNA. This hypothesis is supported by limited experimental data showing that ducks acclimatized to hypobaric conditions characteristic of high altitude displayed up to two-fold higher parasitemia than a control group after infection with *Plasmodium relictum*[245]. Interestingly, both the ancient Andean and Tibetan individuals included in this study

would be expected to experience an increase in total red blood cell count in response to high-altitude stress. The same is not true for modern Tibetans, who appear to have evolved a blunted erythropoietic response, instead displaying RBC and hemoglobin concentrations similar to populations at sea level[246]. The blunted erythropoietic response is linked to several Tibetan-specific variants in the *EPAS1* gene, part of the hypoxia inducible factor (HIF) pathway that controls the body's response to hypoxia[247]. Interestingly, previous analyses found that the Tibetan individual included in this study exhibited ancestral *EPAS1* alleles associated with the wild-type erythropoietic response to high altitude[50].

Supplementary Note 12: Malaria Preservation in Skeletal Material

Plasmodium spp. have a complex pathophysiology, with major life stages occurring in both the human bloodstream and the liver[4]. *P. falciparum*-infected red blood cells avoid splenic clearance by adhering to the epithelium in peripheral vasculature, contributing to diverse pathologies depending on the organ system(s) affected[4], while developmental *P. falciparum* and *P. vivax* gametyocytes are enriched in bone marrow[248]. Emerging research also suggests that certain *P. vivax* asexual stages may undergo sequestration in the bone marrow and liver[248]. This complexity inhibits efforts to identify *a priori* skeletal elements suitable for *Plasmodium* spp. DNA preservation in the archaeological record. Furthermore, unlike pathogens such as *Mycobacterium tuberculosis* and *Mycobacterium leprae*, *Plasmodium* spp. do not leave pathognomonic skeletal lesions suitable for the unambiguous identification of ancient malaria infections in the archaeological record[22,23]. As a result, in this study we opted for an unbiased metagenomic screening approach to identify capture candidates, analyzing previously-produced libraries for evidence of *Plasmodium* preservation regardless of skeletal element type.

We find that the dental pulp chamber provides a favorable microenvironment for the preservation of *Plasmodium* spp. DNA. Previous paleogenomic studies have succeeded in reconstructing genomes of numerous bacterial, viral, and eukaryotic pathogens from teeth, including partial mitochondrial genomes from *P. falciparum*[32,36]. The pulp chamber is highly vascularized, perhaps facilitating the retention of blood-stage *Plasmodium* parasites in dried material within the tooth[32,249]. Surprisingly, we also observe preservation of *Plasmodium* parasites in other skeletal elements, including the petrous portion of the temporal bone. Forming early in life, the highly dense otic capsule of the petrous bone resists taphonomic changes and provides favorable conditions for the preservation of host DNA[73]; however, the petrous bone has low vascularization and has been considered an unlikely candidate for pathogen preservation[132]. Interestingly, Kocher *et al.* recently succeeded in reconstructing numerous Hepatitis B Virus (HBV) genomes from petrous bones, although they show that teeth preserve HBV DNA significantly better than other skeletal elements[171].

We used a mixed effects model to explore the impact of skeletal element type on *Plasmodium* DNA preservation. When considering MT DNA, only the *Plasmodium* species had a significant effect on mean coverage, with an increased coverage for *P. falciparum* compared to *P. vivax* (p.value=0.0084). Consistent with this observation, *P. falciparum* exhibits ~20 copies of the mitochondrial genome per cell, while estimates of MT copy number for *P. vivax* range from c. 8-10[250]; furthermore, *P. falciparum* generally exhibits higher parasitemia compared to *P. vivax*[251]. On the other hand, while we observed an increase of MT coverage in libraries prepared from teeth instead of petrous bone, this difference did not appear significant (p.value=0.1998).

This could be due to the low number of petrous bones included in this study (7 petrous bones vs. 27 teeth), precluding detailed assessment. While factors facilitating pathogen preservation in the petrous bone remain to be elucidated, we suggest that variation in sampling practices may contribute. Archaeogenetic analyses typically target the dense otic capsule, but depending on sampling technique and precision, material from the more vascularized surrounding bone may also be collected. The crania also contains bone marrow, which might serve as a source for *Plasmodium* DNA. Further analysis of *Plasmodium* DNA from a variety of skeletal elements will be needed to explore differences in malaria preservation across tissue types.

Supplementary Note 13: Bayesian Molecular Dating Using BEAST

To explore the age of the Latin American and European *P. vivax* populations, we performed Bayesian molecular dating analyses using the software BEAST (Bayesian Evolutionary Analysis Sampling Trees, v. 2.7.6)[156]. First, following methods outlined elsewhere[30], we obtained raw data from a set of 15 modern *P. vivax* strains, including 12 from Latin America (Mexico and Colombia), as well as strains from Myanmar, North Korea, and India (**Supplementary Methods 12**). In order to compensate for the impact of recombination on clock rate estimation, previous analyses used the tool homoplasyFinder to identify and remove SNPs with patterns of presence/absence that contradict the tree topology (https://github.com/JosephCrispell/homoplasyFinder)[30,153]. Employing the same approach, we observed that filtering using homoplasyFinder differentially impacted branch length across clades in the phylogeny (**Extended Data Figure 6**). Notably, clades incorporating more modern strains exhibited increased branch shortening; this phenomenon is expected, as inclusion of additional sympatric samples increases the number of positions that contradict the tree topology. We note that application of homoplasyFinder resulted in removal of 5,123/17,100 positions in the present dataset (30.0%); consequently, we suggest that this approach to recombination removal may significantly bias clock-rate estimation. Instead, we selected a global set of samples from allopatric *P. vivax* populations for Bayesian molecular phylogenetic analysis (**Supplementary Methods 12**). We rationalized that geographically disparate, genetically distinct populations should have a limited history of admixture; although we cannot exclude an impact from ancient recombination events, we refrained from removing homoplastic SNPs in this dataset to avoid potential biases such approaches might introduce.

Next, we utilized root-to-tip regression and date randomization tests to check for the characteristics of a measurably-evolving population[155,252]. The root-to-tip regression performed using TempEst (v. 1.5.3) suggested limited evidence for temporal signal in our dataset (R^2=0.2946)[155]. To investigate this observation further, we used BEAST to perform 15 date-randomization trials under the optimized relaxed clock and coalescent Bayesian skyline models (**Supplementary Methods 12).** Tip dates were randomly shuffled and estimates of the mean clock rate compared to the results inferred using the true tip dates (**Supplementary Methods 12**). Notably, the mean uncorrelated log-normal relaxed clock rate with true dates (5.845×10^{-8}) falls within the 95% highest posterior density (HPD) interval for 5/15 date randomizations, while the 95% HPD interval for the mean uncorrelated log-normal relaxed clock rate with true dates (7.3211×10^{-9}, 1.2615×10^{-7}) overlaps the 95% HPD intervals estimated in 15/15 randomization tests (**Supplementary Table 18**). Therefore, we argue that the shallow sampling depth of our dataset provides limited temporal signal for inferring deep divergence

times in the *P. vivax* phylogeny.

Consistent with this observation, we note that BEAST infers an unexpectedly recent root date for our global set of *P. vivax* strains (**Extended Data Figure 6**); specifically, the median tree height of the maximum clade credibility tree inferred using the relaxed clock and bayesian skyline models is only 869.38 years old. This observation is consistent with the overall shape of the *P. vivax* phylogeny; modern strains exhibit long terminal branches compared to ancient clones, while internal branches are comparatively short (**Extended Data Figure 6**). However, this inferred recent origin of global *P. vivax* diversity is difficult to reconcile with the low-coverage ancient data produced in this study. Since our earliest *P. vivax* strains predate the root dates of our mcc trees by c. 5000 years, such an observation can only be explained as a complete replacement of the existing *P. vivax* diversity c. 1000 years ago. Moreover, despite low coverage, we show genetic similarity between ancient strains >1000 years old (e.g. Thebes, c. 3,000 years old) and modern strains from similar global regions, an observation which is difficult to explain under the complete replacement model. Therefore, we caution against overinterpretation of these results. Instead, we suggest that the (1.) limited temporal signal in our ancient datasets and (2.) contradictions between our inferred root age and ancient *P. vivax* population genetics results demonstrate that our dataset is insufficient to reliably date the ages of the Latin American and European *P. vivax* clades. A more thorough investigation of this question must await additional high-coverage samples from earlier times periods and may also necessitate application of alternative dating methods that can better accommodate the impact of diversity introduced through meiotic recombination.

SUPPLEMENTARY TEXT REFERENCES

102. Otto, T. D. *et al.* Long read assemblies of geographically dispersed *Plasmodium falciparum* isolates reveal highly structured subtelomeres [version 1; peer review: 3 approved]. *Wellcome Open Res* **3**, (2018).
103. Rutledge, G. G. *et al. Plasmodium malariae* and *P. ovale* genomes provide insights into malaria parasite evolution. *Nature* **542**, 101–104 (2017).
104. Shen, W., Le, S., Li, Y. & Hu, F. SeqKit: A Cross-Platform and Ultrafast Toolkit for FASTA/Q File Manipulation. *PLoS One* **11**, (2016).
105. Okonechnikov, K., Conesa, A. & García-Alcalde, F. Qualimap 2: advanced multi-sample quality control for high-throughput sequencing data. *Bioinformatics* **32**, 292–294 (2016).
106. Neukamm, J., Peltzer, A. & Nieselt, K. DamageProfiler: fast damage pattern calculation for ancient DNA. *Bioinformatics* **37**, 3652–3653 (2021).
107. Cock, P. J. A. *et al.* Biopython: freely available Python tools for computational molecular biology and bioinformatics. *Bioinformatics* **25**, 1422–1423 (2009).
108. Camponovo, F., Buckee, C. O. & Taylor, A. R. Measurably recombining malaria parasites. *Trends Parasitol.* **39**, 17–25 (2023).
109. Otto, T. D. *et al.* Genomes of all known members of a *Plasmodium* subgenus reveal paths to virulent human malaria. *Nat Microbiol* **3**, 687–697 (2018).
110. Virtanen, P. *et al.* SciPy 1.0: fundamental algorithms for scientific computing in Python. *Nat. Methods* **17**, 261–272 (2020).
111. Lamnidis, T. C. *et al.* Ancient Fennoscandian genomes reveal origin and spread of Siberian ancestry in Europe. *Nat. Commun.* **9**, (2018).
112. Ewels, P., Magnusson, M., Lundin, S. & Käller, M. MultiQC: summarize analysis results for multiple tools and samples in a single report. *Bioinformatics* **32**, 3047–3048 (2016).
113. Korneliussen, T. S., Albrechtsen, A. & Nielsen, R. ANGSD: Analysis of Next Generation Sequencing Data. *BMC Bioinformatics* **15**, (2014).
114. Fu, Q. *et al.* A revised timescale for human evolution based on ancient mitochondrial genomes. *Curr. Biol.* **23**, 553–559 (2013).
115. Arias, L. *et al.* Interpreting mismatches between linguistic and genetic patterns among speakers of Tanimuka (Eastern Tukanoan) and Yukuna (Arawakan). *Interface Focus* **13**, (2023).
116. Barbieri, C. *et al.* The Current Genomic Landscape of Western South America: Andes, Amazonia, and Pacific Coast. *Mol. Biol. Evol.* **36**, 2698–2713 (2019).
117. Bongers, J. L. *et al.* Integration of ancient DNA with transdisciplinary dataset finds strong support for Inca resettlement in the south Peruvian coast. *Proc. Natl. Acad. Sci. U. S. A.* **117**, 18359–18368 (2020).
118. Lazaridis, I. *et al.* Ancient human genomes suggest three ancestral populations for present-day Europeans. *Nature* **513**, 409–413 (2014).
119. Lindo, J. *et al.* The genetic prehistory of the Andean highlands 7000 years BP though European contact. *Sci Adv* **4**, (2018).
120. Moreno-Mayar, J. V. *et al.* Early human dispersals within the Americas. *Science* **362**, (2018).
121. Nakatsuka, N. *et al.* A Paleogenomic Reconstruction of the Deep Population History of the Andes. *Cell* **181**, 1131–1145 (2020).
122. Posth, C. *et al.* Reconstructing the Deep Population History of Central and South America. *Cell* **175**, 1185–1197 (2018).
123. Raghavan, M. *et al.* Genomic evidence for the Pleistocene and recent population history of Native Americans. *Science* **349**, (2015).
124. Rasmussen, M. *et al.* The genome of a Late Pleistocene human from a Clovis burial site in western Montana. *Nature* **506**, 225–229 (2014).
125. Scheib, C. L. *et al.* Ancient human parallel lineages within North America contributed to a coastal

expansion. *Science* **360**, 1024–1027 (2018).
126. Schroeder, H. *et al.* Origins and genetic legacies of the Caribbean Taino. *Proc. Natl. Acad. Sci. U. S. A.* **115**, 2341–2346 (2018).
127. Mallick, S. & Reich, D. The Allen Ancient DNA Resource (AADR): A curated compendium of ancient human genomes. Harvard Dataverse https://doi.org/10.7910/DVN/FFIDCW (2023).
128. Mallick, S. *et al.* The Allen Ancient DNA Resource (AADR) a curated compendium of ancient human genomes. *Sci Data* **11**, 182 (2024).
129. Antonio, M. L. *et al.* Ancient Rome: A genetic crossroads of Europe and the Mediterranean. *Science* **366**, 708–714 (2019).
130. Lazaridis, I. *et al.* The genetic history of the Southern Arc: A bridge between West Asia and Europe. *Science* **377**, (2022).
131. Marcus, J. H. *et al.* Genetic history from the Middle Neolithic to present on the Mediterranean island of Sardinia. *Nat. Commun.* **11**, (2020).
132. Margaryan, A. *et al.* Ancient pathogen DNA in human teeth and petrous bones. *Ecol. Evol.* **8**, 3534–3542 (2018).
133. Saag, L. *et al.* The Arrival of Siberian Ancestry Connecting the Eastern Baltic to Uralic Speakers further East. *Curr. Biol.* **29**, 1701–1711 (2019).
134. Olalde, I. *et al.* The genomic history of the Iberian Peninsula over the past 8000 years. *Science* **363**, 1230–1234 (2019).
135. Antonio, M. L. *et al.* Stable population structure in Europe since the Iron Age, despite high mobility. *Elife* **13**, (2024).
136. Schmid, C. & Schiffels, S. Estimating human mobility in Holocene Western Eurasia with large-scale ancient genomic data. *Proc. Natl. Acad. Sci. U. S. A.* **120**, (2023).
137. Skoglund, P. *et al.* Genetic evidence for two founding populations of the Americas. *Nature* **525**, 104–108 (2015).
138. Meyer, M. *et al.* A high-coverage genome sequence from an archaic Denisovan individual. *Science* **338**, 222–226 (2012).
139. Mallick, S. *et al.* The Simons Genome Diversity Project: 300 genomes from 142 diverse populations. *Nature* **538**, 201–206 (2016).
140. Bergström, A. *et al.* Insights into human genetic variation and population history from 929 diverse genomes. *Science* **367**, (2020).
141. Biagini, S. A. *et al.* People from Ibiza: an unexpected isolate in the Western Mediterranean. *Eur. J. Hum. Genet.* **27**, 941–951 (2019).
142. Lazaridis, I. *et al.* Genomic insights into the origin of farming in the ancient Near East. *Nature* **536**, 419–424 (2016).
143. Jeong, C. *et al.* The genetic history of admixture across inner Eurasia. *Nat Ecol Evol* **3**, 966–976 (2019).
144. Rohrlach, A. B. *et al.* Using Y-chromosome capture enrichment to resolve haplogroup H2 shows new evidence for a two-path Neolithic expansion to Western Europe. *Sci. Rep.* **11**, (2021).
145. Grubaugh, N. D. *et al.* An amplicon-based sequencing framework for accurately measuring intrahost virus diversity using PrimalSeq and iVar. *Genome Biol.* **20**, (2019).
146. Schönherr, S., Weissensteiner, H., Kronenberg, F. & Forer, L. Haplogrep 3 - an interactive haplogroup classification and analysis platform. *Nucleic Acids Res.* **51**, W263–W268 (2023).
147. Kennett, D. J. *et al.* Archaeogenomic evidence reveals prehistoric matrilineal dynasty. *Nat. Commun.* **8**, (2017).
148. Monroy Kuhn, J. M., Jakobsson, M. & Günther, T. Estimating genetic kin relationships in prehistoric populations. *PLoS One* **13**, (2018).
149. Rohrlach, A. B., Tuke, J., Popli, D. & Haak, W. BREADR: An R Package for the Bayesian Estimation of Genetic Relatedness from Low-coverage Genotype Data. *bioRxiv* (2023) doi:10.1101/2023.04.17.537144.

150. Lipatov, M., Sanjeev, K., Patro, R. & Veeramah, K. R. Maximum Likelihood Estimation of Biological Relatedness from Low Coverage Sequencing Data. *bioRxiv* (2015) doi:10.1101/023374.
151. Bates, D., Mächler, M., Bolker, B. & Walker, S. Fitting Linear Mixed-Effects Models Using lme4. *J. Stat. Softw.* **67**, 1–48 (2015).
152. Page, A. J. *et al.* SNP-sites: rapid efficient extraction of SNPs from multi-FASTA alignments. *Microb Genom* **2**, e000056 (2016).
153. Crispell, J., Balaz, D. & Gordon, S. V. HomoplasyFinder: a simple tool to identify homoplasies on a phylogeny. *Microb Genom* **5**, (2019).
154. Kozlov, A. M., Darriba, D., Flouri, T., Morel, B. & Stamatakis, A. RAxML-NG: a fast, scalable and user-friendly tool for maximum likelihood phylogenetic inference. *Bioinformatics* **35**, 4453–4455 (2019).
155. Rambaut, A., Lam, T. T., Max Carvalho, L. & Pybus, O. G. Exploring the temporal structure of heterochronous sequences using TempEst (formerly Path-O-Gen). *Virus Evol* **2**, (2016).
156. Bouckaert, R. *et al.* BEAST 2.5: An advanced software platform for Bayesian evolutionary analysis. *PLoS Comput. Biol.* **15**, (2019).
157. Douglas, J., Zhang, R. & Bouckaert, R. Adaptive dating and fast proposals: Revisiting the phylogenetic relaxed clock model. *PLoS Comput. Biol.* **17**, (2021).
158. Ramsey, C. B. Bayesian Analysis of Radiocarbon Dates. *Radiocarbon* **51**, 337–360 (2009).
159. Reimer, P. J. *et al.* The IntCal20 Northern Hemisphere Radiocarbon Age Calibration Curve (0–55 cal kBP). *Radiocarbon* **62**, 725–757 (2020).
160. Durand, R. Bourges (Cher). Monin, 33 rue Émile Martin. *Archéol. médiév.* **49**, 346–347 (2019).
161. Mazzarello, V. *et al.* Bioarchaeological analysis of Castelsardo's Mummies. *Ital J Anat Embryol* **119**, (2014).
162. Jeong, C. *et al.* Long-term genetic stability and a high-altitude East Asian origin for the peoples of the high valleys of the Himalayan arc. *Proc. Natl. Acad. Sci. U. S. A.* **113**, 7485–7490 (2016).
163. Varney, T. L. & Nicholson, D. V. Digging the grave of the Englishman: A preliminary report on excavations at a former British Navy hospital cemetery, English Harbour, Antigua, WI. in *Proceedings of the XVIII International Congress for Caribbean Archaeology, Grenada, WI* 329–335 (Association Internationale d'Archéologie de la Caraïbe, 1999).
164. Varney, T., Swanston, T., Brown, M. & Murphy, R. Exposed Burials on Galleon Beach, Antigua – a preliminary reporting. in *Proceedings of the XXVI International Congress for Caribbean Archaeology, Sint Maarten* (2015).
165. Karwowski, M. & Czubak, J. M. Das latènezeitliche Gräberfeld von Göttlesbrunn, Flur Geitzbilln (VF 07-08). in *Beiträge zum Tag der Niederösterreichischen Landesarchäologie 2019* (eds. Pieler, F. & Trebsche, P.) 71–78 (Bösmüller Print Management, 2019).
166. Cruz Berrocal, M. *et al.* A Comprised Archaeological History of Taiwan through the Long-Term Record of Heping Dao, Keelung. *Int. J. Hist. Archaeol.* **22**, 905–940 (2018).
167. Cruz Berrocal, M. *et al.* The Study of European Migration in Asia-Pacific During the Early Modern Period: San Salvador de Isla Hermosa (Keelung, Taiwan). *Int. J. Hist. Archaeol.* **24**, 233–283 (2020).
168. Abadie, I. L'évolution des pratiques funéraires religieuses des Parisiens: La fouille du cimetière de l'ancien hôpital médiéval et moderne de la Trinité. *Hist. Urbaine* **60**, 97–126 (2021).
169. Sekelj Ivančan, T. & Tkalčec, T. Archaeological site Torčec-Cirkvišće. *Podravina* **4**, 5–36 (2003).
170. Šlaus, M., Tkalčec, M. & Krznar, S. Paleodemography and paleopathology analyses of human osteology material from archeological site Torčec-Cirkvišče (near Koprivnica). *Podravina* **2**, 37–48 (2003).
171. Kocher, A. *et al.* Ten millennia of hepatitis B virus evolution. *Science* **374**, 182–188 (2021).
172. Gogâltan, F. & Ailincăi, S. C. Settlements of Life and Death. Studies from Prehistory to Middle Ages. in *Proceedings of an International Colloquium Tulcea, 25th-28th of May 2016* (2016).
173. Teschler-Nicola, M., Wiltschke-Schrotta, K., Prossinger, H. & Berner, M. The epidemiology of an Early Medieval population from Gars/Thunau, Lower Austria. *Homo* **45**, (1994).

174. Teschler-Nicola, M. Differential diagnosis of tuberculosis: the diagnostic value of endocranial features. in *The Evolution and Palaeoepidemiology of Tuberculosis (Abstractbook)* (eds. Pálfi, G., Dutour, O. & Deák, J.) (Szeged, 1997).
175. Latkoczy, C., Prohaska, T., Stingeder, G. & Teschler-Nicola, M. Strontium isotope ratio measurements in prehistoric human bone samples by means of high-resolution inductively coupled plasma mass spectrometry (HR-ICP-MS). *J. Anal. At. Spectrom.* **13**, 561–566 (1998).
176. Latkoczy, C., Prohaska, T., Stingeder, G. & Teschler-Nicola, M. Inductively Coupled Plasma Sectorfield Mass Spectrometry (ICP-SFMS) for Accurate and Precise Strontium Isotope Ratio Measurements in Prehistoric Human Bone Samples. in *Plasma Source Mass Spectrometry: State of the Art of ICP-MS* (eds. Holland, G. & Tanner, S. D.) 208–221 (The Royal Chemical Society, 1999).
177. Latkoczy, C., Prohaska, T., Watkins, M., Teschler-Nicola, M. & Stingeder, G. Strontium isotope ratio determination in soil and bone samples after on-line matrix separation by coupling ion chromatography (HPIC) to an inductively coupled plasma sector field mass spectrometer (ICP-SFMS). *J. Anal. At. Spectrom.* **16**, 806–811 (2001).
178. Huemer, C. Migration studies of humans by isotopic fingerprints using inductively coupled plasma mass spectrometry (ICP-MS). (University of Agricultural Sciences (BOKU), Vienna, 2008).
179. Pany-Kutschera, D. & Teschler-Nicola, M. Warriors versus working Men in Early Mediaeval Thunau – An Enthesis and Joint Study. (2008).
180. Rumpelmayr, K. Stabile-Isotopenuntersuchungen ($\delta^{13}C$ und $\delta^{15}N$) der frühmittelalterlichen Skelettreste von Thunau. (University Vienna, VERA-LAB, 2011).
181. Nittmann, J. Veränderungen der Wirbelsäule als Indikatoren physischer Belastung am Beispiel der frühmittelalterlichen Population von Gars/Thunau, Niederösterreich. (University of Vienna, 2012).
182. Nittmann, J. & Teschler-Nicola, M. Veränderungen der Wirbelsäule als Indikator physischer Belastung. *Mitt. Anthrop. Ges. Wien* **144**, 201–220 (2014).
183. Gangl, S. K. Autochthony in the Early Medieval settlement of Thunau/Kamp? A question explored by $^{87}Sr/^{86}Sr$ isotope ratios using MC ICP-MS. (University of Agricultural Sciences (BOKU), Vienna, 2014).
184. Teschler-Nicola, M. *et al.* The Early Mediaeval manorial estate of Gars/Thunau, Lower Austria: An enclave of endemic tuberculosis? *Tuberculosis* **95**, S51–S59 (2015).
185. Teschler-Nicola, M. *et al.* Die frühmittelalterlichen Fundstellen von Thunau am Kamp (NÖ) und ihre bioanthropologischen Evidenzen – eine Zusammenfassung. in *Archäologische Forschungen in Niederösterreich* (eds. Pieler, F. & Laussegger, A.) vol. 5 219–240 (Amt der Niederösterreichischen Landesregierung, 2018).
186. Friesinger, H. Beiträge zur Besiedlungsgeschichte des nördlichen Niederösterreich im 9.–10. Jahrhundert. *Archaeologia Austriaca* **37**, 79–113 (1965).
187. Friesinger, H. & Friesinger, I. Die Befestigungsanlage von Thunau. 5000 Jahre Siedlung im Garser Raum. *Katalogreihe des Krahuletzmuseums Eggenburg* **3**, (1975).
188. Friesinger, H. & Friesinger, I. Ein Vierteljahrhundert Grabungen in Thunau/Gars am Kamp. *Archäologie Österreichs* **2**, 6–22 (1991).
189. Obenaus, M., Breibert, W. & Szameit, E. Frühmittelalterliche Bestattungen und Siedlungsbefunde aus Thunau am Kamp, Niederösterreich – ein Vorbericht. *Fundberichte aus Österreich* **44**, 347–368 (2005).
190. Obenaus, M. Die neuen Forschungen in der frühmittelalterlichen Talsiedlung von Thunau am Kamp (Ein Zwischenbericht). *Studien zur Archäologie Europas* **14**, 529–549 (2011).
191. Obenaus, M. Die frühmittelalterliche Talsiedlung von Thunau am Kamp. Grabungen 2004 bis 2014. *Archäologie Österreichs* **26**, 9–21 (2015).
192. Stadler, P. *et al.* Die Absolutdatierung der urnenfelderzeitlichen und frühmittelalterlichen Wallanlagen von Thunau am Kamp, MG Gars am Kamp, Niederösterreich mit Hilfe von 14C-Daten. *Archaeologia Austriaca* **82/83**, 39–56 (1989/1999).
193. Herold, H. Der Schanzberg von Gars-Thunau in Niederösterreich. Eine befestigte Höhensiedlung mit

Zentralortfunktion aus dem 9.-10. Jahrhundert. *Archäologisches Korrespondenzblatt* **38**, 283–299 (2008).
194. Herold, H. The fortified hilltop site of Gars-Thunau and the settlements of the 9th and 10th centuries AD in Lower Austria. *Studien zur Archäologie Europas* **14**, 519–528 (2011).
195. Herold, H. & Szameit, E. Gars als Zentralort. in *Schicksalsjahr 907. Die Schlacht bei Pressburg und das frühmittelalterliche Niederösterreich. Ausstellungskatalog* (ed. Zehetmayer, R.) 87–91 (Niederösterreichisches Landesarchiv St. Pölten, 2007).
196. Nowotny, E. Mehrfachgräber im Gräberfeld von Thunau, Obere Holzwiese. Methodik, Ausprägung, Deutungsmöglichkeiten. *Archeologické rozhledy* **63**, 443–465 (2011).
197. Nowotny, E. Thunau am Kamp – Das frühmittelalterliche Gräberfeld auf der Oberen Holzwiese. in *Mitteilungen der Prähistorischen Kommission* vol. 87 (Verlag der Österr. Akademie der Wissenschaften – Wien, 2018).
198. Szameit, E. Gars-Thunau – frühmittelalterliche Residenz und vorstädtisches Handelszentrum. in *Burg – Burgstadt – Stadt. Zur Genese mittelalterlicher nichtagrarischer Zentren in Ostmitteleuropa* (ed. Brachmann, H.) 274–282 (Akademie Verlag Berlin, 1995).
199. Obenaus, M. Ostösterreich – Ein Grenzraum im 9. und 10. Jahrhundert aus archäologischer Sicht. Im Schnittpunkt frühmittelalterlicher Kulturen, Niederösterreich an der Wende vom 9. zum 10. Jahrhundert. *NÖLA-Mitteilungen aus dem Niederösterreichischen Landesarchiv* **3**, 194–218 (2008).
200. Schamall, D. *et al.* Structural analysis of a modern and a mediaeval case of *Actinomycosis* infection in pelvis and spine. *Wiener Medizinische Wochenschrift* **168**, 168 (2018).
201. Teschler-Nicola, M. *et al.* Reflections on corporal punishment and intoxication in the Early Mediaeval central site Gars/Thunau, Lower Austria (in prep).
202. Teschler-Nicola, M. & Winter, E. Bedeutungsvolle Steine Beispiele aus der Körpersteinkollektion der Pathologisch- anatomischen Sammlung im "Narrenturm"– Naturhistorisches Museum Wien (PASiN – NHM). *Archaeologie Österreichs* **24**, 43–48 (2013).
203. Vigil-Escalera Guirado, A. Gózquez. in *El poblamiento rural de época visigoda en Hispania. Arqueología del campesinado en el interior peninsular* (ed. Quirós Castillo, J. A.) 155–176 (Bilbao: UPV/EHU, 2013).
204. Vigil-Escalera Guirado, A. Invisible social inequalities in early medieval communities: the bare bones of household slavery. in *Social complexity in early medieval rural communities. The north-western Iberia archaeological record.* (ed. Quirós Castillo, J. A.) 113–124 (Archaeopress Publishing Ltd, Oxford, 2016).
205. Vigil-Escalera Guirado, A. *et al.* Productive strategies and consumption patterns in the Early Medieval village of Gózquez (Madrid, Spain). *Quat. Int.* **346**, 7–19 (2014).
206. von Hagen, A. & Guillén, S. TOMBS with a VIEW. *Archaeology* **51**, 48–54 (1998).
207. Espinoza, W. *Los Señoríos étnicos de Chachapoyas Y La Alianza Hispano-Chacha: Siglos XV-XVI*. 224–332 (Academia Nac. de la Historia, 1967).
208. Wild, E. M., Guillen, S., Kutschera, W., Seidler, H. & Steier, P. Radiocarbon dating of the Peruvian Chachapoya/Inca site at the Laguna de los Condores. *Nucl. Instrum. Methods Phys. Res. B* **259**, 378–383 (2007).
209. Küßner, M. Leubingen und Dermsdorf, Lkr. Sömmerda – „Fürstengrab", Großbau und Schatzdepot der frühen Bronzezeit. *Erfurt und Umgebung. Archäologische Denkmale in Thüringen* **3**, 194–197 (2014).
210. Kokles, A.-K. Baalberge in Thüringen: Zwei neu entdeckte Trapezgrabenanlagen sowie Siedlungsbefunde aus Leubingen, Lkr. Sömmerda. *Alteuropäische Forschungen* **9**, 83–98 (2016).
211. Damašek, L. *et al.* Archaeological excavations at Lungi Tepa, south Uzbekistan. Preliminary Report for Season 2019. *Studia Hercynia* **2020**, 159–178 (2020).
212. Papac, L. *et al.* Dynamic changes in genomic and social structures in third millennium BCE central Europe. *Sci Adv* **7**, (2021).
213. Ernée, M. *et al. Mikulovice. Early Bronze Age Cemetery on the Amber Road*. (Památky

archeologické, Supplementum 21, Prague, Institute of Archaeology, 2021).
214. Posth, C. *et al.* The origin and legacy of the Etruscans through a 2000-year archeogenomic time transect. *Sci. Adv.* **7**, (2021).
215. Shaw, I. *The Oxford History of Ancient Egypt*. (Oxford University Press, Oxford ; New York, 2003).
216. Wilkinson, T. *The Rise and Fall of Ancient Egypt*. (Random House, New York, 2010).
217. Schmidt, E. Catalog der im Anatomischen Institut der Universität Leipzig aufgestellten craniologischen Sammlung des Herrn Dr. Emil Schmidt. Die Anthropologischen Privat-Sammlungen Deutschlands. Preprint at (I. F. Vieweg und Sohn 1887).
218. Schmidt, E. Ueber alt- und neuägyptische Schädel. Beitrag zu unseren Anschauungen ueber die Veränderlichkeit und Constanz der Schädelformen. Der Philosophischen Facultät der Universität Leipzig vorgelegt zum Zweck der Habilitation für Anthropologie. (1885).
219. Németh, R. E. The Middle Bronze Age 'Mass Grave' from Voivodeni- La Școală. A Chronological Approach. in *Bronze Age Chronology in the Carpathian Basin: Proceedings from the International Colloquium from Târgu Mureș* (eds. Németh, R. E. & Rezi, B.) 179–199 (MEGA, 2015).
220. Spitsyn, A. A. Kurgans of the Saint Petersburg province in the excavations of L.K. Ivanovsky. in *Materials on the Archaeology of Russia* vol. 20 123 (GDE Printing House, Saint Petersburg, 1896).
221. Lesman, Y. M. Chronological Periodization of the Izhora Plateau Barrows. in *Northern Russia and its Neighbors in the Early Middle Ages* (ed. Stolyar, A. D.) 65–74 (LSU Publ., Leningrad, 1982).
222. Седов, В. В. Этнический состав населения северо-западных земель Великого Новгорода (IX-XIV вв.). *Советская Археология* **XVIII**, 190–229 (1953).
223. Patrushev, V. & Lavento, M. Sosnovaya Griva 3- A Dwelling Site Complex in the Mari Republic, in the Middle Volga Region. *Fennoscandia archaeological XIII* 29–49 (1996).
224. McCormick, M. Tracking mass death during the fall of Rome's empire (I). *Journal of Roman Archaeology* **28**, 325–357 (2015).
225. Schuenemann, V. J. *et al.* Ancient Egyptian mummy genomes suggest an increase of Sub-Saharan African ancestry in post-Roman periods. *Nat. Commun.* **8**, 15694 (2017).
226. Lawson, D. J., Hellenthal, G., Myers, S. & Falush, D. Inference of Population Structure using Dense Haplotype Data. *PLoS Genet.* **8**, e1002453 (2012).
227. Hellenthal, G. *et al.* A genetic atlas of human admixture history. *Science* **343**, 747–751 (2014).
228. Bishop, R. A. & Litch, J. A. Malaria at High Altitude. *J. Travel Med.* **7**, 157–158 (2006).
229. Dhimal, M., Ahrens, B. & Kuch, U. Altitudinal shift of malaria vectors and malaria elimination in Nepal. *Malar. J.* **13**, (2014).
230. Matthews-Bird, F., Valencia, B. G., Church, W., Peterson, L. C. & Bush, M. A 2000-year history of disturbance and recovery at a sacred site in Peru's northeastern cloud forest. *Holocene* **27**, 1707–1719 (2017).
231. Åkesson, C. M. *et al.* 2,100 years of human adaptation to climate change in the High Andes. *Nat Ecol Evol* **4**, 66–74 (2020).
232. Pinault, L. L. & Hunter, F. F. Malaria in Highlands of Ecuador since 1900. *Emerg. Infect. Dis.* **18**, 615–622 (2012).
233. Pinault, L. L. & Hunter, F. F. New highland distribution records of multiple *Anopheles* species in the Ecuadorian Andes. *Malar. J.* **10**, (2011).
234. Levi-Castillo, R. *Anopheles pseudopunctipennis* in the Los Chillos Valley of Ecuador. *J. Econ. Entomol.* **38**, 385–388 (1945).
235. Lardeux, F., Loayza, P., Bouchité, B. & Chavez, T. Host choice and human blood index of *Anopheles pseudopunctipennis* in a village of the Andean valleys of Bolivia. *Malar. J.* **6**, (2007).
236. Rutar, T., Baldomar Salgueiro, E. J. & Maguire, J. H. Introduced *Plasmodium vivax* Malaria in a Bolivian Community at an Elevation of 2,300 Meters. *Am. J. Trop. Med. Hyg.* **70**, 15–19 (2004).
237. Roewer, L. *et al*. Signature of recent historical events in the European Y-chromosomal STR haplotype distribution. *Hum. Genet.* **116**, 279–291 (2005).
238. García-Fernández, C. *et al.* Y-chromosome target enrichment reveals rapid expansion of haplogroup

R1b-DF27 in Iberia during the Bronze Age transition. *Sci. Rep.* **12**, (2022).
239. Solé-Morata, N. *et al.* Analysis of the R1b-DF27 haplogroup shows that a large fraction of Iberian Y-chromosome lineages originated recently in situ. *Sci. Rep.* **7**, (2017).
240. Kistler, L., Ware, R., Smith, O., Collins, M. & Allaby, R. G. A new model for ancient DNA decay based on paleogenomic meta-analysis. *Nucleic Acids Res.* **45**, 6310–6320 (2017).
241. Doolan, D. L., Dobaño, C. & Baird, J. K. Acquired Immunity to Malaria. *Clin. Microbiol. Rev.* **22**, 13–36 (2009).
242. AlQahtani, S. J., Hector, M. P. & Liversidge, H. M. Brief Communication: The London Atlas of Human Tooth Development and Eruption. *Am. J. Phys. Anthropol.* **142**, 481–490 (2010).
243. Bigham, A. W. *et al.* Andean and Tibetan Patterns of Adaptation to High Altitude. *Am. J. Hum. Biol.* **25**, 190–197 (2013).
244. Storz, J. F. & Scott, G. R. Life Ascending: Mechanism and Process in Physiological Adaptation to High-Altitude Hypoxia. *Annu. Rev. Ecol. Evol. Syst.* **50**, 503–526 (2019).
245. Rostorfer, H. H. & Rigdon, R. H. ANOXIA IN MALARIA: An Experimental Study on Ducks. *J. Lab. Clin. Med.* **30**, 860–866 (1945).
246. Beall, C. M. Andean, Tibetan, and Ethiopian patterns of adaptation to high-altitude hypoxia. *Integr. Comp. Biol.* **46**, 18–24 (2006).
247. Storz, J. F. High-Altitude Adaptation: Mechanistic Insights from Integrated Genomics and Physiology. *Mol. Biol. Evol.* **38**, 2677–2691 (2021).
248. Obaldia, N., 3rd *et al.* Bone Marrow Is a Major Parasite Reservoir in Plasmodium vivax Infection. *MBio* **9**, (2018).
249. Bos, K. I. *et al.* Paleomicrobiology: Diagnosis and Evolution of Ancient Pathogens. *Annu. Rev. Microbiol.* **73**, 639–666 (2019).
250. Sharma, I. & Sharma, Y. D. Malaria Mitochondrial Genome: The 6kb Element. *Indian J. Malariol.* **38**, 45–60 (2001).
251. Menkin-Smith, L. & Winders, W. T. *Plasmodium Vivax Malaria*. (StatPearls Publishing, 2023).
252. Duchêne, S., Duchêne, D., Holmes, E. C. & Ho, S. Y. W. The Performance of the Date-Randomization Test in Phylogenetic Analyses of Time-Structured Virus Data. *Mol. Biol. Evol.* **32**, 1895–1906 (2015).
