# Supplementary material for: Ancient Plasmodium genomes shed light on the history of human malaria
Source: Nature. 2024 Jun 12;631(8019):125–33. doi: 10.1038/s41586-024-07546-2 (PMC11222158; doi:10.1038/s41586-024-07546-2)
Supplement: Supplementary file 2 — Reporting Summary [file 41586_2024_7546_MOESM2_ESM.pdf]

nature portfolio

Corresponding author(s): Megan Michel, Alexander Herbig, Johannes Krause

Last updated by author(s): 04/19/2024

Reporting Summary

Nature Portfolio wishes to improve the reproducibility of the work that we publish. This form provides structure for consistency and transparency in reporting. For further information on Nature Portfolio policies, see our Editorial Policies and the Editorial Policy Checklist.

Statistics

For all statistical analyses, confirm that the following items are present in the figure legend, table legend, main text, or Methods section.

n/a	Confirmed	
☐	☒	The exact sample size (*n*) for each experimental group/condition, given as a discrete number and unit of measurement
☒	☐	A statement on whether measurements were taken from distinct samples or whether the same sample was measured repeatedly
☐	☒	The statistical test(s) used AND whether they are one- or two-sided<br>*Only common tests should be described solely by name; describe more complex techniques in the Methods section.*
☒	☐	A description of all covariates tested
☒	☐	A description of any assumptions or corrections, such as tests of normality and adjustment for multiple comparisons
☐	☒	A full description of the statistical parameters including central tendency (e.g. means) or other basic estimates (e.g. regression coefficient) AND variation (e.g. standard deviation) or associated estimates of uncertainty (e.g. confidence intervals)
☐	☒	For null hypothesis testing, the test statistic (e.g. *F*, *t*, *r*) with confidence intervals, effect sizes, degrees of freedom and *P* value noted<br>*Give P values as exact values whenever suitable.*
☐	☒	For Bayesian analysis, information on the choice of priors and Markov chain Monte Carlo settings
☒	☐	For hierarchical and complex designs, identification of the appropriate level for tests and full reporting of outcomes
☒	☐	Estimates of effect sizes (e.g. Cohen's *d*, Pearson's *r*), indicating how they were calculated

Our web collection on statistics for biologists contains articles on many of the points above.

Software and code

Policy information about availability of computer code

Data collection	No software was used in data collection.
Data analysis	The following pieces of published/publicly available software were used in the analysis of data presented in this manuscript: AdapterRemoval (v. 2.3.2), AdmixTools (v. 7.0.2), ADMIXTURE (v. 1.3.0), AdmixturePlotter (https://github.com/TCLamnidis/AdmixturePlotter), AMDirT (v. 1.3, https://amdirt.readthedocs.io/en/latest/), ANGSD (v. 0.935), BamUtil (v. 1.0.15), Bayesian Evolutionary Analysis Sampling Trees 2 (BEAST2, v. 2.7.6), BEAUti (v. 2.7.6), BEDtools (v. 2.25.0 or v. 2.30.0), BLAST (https://blast.ncbi.nlm.nih.gov/Blast.cgi), Biopython (Bio.Entrez package, Biopython version 1.79), BREAD (), BWA aln (v. 0.7.12 or v. 0.7.17), ContamMix (v. 1.0-10), Chromopainter/fineSTRUCTURE (v. 2), DamageProfiler (v. 0.4.9 or v. 1.1), dustmasker (v. 1.0.0, from BLAST package 2.9.0), EIGENSOFT (v. 7.2.1), fastp (v. 0.20.1), FASTQC (v. 0.11.9, https://www.bioinformatics.babraham.ac.uk/projects/fastqc/), FigTree (v. 1.4.4), GATK UnifiedGenotyper (v. 3.5), GenoSL (https://github.com/aidaanva/GenoSL), Haplogrep3 (v. 3.2.1), Heuristic Operations for Pathogen Screening (HOPS, v. 0.35) pipeline, homoplasyFinder (https://github.com/JosephCrispell/homoplasyFinder), Interactive Tree of Live (iTOL, v. 6.7.6), ivar (v. 1.3), LcMLkin (v.), leeHom (v. 1.1.5-eb382b3 or v. 1.1.5-ba378b6), lme4 (v. 1.1-34), MarkDuplicates (v. 2.26.0, http://broadinstitute.github.io/picard), MDF.R (https://github.com/aidaanva/MDF), MEGA-CC (v. 10.0.2), MEGAN Alignment Tool (MALT, v. 0.4.0, v. 0.4.1, or v. 0.5.2), MEGAN (MEtaGenome ANalyzer, v. 6.25.3), mobest (v. 1.0.0, https://github.com/nevrome/mobest/releases), MultiQC (v. 1.3), MultiVCFAnalyzer (v. 0.85.2 and v. 0.87.1), nf-core/eager (v. 2.3.1, v. 2.4.5, or v. 2.4.6), OxCal (v. 4.4), picard AddOrReplaceReadGroups (v. 2.18.29-SNAPSHOT, http://broadinstitute.github.io/picard), pileupcaller (v. 1.5.2), PLINK (v. 1.90, http://pngu.mgh.harvard.edu/purcell/plink/), pMMRCalculator (v. 1.1.0, https://github.com/TCLamnidis/pMMRCalculator), PMR (), PopART (http://popart.otago.ac.nz), Poseidon (v. 2.7.1, http://www.poseidon-adna.org), PRINSEQ-lite (v. 0.20.4), PRINSEQ parallel (https://github.com/spabinger/prinseq_parallel), ProbeGenerator (v. 0.89), qpAdm (v.)5.1, qpWave (v. 5.1), Qualimap (v. 2.2.2), randomise_dates_beast2.py (https://github.com/sebastianduchene/phylo_xml_tools), RAxML-NG (v. 1.1), READ (v.), Samtools (v. 1.3, v. 1.9, or v. 1.12), SciPy (v. 1.9), SeqKit (v. 2.4.0), seqtk (v. 1.2-r94), Sex.DetERRmine (v 1.1.2), smartPCA (v. 16000), SNP-sites (v. 2.5.1), TempEst (v. 1.5.3), Tracer (v. 1.7.2), TreeAnnotator (v. 2.7.6).

Custom scripts used for data processing and/or analysis can be retrieved from https://github.com/meganemichel/plasmodium_project_scripts.

For manuscripts utilizing custom algorithms or software that are central to the research but not yet described in published literature, software must be made available to editors and reviewers. We strongly encourage code deposition in a community repository (e.g. GitHub). See the Nature Portfolio guidelines for submitting code & software for further information.

Data

Policy information about availability of data

All manuscripts must include a data availability statement. This statement should provide the following information, where applicable:

- Accession codes, unique identifiers, or web links for publicly available datasets
- A description of any restrictions on data availability
- For clinical datasets or third party data, please ensure that the statement adheres to our policy

Raw sequencing data from 36 malaria-positive individuals as well as newly-reported data from 41 ancient individuals enriched at human ancestry-informative SNP positions have been deposited on the European Nucleotide Archive (ENA) (Accession number PRJEB73276). Ancient and modern P. vivax and P. falciparum nuclear genotypes are available in eigenstrat format (https://figshare.com/projects/Ancient_Plasmodium_genomes_shed_light_on_the_history_of_human_malaria/196711). This study utilizes modern P. falciparum genotype datasets available through the Pf6 data release of the MalariaGEN P. falciparum Community Project (ftp://ngs.sanger.ac.uk/production/malaria/pfcommunityproject/Pf6/). Modern P. vivax genotype datasets analyzed here are available through the Pv4 data release of the MalariaGEN P. vivax Genome Variation project (ftp://ngs.sanger.ac.uk/production/malaria/Resource/30). Previously published raw sequencing datasets from Indian P. falciparum strains and the Ebro Delta blood slide can be obtained from the European Nucleotide Archive under accession numbers PRJNA322219 and PRJEB30878, respectively. This study utilized previously published ancient human genotype datasets obtained from the Reich Laboratory's Allen Ancient DNA Resource v. 54.1 (https://reich.hms.harvard.edu/allen-ancient-dna-resource-aadr-downloadable-genotypes-present-day-and-ancient-dna-data). Previously published modern P. falciparum and P. vivax mitochondrial datasets as well as genomic sequences utilized in our probe design and metagenomic screening database are available from the National Center for Biotechnology Information (NCBI) (accession numbers in Supplementary Tables 9, 10, 11, and 13). The following whole-genome sequencing datasets available on the NCBI Sequence Read Archive were used for phylogenetic dating: SAMN02677154, SAMN02677164, SAMN02677167, SAMN03274512, SAMN02677169, SAMN02677170, SAMN02677171, SAMN02677180, SAMN02677183, SAMN02677184, SAMN02677185, SAMN02677186, SAMN02677187, SAMN02677195, SAMN00710542. Finally, the following publicly available genomes are available on NCBI and were used as references in this study: P. falciparum mitochondria: LR605957.1, P. vivax mitochondria: LT635627.1, P. malariae mitochondria: LT594637.1, P. falciparum nuclear chromosomes: GCA_000002765.3, P. vivax nuclear chromosomes: GCA_900093555.1, P. vivax-like nuclear chromosomes: GCA_003402215.1, Plasmodium cynomolgi nuclear chromosomes: GCA_900180395.1, P. praefalciparum nuclear chromosomes: GCA_900095595.1, and the Genome Reference Consortium Human Build 37 (HG19): PRJNA31257.

Research involving human participants, their data, or biological material

Policy information about studies with human participants or human data. See also policy information about sex, gender (identity/presentation), and sexual orientation and race, ethnicity and racism.

Reporting on sex and gender	All references to the sex of ancient individuals refers to biological sex, as determined based on osteological and/or genetic analysis.
Reporting on race, ethnicity, or other socially relevant groupings	NA
Population characteristics	NA
Recruitment	NA
Ethics oversight	NA

Note that full information on the approval of the study protocol must also be provided in the manuscript.

Field-specific reporting

Please select the one below that is the best fit for your research. If you are not sure, read the appropriate sections before making your selection.

☒ Life sciences ☐ Behavioural & social sciences ☐ Ecological, evolutionary & environmental sciences

For a reference copy of the document with all sections, see nature.com/documents/nr-reporting-summary-flat.pdf

Life sciences study design

All studies must disclose on these points even when the disclosure is negative.

Sample size	No sample size calculation was performed in association with this study. Instead, we used a metagenomic approach to identify candidate libraries preserving ancient Plasmodium DNA and captured all those exceeding preservational thresholds.
Data exclusions	No data was excluded from this study.

Replication	For population genetic analyses such as ADMIXTURE and phylogenetics, we used bootstrap replicates to infer confidence estimates for inferred relationships.
Randomization	For the Bayesian phylogenetic dating, we performed a date randomization test to compare the mutation rates estimated using randomly shuffled dates compared to true sampling dates for Plasmodium vivax strains. Out of 15 replicate BEAST analyses with shuffled dates, all 15 yielded mutation rate estimates with a 95% highest posterior density interval overlapping the 95% highest posterior density interval inferred using true dates, indicating that there is insufficient temporal signal in the dataset to reliably date divergence events.
Blinding	No blinding was performed in the present study. We performed only quantitative genomic analyses which are less vulnerable to subjective biases. Furthermore, as the samples analyzed derive from ancient archaeological remains that exhibited some evidence of malaria infection, they could not be allocated into experimental groups for which blinding would be an appropriate experimental design.

Reporting for specific materials, systems and methods

We require information from authors about some types of materials, experimental systems and methods used in many studies. Here, indicate whether each material, system or method listed is relevant to your study. If you are not sure if a list item applies to your research, read the appropriate section before selecting a response.

Materials & experimental systems

n/a	Involved in the study	
☒	☐	Antibodies
☒	☐	Eukaryotic cell lines
☐	☒	Palaeontology and archaeology
☒	☐	Animals and other organisms
☒	☐	Clinical data
☒	☐	Dual use research of concern
☒	☐	Plants

Methods

n/a	Involved in the study	
☒	☐	ChIP-seq
☒	☐	Flow cytometry
☒	☐	MRI-based neuroimaging

Palaeontology and Archaeology

Specimen provenance	Samples were obtained with prior permission from the appropriate authority in each case and following legal guidelines in the relevant country/countries.
Specimen deposition	Sequences/genotypes associated with this project have been deposited on the ENA and/or figshare to facilitate access by other researchers.
Dating methods	New dates were generated by AMC C14 dating at the Mannheim lab. All dates were recalibrated using the OxCal software (IntCal20 calibration curve).

☒ Tick this box to confirm that the raw and calibrated dates are available in the paper or in Supplementary Information.

Ethics oversight	Prehistoric and historic individuals, therefore no ethical oversight was required.

Note that full information on the approval of the study protocol must also be provided in the manuscript.

Plants

Seed stocks	*Report on the source of all seed stocks or other plant material used. If applicable, state the seed stock centre and catalogue number. If plant specimens were collected from the field, describe the collection location, date and sampling procedures.*
Novel plant genotypes	*Describe the methods by which all novel plant genotypes were produced. This includes those generated by transgenic approaches, gene editing, chemical/radiation-based mutagenesis and hybridization. For transgenic lines, describe the transformation method, the number of independent lines analyzed and the generation upon which experiments were performed. For gene-edited lines, describe the editor used, the endogenous sequence targeted for editing, the targeting guide RNA sequence (if applicable) and how the editor was applied.*
Authentication	*Describe any authentication procedures for each seed stock used or novel genotype generated. Describe any experiments used to assess the effect of a mutation and, where applicable, how potential secondary effects (e.g. second site T-DNA insertions, mosiacism, off-target gene editing) were examined.*
